# Dynamic transcriptome profiling dataset of vaccinia virus obtained from long-read sequencing techniques

**DOI:** 10.1093/gigascience/giy139

**Published:** 2018-11-23

**Authors:** Dóra Tombácz, István Prazsák, Attila Szűcs, Béla Dénes, Michael Snyder, Zsolt Boldogkői

**Affiliations:** 1 Department of Medical Biology, Faculty of Medicine, University of Szeged, Somogyi B. u. 4., 6720 Szeged, Hungary; 2 Veterinary Diagnostic Directorate of the National Food Chain Safety Office, Tábornok u. 2., 1143 Budapest, Hungary; 3 Department of Genetics, School of Medicine, Stanford University, 300 Pasteur Dr, Stanford, California, USA

**Keywords:** poxvirus, vaccinia virus, long-read sequencing, full-length transcriptome, Pacific Biosciences, RS II system, Sequel system, Oxford Nanopore Technologies, MinION system, direct RNA sequencing

## Abstract

**Background:**

Poxviruses are large DNA viruses that infect humans and animals. Vaccinia virus (VACV) has been applied as a live vaccine for immunization against smallpox, which was eradicated by 1980 as a result of worldwide vaccination. VACV is the prototype of poxviruses in the investigation of the molecular pathogenesis of the virus. Short-read sequencing methods have revolutionized transcriptomics; however, they are not efficient in distinguishing between the RNA isoforms and transcript overlaps. Long-read sequencing (LRS) is much better suited to solve these problems and also allow direct RNA sequencing. Despite the scientific relevance of VACV, no LRS data have been generated for the viral transcriptome to date.

**Findings:**

For the deep characterization of the VACV RNA profile, various LRS platforms and library preparation approaches were applied. The raw reads were mapped to the VACV reference genome and also to the host (*Chlorocebus sabaeus*) genome. In this study, we applied the Pacific Biosciences RSII and Sequel platforms, which altogether resulted in 937,531 mapped reads of inserts (1.42 Gb), while we obtained 2,160,348 aligned reads (1.75 Gb) from the different library preparation methods using the MinION device from Oxford Nanopore Technologies.

**Conclusions:**

By applying cutting-edge technologies, we were able to generate a large dataset that can serve as a valuable resource for the investigation of the dynamic VACV transcriptome, the virus-host interactions, and RNA base modifications. These data can provide useful information for novel gene annotations in the VACV genome. Our dataset can also be used to analyze the currently available LRS platforms, library preparation methods, and bioinformatics pipelines.

## Data Description

### Background

*Poxviridae* is a large virus family that infects vertebrates and invertebrates with highly pathogenic members, such as the Variola virus, which is the causative agent of smallpox [1]. Vaccinia virus (VACV) is the prototypic member of the Orthopoxvirus genus. It is closely related to the *Variola virus* [2] that was eradicated as a result of a successful global vaccination program using live VACV.

It had generally been assumed that the virus in the smallpox vaccine, renamed vaccinia virus, is a cowpox virus. However, VACV differs from the cowpox virus and has no known natural hosts; its origin is still being investigated. It has been suggested that the smallpox vaccine was based on horsepox [3]. VACV has been extensively utilized as an expression and a gene delivery vector [4]. It also serves as a model system for the analysis of virus-host interactions, transcriptional regulation, and for other molecular biological studies [5].

Poxviruses are able to replicate in the cytoplasm of the host cell because they encode the proteins needed for DNA synthesis [6]. They have a relatively large (approximately 195 kbp) double-stranded DNA genome coding for about 220 proteins. The VACV genes are divided into three temporal classes: early (E), intermediate (I), and late (L) genes. A study characterized 35 VACV genes as immediate-early (IE) kinetics [7], but this categorization has not been widely accepted. The promoters of genes belonging to different kinetic classes are recognized by stage-specific transcription factors [8–11]. VACV genes belonging to the same kinetic group have been shown to be clustered in the genome [7]: E genes are located at the termini of the viral genome, while I and L genes are situated in the middle genomic region. Most of the adjacent VACV genes are oriented in the same direction, while convergent and divergent positioning is uncommon.

Although the extraordinary complexity of the VACV transcriptome has been thought to be well characterized [12–15], traditionally used techniques such as short-read sequencing (SRS), ribosome profiling, cap analysis of gene expression (CAGE), and genome tiling [16] are not able to span the entire transcript nor to distinguish between transcript isoforms, bi-, and polycistronic RNA variants, overlapping gene products, and embedded RNAs. Transcriptional overlaps generated by the read-through mechanism are very frequent in VACV and cause a major problem in the analysis of individual viral transcripts using traditional approaches. The transcription patterns of VACV genes exhibit an extreme stochasticity, which includes an enormous number of transcriptional start sites (TSSs) and transcription end sites (TESs) even within the open reading frames (ORFs). These features of transcription are uncommon even among large DNA viruses; it might represent a form of gene regulation that is unique to living organisms. Therefore, it is especially important to use full-length sequencing methods in order to match the transcript ends.

Previous studies have determined the precise TSSs and TESs of VACV transcripts [14, 17]. However, the methods that were applied were not suitable for detecting the entire transcripts at the single-molecule level, and therefore it was impossible to determine which TSSs are paired by certain TESs.

The Pacific Biosciences (PacBio) isoform sequencing (Iso-Seq) protocol (using oligo(dT) or random hexamer primers for the reverse transcription), the cDNA sequencing, and direct (d)RNA-sequencing (RNA-seq) methods from the Oxford Nanopore Technologies (ONT), as well as the Cap-selection (Cap-Seq) cDNA preparation method (Lexogen) are able to generate full-length transcripts, and thus they can circumvent the limitations of SRS techniques. By using these techniques for cDNA productions and library preparations with the PacBio Real-Time Sequencer (RS)II and Sequel, as well as the ONT MinION platforms, we were able to identify hundreds of novel RNA isoforms (e.g., TSS and TES variants, mono-, bi-, polycistronic transcripts), dozens of coding and non-coding RNAs, and numerous complex transcripts in various herpesviruses [18–23] and in a baculovirus [24] and we were also able to generate a comprehensive full-length transcript data catalog of VACV.

**Figure 1:**
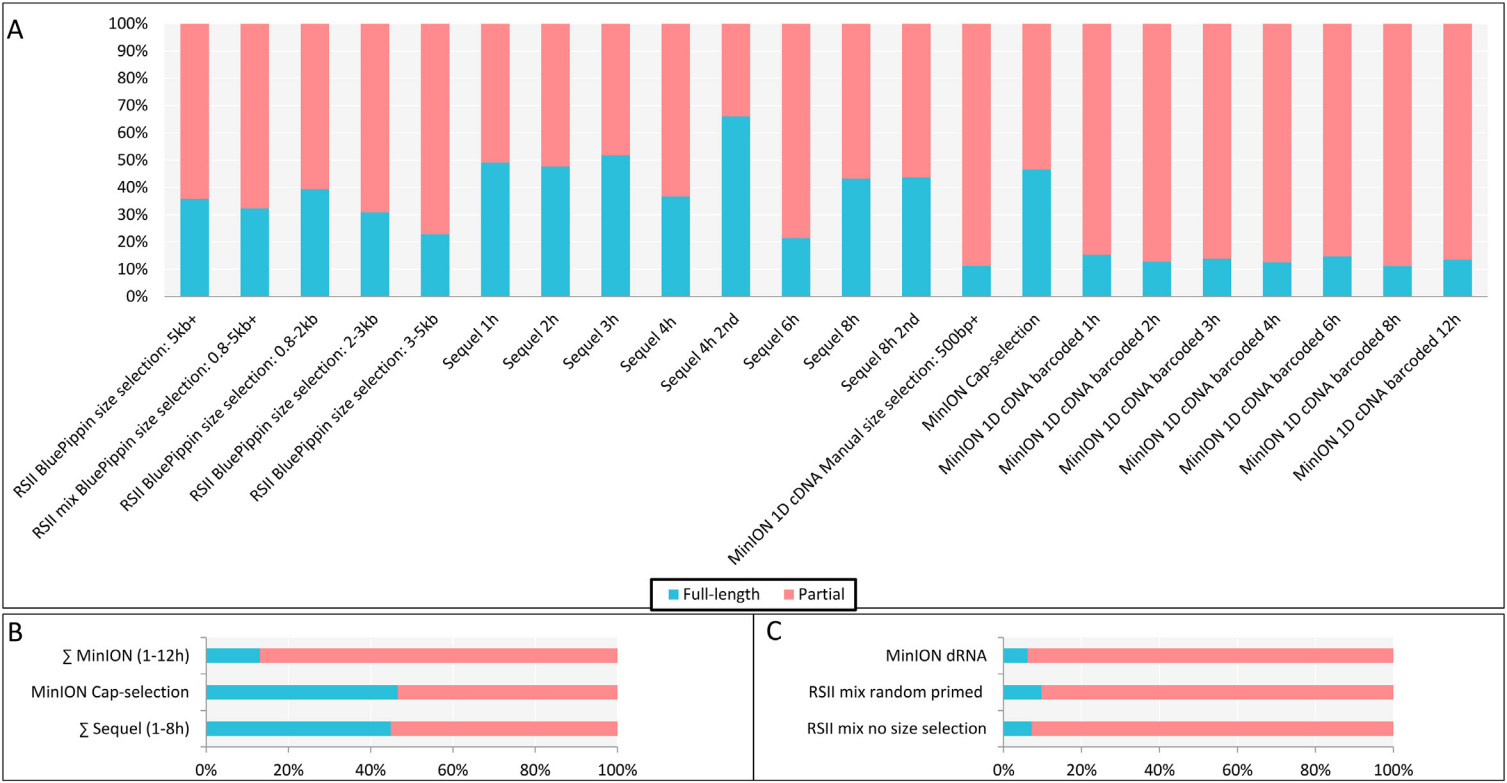
Distribution of sequencing reads. **(A)** The stacked bar chart of the proportion of full-length and partial reads from PolyA cDNA-sequencing shows large differences between the various library-preparation and sequencing methods. All of the PacBio methods and the Cap-selected ONT approach resulted in a higher percentage of full-length reads. The weakest ratios of complete/incomplete reads are from MinION 1D sequencing. The explanation for this result is the lack of size selection. In PacBio sequencing, even in the non-size-selected samples, the short RNA fragments were eliminated by the MagBead loading protocol. **(B)** The horizontal bar graph shows the proportion of full-length/partial reads derived from oligo(d)T-primed, non-size-selected cDNA sequencing, generated by the three different library preparation kits utilized in this study (the same cDNA kits were used for PacBio RSII and Sequel libraries). The sum of the read counts was taken from individual time points of Sequel and MinION 1D sequencing. In order to obtain a full set of transcripts, we mixed RNA samples obtained from various time points for the Cap-Seq analysis. No significant difference between the Sequel and the Cap-selected MinION libraries can be observed, while the MinION 1D-Seq produced much fewer complete sequencing reads. **(C)** This figure shows the methods that generated a very low amount of complete reads (<10%). The weak result of the non-size-selected RSII is not to be considered significant because of the very low yield of this run. However, due to technical reasons, this phenomenon is to be expected from the dRNA-seq and from the random primed sequencing.

The PacBio Sequel and the ONT Cap-Seq methods yielded the highest number of full-length reads in our experiments (Fig. 1). The ratio between the complete and partial reads varies within the size-selected RSII samples. ONT 1D cDNA sequencing yielded the lowest ratio of full-length transcripts but the highest number of read counts; therefore, full-length transcripts are also present in a large number in these samples. Even if a large proportion of the reads are incomplete, they can be utilized to, e.g., distinguish between the various transcript isoforms or to identify embedded transcripts, which is essential for the correct kinetic classification [13]. A large number of incomplete reads have been obtained from the dRNA-seq, which were consistent with our previous results [24]. The current method of this technique produces sequencing reads missing varying size short sequences from both ends. Random-primed reverse transcription (RT) - based sequencing rarely gains complete reads; the reason for which is that the primers seldom bind to exactly the 3′-ends of the transcripts. However, these samples provide further significant value to the dataset. For example, random-primed sequencing may result in novel, non-polyadenylated transcripts [25, 26], while direct RNA sequencing data may provide epitranscriptomic information by detecting base modifications (e.g., m7G). Furthermore, the dRNA-seq method is free of artifacts produced by RT and polymeraase chain reaction (PCR) in cDNA sequencing.

The present report provides the first long-read, dynamic RNA profiling dataset from the family of Poxviruses and the host cell line (CV-1), which can redefine the VACV transcriptomic landscape. This study is a very large cohort of data from the currently available third-generation sequencing methods representing the forefront techniques for transcriptome research. As such, the data presented herein can prove to be useful not only at the molecular level and not just for virologists but also with respect to general genomics and bioinformatics.

## Methods

**Figure 2:**
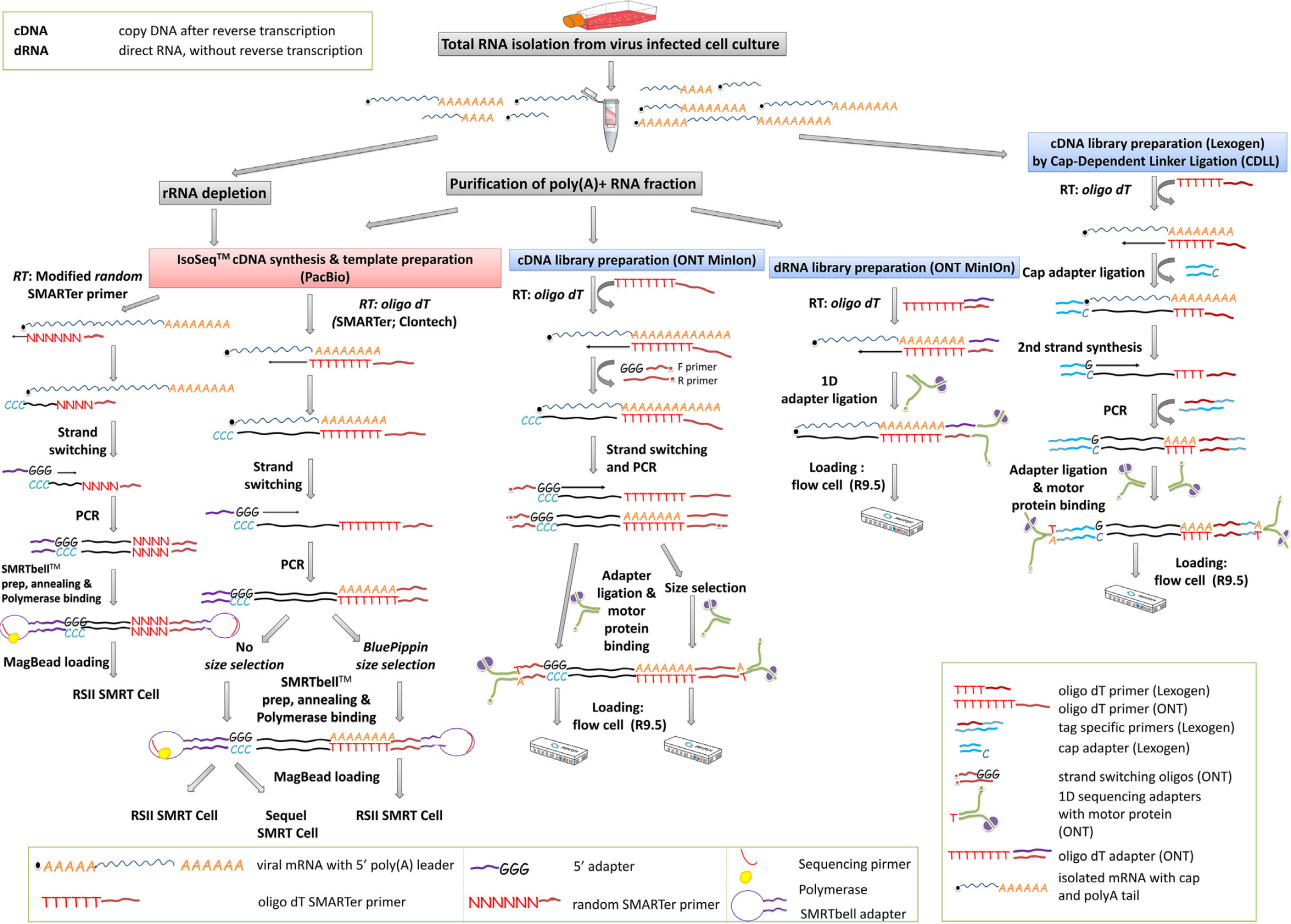
Comprehensive experimental workflow of the PacBio and MinION sequencing.

A detailed workflow pertaining to the different library preparation strategies is presented in Fig. 2 and Table 1.

**Table 1:** Summary table of the different wet lab approaches applied in this study.

Run No.	Platform	Sample collection strategy	Time points (h)	RNA sample	RT priming	Cap-selection	PCR	Size selection	Library prep	Barcodes	Base calling
1	RSII	Static	1, 2, 4, 8	PolyA(+)	Oligo(d)T	No	Yes	No	Iso-Seq	No	SMRT Analysis v2.3.0
2	RSII	Static	1, 2, 4, 8	rRNA depletion	Random hexamer	No	Yes	No	Iso-Seq	No	SMRT Analysis v2.3.0
3	RSII	Static	1, 2, 4, 8	PolyA(+)	Oligo(d)T	No	Yes	BluePippin 0.8kb-5kb+	Iso-Seq	No	SMRT Analysis v2.3.0
4	RSII	Static	1, 2, 4, 8	PolyA(+)	Oligo(d)T	No	Yes	BluePippin 0.8–2kb	Iso-Seq	No	SMRT Analysis v2.3.0
5	RSII	Static	1, 2, 4, 8	PolyA(+)	Oligo(d)T	No	Yes	BluePippin 2-3kb	Iso-Seq	No	SMRT Analysis v2.3.0
6	RSII	Static	1, 2, 4, 8	PolyA(+)	Oligo(d)T	No	Yes	BluePippin 3-5kb	Iso-Seq	No	SMRT Analysis v2.3.0
7	RSII	Static	1, 2, 4, 8	PolyA(+)	Oligo(d)T	No	Yes	BluePippin 5kb+	Iso-Seq	No	SMRT Analysis v2.3.0
8	Sequel	Dynamic	1	PolyA(+)	Oligo(d)T	No	Yes	No	Iso-Seq	No	SMRT Link v5.0.1.9585
9	Sequel	Dynamic	2	PolyA(+)	Oligo(d)T	No	Yes	No	Iso-Seq	No	SMRT Link v5.0.1.9585
10	Sequel	Dynamic	3	PolyA(+)	Oligo(d)T	No	Yes	No	Iso-Seq	No	SMRT Link v5.0.1.9585
11	Sequel	Dynamic	4	PolyA(+)	Oligo(d)T	No	Yes	No	Iso-Seq	No	SMRT Link v5.0.1.9585
12	Sequel	Dynamic	4	PolyA(+)	Oligo(d)T	No	Yes	No	Iso-Seq	No	SMRT Link v5.0.1.9585
13	Sequel	Dynamic	6	PolyA(+)	Oligo(d)T	No	Yes	No	Iso-Seq	No	SMRT Link v5.0.1.9585
14	Sequel	Dynamic	8	PolyA(+)	Oligo(d)T	No	Yes	No	Iso-Seq	No	SMRT Link v5.0.1.9585
15	Sequel	Dynamic	8	PolyA(+)	Oligo(d)T	No	Yes	No	Iso-Seq	No	SMRT Link v5.0.1.9585
16	MinION	Static	1, 2, 3, 4, 6, 8, 12, 16	PolyA(+)	Oligo(d)T	No	Yes	Manual Gel 500bp+	1D cDNA	No	Albacore v.2.0.1
17	MinION	Static	1, 2, 3, 4, 6, 8, 12, 16	Total RNA	Oligo(d)T	Yes	Yes	No	Teloprime&1D	No	Albacore v.2.0.1
18	MinION	Static	1, 2, 3, 4, 6, 8, 12, 16	PolyA(+)	Oligo(d)T	No	No	No	dRNA	No	Albacore v.2.0.1
19	MinION	Dynamic	1	PolyA(+)	Oligo(d)T	No	Yes	No	1D cDNA	Yes	Albacore v.2.0.1
20	MinION	Dynamic	2	PolyA(+)	Oligo(d)T	No	Yes	No	1D cDNA	Yes	Albacore v.2.0.1
21	MinION	Dynamic	3	PolyA(+)	Oligo(d)T	No	Yes	No	1D cDNA	Yes	Albacore v.2.0.1
22	MinION	Dynamic	4	PolyA(+)	Oligo(d)T	No	Yes	No	1D cDNA	Yes	Albacore v.2.0.1
23	MinION	Dynamic	6	PolyA(+)	Oligo(d)T	No	Yes	No	1D cDNA	Yes	Albacore v.2.0.1
24	MinION	Dynamic	8	PolyA(+)	Oligo(d)T	No	Yes	No	1D cDNA	Yes	Albacore v.2.0.1
25	MinION	Dynamic	12	PolyA(+)	Oligo(d)T	No	Yes	No	1D cDNA	Yes	Albacore v.2.0.1

The dynamic transcriptome includes transcripts from various stages of viral infection (from 1 hour to 8 hours for Sequel and from 1 hour to 12 hours for MinION sequencing), while static transcriptome contain transcripts expressed at various time points of infection.

### Cells and viruses

African green monkey (*Chlorocebus sabaeus*) kidney fibroblast cells (CV-1; American Type Culture Collection, [RRID:CVCL_0229]) were cultured in RPMI 1640 medium (Sigma-Aldrich) supplemented with 10% fetal bovine serum (FBS) and antibiotic-antimycotic solution (Sigma-Aldrich) in a 150 cm^2^ culture flask at 37°C in a humidified 5% CO_2_ atmosphere until confluence was reached. The cells (∼2.6 × 10^7^) were washed with serum-free medium before the infection. The highly virulent Western Reserve VACV strain was used in this study. The virus stock was diluted in serum-free RPMI 1640 medium and then used (3 mL virus solution; 10 MOI/cell) for the CV-1 infection. Samples were incubated at 37°C in 5% CO_2_ atmosphere for 1 hour with brief agitation at 10-minute intervals to redistribute the virus. Three milliliters of complete growth medium (RPMI 1640 + 10% FBS) was added to the tissue culture flask, and the infected cells were further incubated for 1, 2, 4, and 8 hours for RSII sequencing; 1, 2, 3, 4, 6, and 8 hours for Sequel; or 1, 2, 3, 4, 6, 8, 12, and 16 hours for MinION sequencing (Table 1) at 37°C in a humidified 5% CO_2_ atmosphere. After the incubation, the cells were rinsed with serum-free RPMI 1640 medium, which was followed by the application of three freeze-thaw cycles. Cells were scraped into 2 mL of phosphate-buffered saline and stored at −80°C until use.

### RNA

Total RNA was purified from the infected cells at various stages of viral infection from 1 to 16 hours post-infection (pi) using the NucleoSpin RNA Kit from Macherey-Nagel. Polyadenylated RNAs were purified from the total RNA using the Oligotex mRNA Mini Kit (Qiagen, Additional File 1). For the analysis of non-polyadenylated RNAs, ribodepletion (Epicentre Ribo-Zero Magnetic Kit H/M/R) was carried out on the total RNA samples. RNAs were quantified (Table 2, row A) by Qubit 2.0 using the Qubit RNA BR Assay Kit for the total RNAs and the Qubit RNA HS Assay Kit for the polyA(+) RNAs (Life Technologies). The quality of the samples was assessed with an Agilent 2100 Bioanalyzer. The samples used had RNA integrity numbers greater than 9.5.

**Table 2:** Summary table of the amount of RNA, cDNA, and library samples used for PacBio Sequel sequencing.

Run No.	Time points (h)	Amount of PolyA(+) RNA used for cDNA preparation (ng)	Concentration of PCR products (ng/μL)	Concentration of SMRTbell libraries (ng/μL)
1	mixed	16.2	86.1	5.8
2	mixed	10.0^a^	109.1	5.2
3	mixed	14.6	75.5	6.9
4	mixed	14.6	98.2	7.2
5	mixed	14.6	84.1	7.1
6	mixed	14.6	89.4	9.1
7	mixed	14.6	77.9	7.9
8	1	27.3	410.0	8.1
9	2	10.0	112.0	9.0
10	3	18.9	87.8	11.1
11	4	51.8	460.0	12.1
12	4	19.9	98.3	6.8
13	6	20.3	95.0	7.8
14	8	39.2	460.0	12.0
15	8	19.6	120.0	6.1

a Amount of rRNA-depleted RNA (for random-primed sequencing)

### Library preparation for PacBio RSII and Sequel sequencing

The cDNAs were generated from the polyA(+) RNA fractions in accordance with PacBio's recommendations for Iso-Seq method using the Clontech SMARTer PCR cDNA Synthesis Kit and No Size Selection’ or the “BluePippin size-selection” protocol (Fig. 2, Table 1). The samples collected at various time points (1, 4, 8, and 12 hours pi) were mixed together for the RSII sequencing; however, different time points (1, 2, 3, 4, 6, and 8 hours pi) were used individually for the production of cDNA libraries for the Sequel method. An rRNA-depleted sample mixture (1, 4, 8, and 12 hours) was converted to cDNA with modified random hexamer primers (Table 3) instead of the SMARTer Kit's oligo(d)T-containing oligo. The amounts of the PCR products were measured by Qubit (Table 2, row B). The detailed library preparation methods are described in our recent publication [23]. Briefly, SMRTbell Template Prep Kit 1.0 was used for SMRTbell library production (the libraries were quantified by Qubit [Table 2, row C]), followed by primer annealing using the DNA Sequencing Reagent Kit 4.0 v2 and polymerase (DNA Polymerase P6) binding for RSII sequencing, whereas the Sequel Sequencing Kit 2.1 and Sequel DNA Polymerase 2.0 were applied for the Sequel platform. Samples were bound to magbeads (MagBead Kit v2) for loading onto the PacBio instruments. The RSII movie lengths were set for 240 minutes, while 600-minute movies were captured using the Sequel technique. A single movie was recorded for each SMRT Cell. Seventeen RSII SMRT Cells v3 and 8 Sequel SMRT Cells v2 (SMRT Cell 1M) were used for sequencing. The cDNA samples and the SMRTbell templates were quantified (Table 2) by Qubit using Qubit dsDNA HS (High Sensitivity) Assay Kit.

**Table 3:** Primers sequences used in this study for the reverse transcription reactions.

Sequencing method	Library prep step	Name, availability	Catalog No.	Sequence (5' → 3')
PacBio amplified PolyA	RT	3' SMART CDS primer II A -SMARTer PCR cDNA Synthesis kit (Clontech)	634 925 & 634 926	AAGCAGTGGTATCAACGCAGAGTAC(T)_30_VN
PacBio amplified Random	RT	Custom-made (IDT DNA)	–	AAGCAGTGGTATCAACGCAGAGTACNNNNNN (G: 37%; C: 37%; A: 13%; T: 13%)
MinION cDNA	RT	Poly(T)-containing anchored primer [(VN)T20 - ONT recommended, custom-made (Bio Basic)	–	5phos/ACTTGCCTGTCGCTCTATCTTC(T)_20_VN
MinION CAP-Seq	RT	TeloPrime Full-Length cDNA Amplification Kit (Lexogen)	013.08 & 013.24	TCTCAGGCGTTTTTTTTTTTTTTTTTT
MinION dRNA	RT	RT adapter—Direct RNA Sequencing Kit (Oxford Nanopore Technologies)	SQK-RNA001	GAGGCGAGCGGTCAATTTTCCTAAGAGCAAGAAGAAGCCTTTTTTTTTT
MinION CAP-Seq	test qPCR	D1R fw—custom-made (IDT DNA)	–	CGAACTAGAGGACCGTTGGG
MinION CAP-Seq	test qPCR	D1R rev—custom-made (IDT DNA)	–	TTTCCAGGTCAGCACCGTTT
MinION cDNA barcoded	barcoding	A1/>BC01/(ONT PCR Barcoding Kit 96)	EXP-PBC096	AAGAAAGTTGTCGGTGTCTTTGTG
MinION cDNA barcoded	barcoding	A2/>BC02/(ONT PCR Barcoding Kit 96)	EXP-PBC096	TCGATTCCGTTTGTAGTCGTCTGT
MinION cDNA barcoded	barcoding	A3/>BC03/(ONT PCR Barcoding Kit 96)	EXP-PBC096	GAGTCTTGTGTCCCAGTTACCAGG
MinION cDNA barcoded	barcoding	A4/>BC04/(ONT PCR Barcoding Kit 96)	EXP-PBC096	TTCGGATTCTATCGTGTTTCCCTA
MinION cDNA barcoded	barcoding	A5/>BC05/(ONT PCR Barcoding Kit 96)	EXP-PBC096	CTTGTCCAGGGTTTGTGTAACCTT
MinION cDNA barcoded	barcoding	A6/>BC06/(ONT PCR Barcoding Kit 96)	EXP-PBC096	TTCTCGCAAAGGCAGAAAGTAGTC
MinION cDNA barcoded	barcoding	A7/>BC07/(ONT PCR Barcoding Kit 96)	EXP-PBC096	GTGTTACCGTGGGAATGAATCCTT
PacBio	adapter ligation	PacBio blunt adapter (PacBio Template Prep Kit 1.0)	PN 100–222-300	ATCTCTCTCTTTTCCTCCTCCT-CCGTTGTTGTTGTTGAGAGAGAT
MinION	adapter ligation	5' adapter (ONT Ligation Sequencing 1D kit)	SQK-LSK108	GGTGCTG
MinION	adapter ligation	3' adapter (ONT Ligation Sequencing 1D kit)	SQK-LSK108	TTAACCT

The table also contains the sequence of the gene-specific primer pair used for the amplification of D1R gene of VACV, as well as the sequencing adapters and barcodes.

### ONT MinION cDNA sequencing

The polyA(+) RNAs were used for cDNA sequencing on the MinION device. We prepared one library from the RNA mixture (RNA samples from the 1, 2, 3, 4, 6, 8, 12, and 16 hours pi); but the various time points were also sequenced individually (Table 4). For the library preparation, we used the ONT 1D strand-switching cDNA by ligation protocol (Version: SSE_9011_v108_revS_18Oct2016), the Ligation Sequencing 1D kit (SQK-LSK108, Oxford Nanopore Technologies), and the NEBNext End repair/dA-tailing Module NEB Blunt/TA Ligase Master Mix (New England Biolabs), according to the manufacturers’ recommendations. Briefly, 50 ng of the polyA(+)-selected RNA samples were subjected to RT using Poly(T)-containing anchored oligonucleotides [(VN)T20; ordered from Bio Basic, Canada], (Table 3), dNTPs (10 mM, Thermo Scientific), Superscript IV Reverse Transcriptase Kit (Life Technologies), RNase OUT (Life Technologies), and strand-switching oligonucleotides with three O-methyl-guanine RNA bases (PCR_Sw_mod_3G; ordered from Bio Basic, Canada). First-strand cDNAs were generated at 50°C for 10  minutes of incubation, which was followed by the strand-switching step at 42°C for 10  minutes and a final inactivation step at 80°C for 10  minutes. Double-stranded cDNAs (5 μL from each) were amplified by using KAPA HiFi DNA Polymerase (Kapa Biosystems), Ligation Sequencing Kit Primer Mix (supplied by the 1D Kit), and a Veriti Thermal Cycler (Applied Biosystems). The initial denaturation was conducted at 95°C for 30 seconds (1 cycle), the denaturation was carried out at 95°C for 15 seconds (15 cycles), the annealing step was set to 62°C for 15 seconds (15 cycles), while the elongation was set to 65°C for 4  minutes (15 cycles). The final extension step was carried out at 65°C for 1  minute. NEBNext End repair/dA-tailing Module (New England Biolabs) and the NEB Blunt/TA Ligase Master Mix (New England Biolabs) were used for end repair and for adapter ligations, respectively. The adapter sequences were provided by the 1D kit. Agencourt AMPure XP magnetic beads (Beckman Coulter) were used to purify the samples following each enzymatic step. The Qubit Fluorometer (Life Technologies Qubit 2.0) and the Qubit (ds)DNA HS Assay Kit were applied to measure the quantity of the libraries. Samples were loaded on R9.4 SpotON Flow Cells, and base calling was performed using Albacore v1.2.6. The PCR amplicons derived from the mixed RNA sample were size-selected manually and then run on Ultrapure Agarose gel (Thermo Fischer Scientific), followed by the isolation of 500 bp+ fragments using the Zymoclean Large Fragment DNA Recovery Kit (Zymo Research). The individually sequenced samples were labeled with barcodes applying a combination of two ONT protocols: first, the 1D protocol was used, but after the first end-prep step, we switched to the 1D PCR barcoding (96) genomic DNA (SQK-LSK108) protocol (version: PBGE96_9015_v108_revS_18Oct2016, updated 25/10/2017), which was then followed by the barcode ligation step using the ONT PCR Barcoding Kit 96 (EXP-PBC096): the barcode adapters (Table 3) were ligated to the end-prepped cDNA samples using the NEB Blunt/TA Ligase Master Mix (New England Biolabs), then they were amplified by PCR with Kapa HiFi DNA Polymerase. The quantities of the libraries were measured by Qubit 2.0 (Table 4).

**Table 4:** Summary of the amount of RNA, cDNA, and library samples used for ONT MinION sequencing.

Library	Starting RNA	Starting RNA amount (ng)	cDNA amount (PCR product, ng)	Library used for sequencing (ng)	Barcode no.	Number of flow cells
1D cDNA	polyA(+) mixed	29	253	65	–	1
1D cDNA	polyA(+) mixed	29	251	48	–	
1D cDNA	polyA(+) 1h	50	117	150	A1	1
1D cDNA	polyA(+) 2h	50	387		A2	
1D cDNA	polyA(+) 3h	50	360	300	A3	1
1D cDNA	polyA(+) 4h	50	180		A4	
1D cDNA	polyA(+) 6h	50	207		A5	
1D cDNA	polyA(+) 8h	50	103		A6	
1D cDNA	polyA(+) 12h	50	130		A7	
dRNA	polyA(+) mixed	60	no PCR	10.2	–	1
Cap-Seq	total RNA (1, 2, 3h)	2 μg	240	240	–	1
Cap-Seq	total RNA (4, 6, 8, 12, 16h)	2 μg	1125	320	–	1

### ONT MinION cDNA-sequencing on cap-selected samples

For more precise identification of the 5′-ends of the full-length transcripts, a Cap-selection method was applied and combined with the ONT 1D cDNA library preparation protocol. The cDNAs were generated from a mixed total RNA sample (containing RNAs from 1, 2, 3, 4, 6, 8, 12, and 16 hours pi; Tables 1 and 4) by using the TeloPrime Full-Length cDNA Amplification Kit (Lexogen). The protocol contains a PCR amplification step. The specificity of the products was checked by qPCR (Rotor-Gene Q). A VACV gene-specific primer (D1R gene, Table 3) and ABsolute qPCR SYBR Green Mix (Thermo Fisher Scientific) were used. The amplified PolyA(+)- and Cap-selected samples were subjected to the ONT's 1D strand-switching cDNA by a ligation method (ONT Ligation Sequencing 1D kit); they were end-repaired, then ligated to the 1D adapters (NEBNext End repair/dA-tailing Module NEB Blunt/TA Ligase Master Mix).

### ONT MinION—dRNA sequencing

In order to avoid the potential PCR biases, the amplification-free direct RNA sequencing (DRS) protocol (Version: DRS_9026_v1_revM_15Dec2016) from the ONTs was applied. The library was prepared from a PolyA(+) mixture of 8 time points (1, 2, 3, 4, 6, 8, 12, and 16 hours pi; Table 4). RNA was mixed with the RT (oligo(dT)-containing T10) adapter (provided by the ONT Direct RNA Sequencing Kit; SQK-RNA001) and T4 DNA ligase (2M U/mL; New England BioLabs). Following a 10-minute incubation, the first-strand cDNAs were generated with SuperScript III Reverse Transcriptase (Life Technologies), according to the DRS protocol, at 50°C for 50  minutes, then at 70°C for 10  minutes in a Veriti Thermal Cycler. Samples were purified by using Agencourt AMPure XP Beads (Beckman Coulter). XP Beads were handled before use with RNase OUT (40 U/μL; Life Technologies; 2 U enzyme/1 μL bead). Washed samples were eluted in Ambion Nuclease-Free Water (Thermo Fisher Scientific). An RMX sequencing adapter was ligated to the samples with NEBNext Quick Ligation Reaction Buffer (New England BiceoLabs) T4 DNA ligase. Samples were washed with RNase OUT-treated XP beads and Wash Buffer (part of the DRS Kit). Finally, the samples were eluted in 21 μL Elution Buffer (provided by the DRS Kit). The concentrations of the reverse-transcribed and adapter-ligated RNAs were measured using the Qubit 2.0 Fluorometer and Qubit dsDNA HS Assay Kit (Life Technologies). The ONT cDNA libraries, the Cap-selected samples, and the direct RNA libraries were loaded on 3, 2, and 1 ONT R9.4 SpotON Flow Cells for sequencing, respectively. The runs were carried out using MinKNOW. Voltage levels were set and reset in line with the suppliers’ recommendations.

### Data analysis and visualization

**Figure 3:**
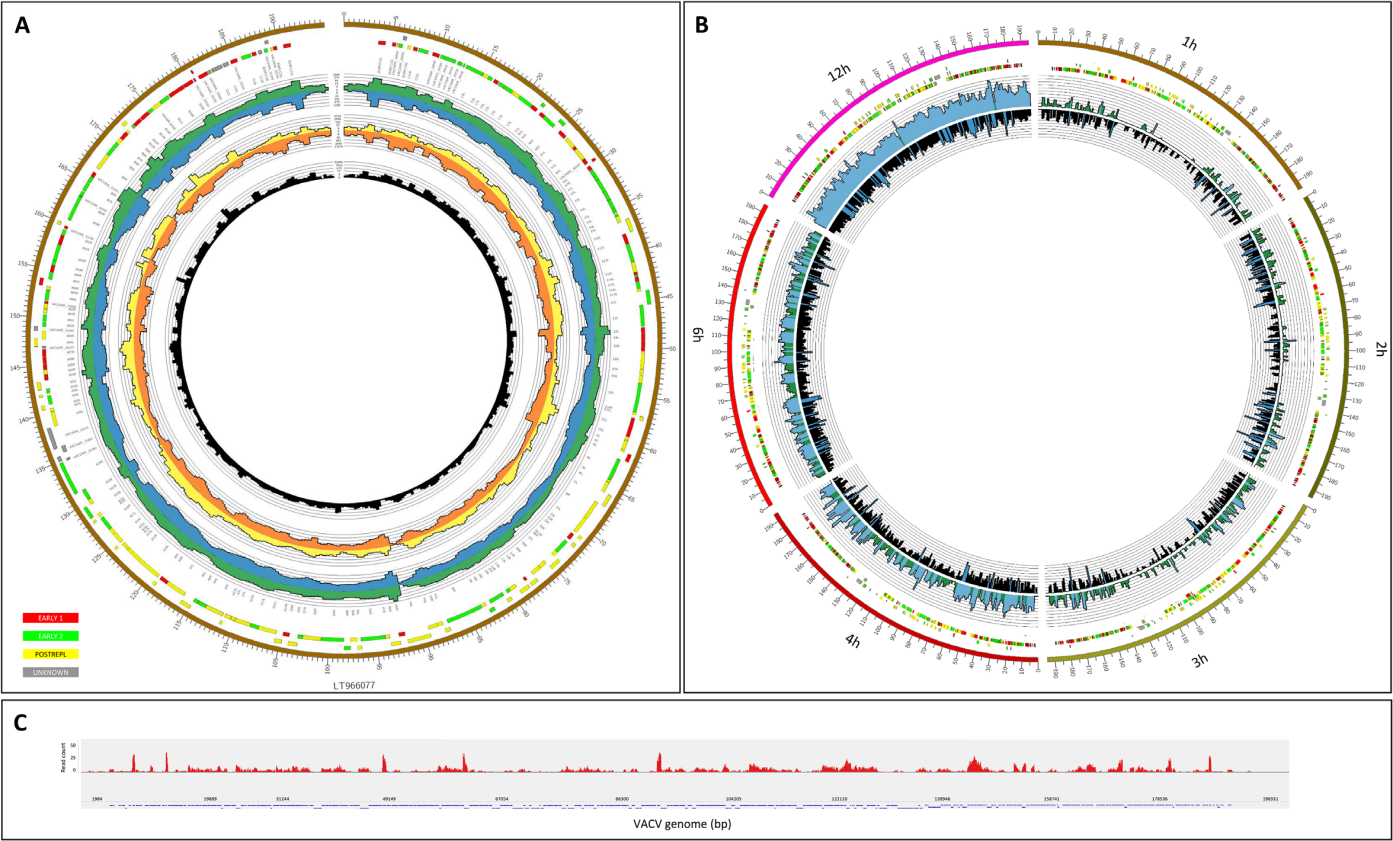
Representation of the depth of viral read coverages generated from different long-read sequencing techniques. **(A)** Circos plot showing the genome-wide transcriptome profile of VACV. The colored boxes represent the genes that belong to different kinetic classes (red: early 1 [early]; green: early 2 [early-late]; yellow: postreplicative [late]; gray: unknown) [13]. Data derived from the five different library preparation and sequencing methods used in this study are shown on the histogram as follows: green: Sequel all data (data from different time points are mixed together); blue: RSII mixed sample; yellow: MinION 1D cDNA mixed sample; orange: MinION Cap-selected mixed sample; black: MinION 1D cDNA barcoded all data (data from different time points are mixed together). **(B)** Visualization of read coverage on VACV genome at individual time points. Six time points that were sequenced by PacBio Sequel (inner radius) and ONT MinION (outer radius) have been visualized in a segmented circos plot (every segment represents an individual time point). **(C)** Sashimi plot presentation of the dRNA-seq data across the VACV genome.

The PacBio RSII reads of insert (ROI) reads were generated using the RS_ReadsOfInsert protocol of the SMRT Analysis v2.3.0, with the following settings: Minimum Full Passes = 1, Minimum Predicted Accuracy = 90, Minimum Length of Reads of Insert = 1, Maximum Length of Reads of Insert = No Limit. These consensus reads were mapped using GMAP (GMAP, RRID:SCR_008992) [27] (version 2017–09-30) with the default settings. GMAP was chosen in this work because we found it to be the best long-read aligner in our earlier publications [18–24]. Others have also found that GMAP produces the best alignment results [e.g., 28]. The ROIs from the Sequel data were created using SMRT Link5.0.1.9585. ONT's Albacore software v.2.0.1 (Albacore, RRID:SCR_015897) was then applied for the MinION base calling. This base caller is able to identify the nucleotide sequences directly from raw sequencing data. The reads were aligned with the GMAP program using the same setting as described above. The raw reads were aligned to the reference genome of the virus (LT966077.1) and the host cell (*Chlorocebus sabaeus*) (GenBank assembly accession: GCA_000 409795.2 [latest]; RefSeq assembly accession: GCF_000 409795.2 [latest]). In-house routines were used to acquire the quality information presented in this data note. The code has been archived on Github [29]. Bedtools genomecov software (BEDTools, RRID:SCR_006646) [30] was used to generate coverage files with the following parameters: -split—ibam. The output bed files from cDNA sequencing were visualized by Circos plot [31] (Fig. 3), while the low-coverage dRNA-seq data was shown using the Integrative Genomics Viewer (IGV, RRID:SCR_011793)[32].

## Data Summary

**Figure 4:**
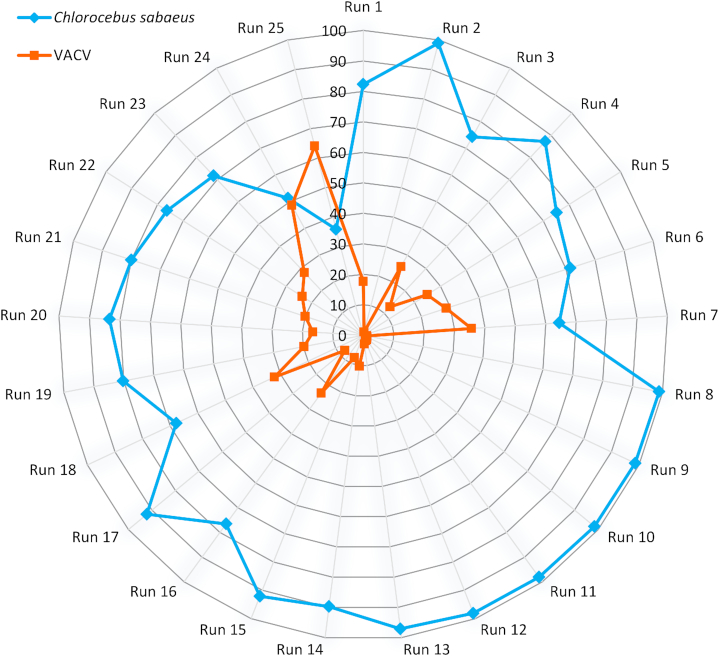
Polar plot representation of the percentages of virus-host read counts.

**Figure 5:**
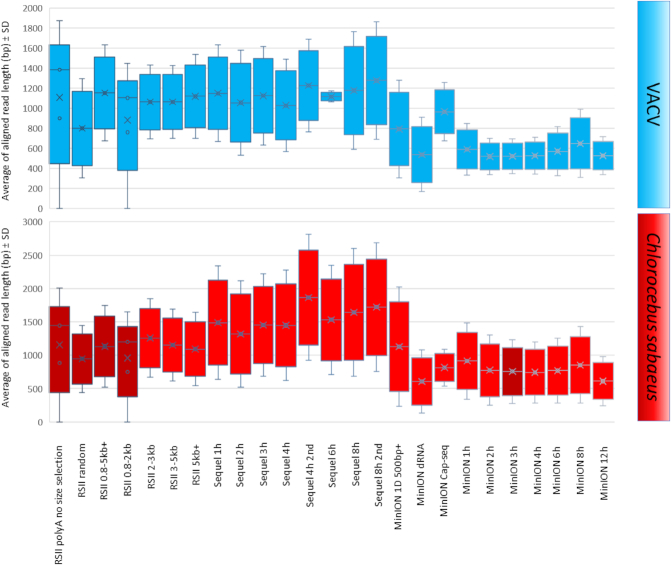
Box plot presentation of the average of aligned read lengths obtained from the applied sequencing methods. The reads were mapped to the VACV and to the host genome, and the average lengths were plotted with the standard deviation values.

The raw sequencing reads were mapped to both the VACV reference genome and to the host genome. In this study, we generated full-length transcripts of VACV and the CV-1 cells, yielding about 3.17 Gb of mapped sequencing data. Sequencing on the RSII and Sequel platforms yielded 86,728 and 850,803 ROIs aligned to the viral and the host genomes, respectively. The utilized nanopore-based cDNA sequencing approaches resulted in 413,497 VACV-specific reads (Table 5, Additional File 2), while we obtained 155,876 reads from the Cap-selected samples. The different MinION sequencing methods yielded 1,590,975 reads that mapped to the host genome. The ratio of viral transcripts is 21.9% on average in our samples. The exact ratio is dependent on the titer of the virus used for the infection, as well as on the stage of the viral life cycle at the examination period. The sequencing method affects the ratio of read counts between the virus and host cell; e.g., the MinION 1D-Seq method yields a higher amount of shorter reads compared to the PacBio Sequel technique. The VACV transcripts are relatively short compared to the host or to other large DNA viruses (such as herpesviruses and baculoviruses), which is assumed to result in the relatively high ratio of viral reads compared to the host reads in the MinION samples (Fig. 4, Additional File 3). The ratio of the viral reads in the RSII samples (with or without size selection) is lower than that of the MinION samples; however, this ratio is significantly higher than in the Sequel samples (without size selection). The Sequel platform generated the same or longer read length than the RSII size-selected samples (Fig. 5, Additional File 2). In contrast to the Sequel and MinION samples where individual time points of viral infection were analyzed, we used mixed time-point samples for the RSII sequencing, therefore the comparison of the obtained results is not possible. The PacBio MagBead loading method and the overall yield of the given runs can also account for the generation of varying ratio of viral reads in the different samples.

**Table 5:** Summary statistics of the sequencing reads that mapped to the viral and the host reference genomes from each run.

		VACV		*C. sabaeus*	
Run no.	Major specificities of libraries	Number of mapped reads^a^	Coverage	Number of mapped reads^a^	Coverage
1	RSII mix no size selection	110	0.7684	512	0.0002
2	RSII mix random primed	31	0.1137	2,905	0.0008
3	RSII mix BluePippin size selection: 0.8–5kb+	23,802	114.55	50,200	0.0167
4	RSII BluePippin size selection: 0.8–2kb	1,283	6.4407	15,206	0.0055
5	RSII BluePippin size selection: 2–3kb	5,029	24.46	8,752	0.0035
6	RSII BluePippin size selection: 3–5kb	20,103	97.639	68,766	0.0217
7	RSII BluePippin size selection: 5kb+	8,848	46.399	16,024	0.0048
8	Sequel 1h	455	1.8081	38,239	0.0145
9	Sequel 2h	527	2.2227	38,255	0.0163
10	Sequel 3h	1,068	4.9862	68,500	0.032
11	Sequel 4h	809	3.0213	42,379	0.018
12	Sequel 4h 2nd	4,522	22.49	233,709	0.109
13	Sequel 6h	3,031	13.401	101,745	0.0499
14	Sequel 8h	5,482	27.619	101,624	0.0548
15	Sequel 8h 2nd	11,628	63.066	63,987	0.0264
16	MinION 1D cDNA Manual size selection: 500bp+	89,778	302.1	293,048	0.0801
17	MinION dRNA	1,259	3.3894	14,757	0.0026
18	MinION Cap-selection	155,876	550.86	327,964	0.059
19	MinION 1D cDNA barcoded 1h	17,048	31.358	69,060	0.0166
20	MinION 1D cDNA barcoded 2h	94,125	147.62	474,008	0.0949
21	MinION 1D cDNA barcoded 3h	22,029	34.865	88,064	0.017
22	MinION 1D cDNA barcoded 4h	41,700	66.981	134,090	0.0249
23	MinION 1D cDNA barcoded 6h	42,082	75.602	106,989	0.0205
24	MinION 1D cDNA barcoded 8h	48,437	101.6	51,071	0.0099
25	MinION 1D cDNA barcoded 12h	57,039	92.483	31,924	0.0028

^a^The difference between the yield of the size-selected and nonsize-selected samples might be caused by the underloading of the SMRT Cell and it is independent from the size-selection step. In some cases, PacBio run results in low output, for which the possible reason is the underloading of the Cells.

**Figure 6:**
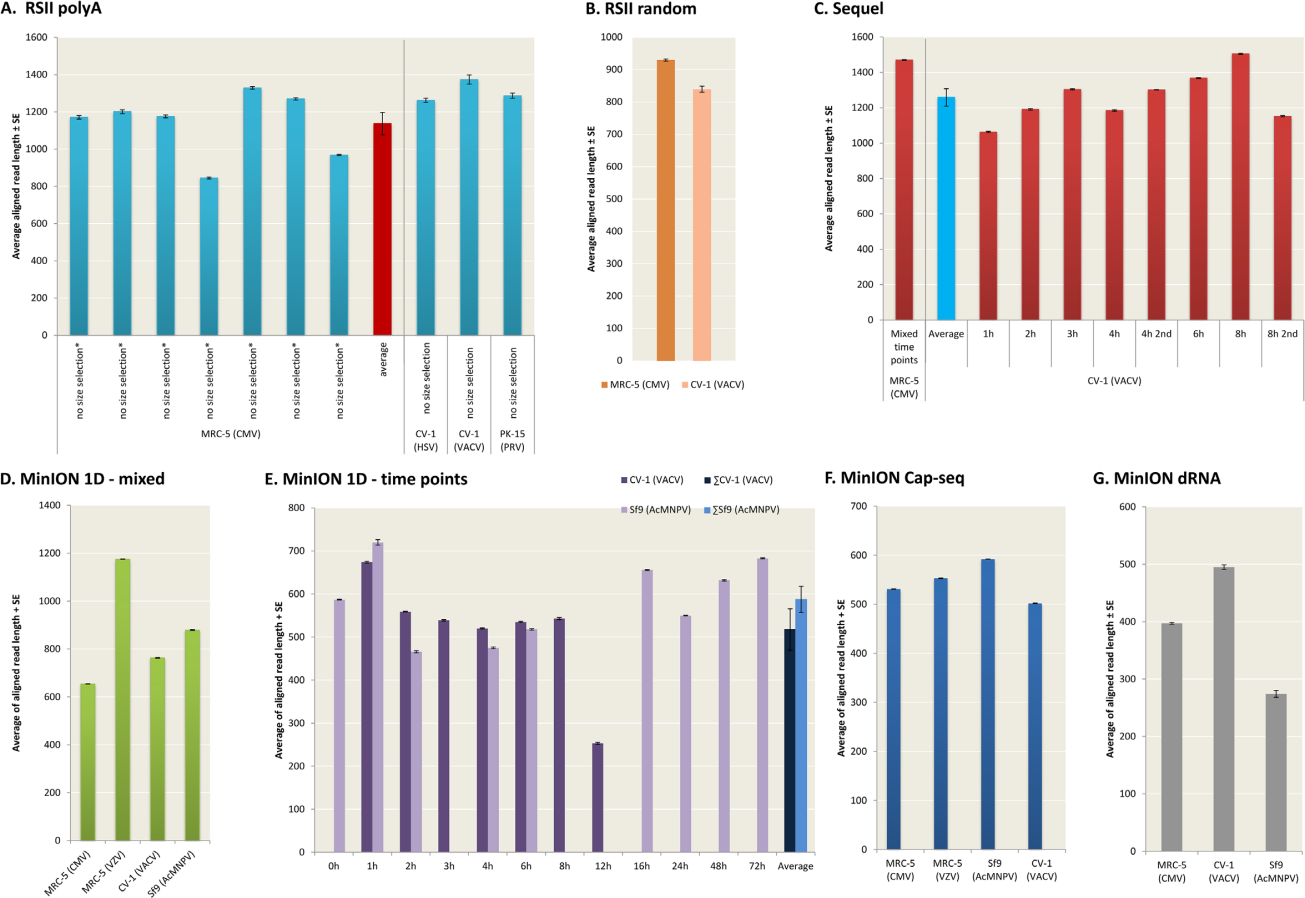
Comparison of the read lengths mapped to the host genome of this and other studies. It must be noted here that the analyzed cell lines are from different organisms and/or they were infected with different viruses using different incubation time points. **(A-C**) RSII and Sequel platforms provide relatively fixed read length (RSII: 800–1,400 bp; Sequel: 1,050–1,500 bp). The average read length of CV-1 samples is longer than those of the MRC-5 in the RSII PolyA-sequencing; however, the opposite result has been obtained with the random-primed RSII and the Sequel PolyA-Seq. (**D-G)** The MinION platform produces greater length variance (250–1,200 bp) except for the Cap-Seq approach, which shows a very small difference between the read lengths among the four different cell lines. Cell lines: African green monkey kidney fibroblast cells (CV-1) infected with VACV or herpes simplex virus type 1 (HSV-1); human lung fibroblast cells (MRC-5) infected with human cytomegalovirus (HCMV) or varicella-zoster virus (VZV); porcine kidney 15 (PK-15) cell line infected with pseudorabies virus (PRV); and Sf9 insect cell line infected with the baculovirus *Autographa californica* multiple nucleopolyhedrovirus (AcMNPV).

The average lengths of ROIs aligning to the VACV genome were 1,098 bp for PacBio RSII and 1,157 bp for the Sequel. The MinION average read lengths were as follows: 557 bp for ONT barcoded cDNA sequencing, 792 bp for the cDNA-Seq, and 965 bp for the Cap-selected samples (Fig. 5, Additional File 2). The average read length produced by dRNA sequencing was 537 bp. It should be noted that the library preparation and size-selection methods resulted in different samples in terms of length; all library preparation methods resulted in longer average read length aligning to the host genome than to the viral genome (Fig. 5, Additional File 2). We have compared the average aligned read-length of cellular transcripts obtained in this and in other studies [18, 19, 21, 24, 33–35] in Fig. 6.

The various sample preparation and sequencing techniques produced different read lengths, read numbers and precision, as well as different artifacts. There is a relatively large difference between the PacBio and ONT sequencing approaches concerning the quality of the sequencing reads; the PacBio technique produces fewer mismatches, insertions and deletions (INDELs) than nanopore sequencing. The various sequencing platforms recommend different cDNA production kits, which contain different enzymes and primers for both the RT and PCR. The various primers and library preparation conditions could produce artifacts; however, these can be easily filtered out if we compare the results of different methods. The PacBio MagBead loading selectively eliminates the short fragments (<1,000 bp). On the one hand, removal of incomplete cDNAs can be advantageous, at the same time, it is unfavorable, as we are unable to detect the shorter transcripts and RNA isoforms. Our data demonstrate that the ONT MinION sequencing resulted in higher error rates for both INDELs and mismatches in comparison to the PacBio systems (Additional File 2). The composition of the errors of the three platforms (RSII, Sequel, and MinION) and the various library preparation techniques (e.g., dRNA-seq, Cap-Seq, etc.) are different. Mismatches are the most common errors in ONT cDNA-Seq, which is consistent with others’ data [36]. In agreement with the previously published data [36], our results also indicate that insertions are the least frequent errors in ONT MinION sequencing. In accordance with others’ results [37], our dRNA reads have higher deletion error rates than either of the cDNA datasets and lower than those of the ONT cDNA-Seq samples, which might be the result of the lower coverage of the dRNA-seq. In contrast to others’ results [36], deletions are the major errors in our PacBio RSII dataset. The quality of the Sequel dataset shows “coverage-specificity”; mismatches are the major errors in the lower-coverage samples, which complies with others’ data [36], while contrary to the same report in that the insertions are more frequent in the higher coverage samples in our dataset. The RSII and the Sequel platforms produce the same error rate. Conversely, our data show a somewhat higher error rate for the Sequel, which might be the result of the different library preparation approaches. In sum, the absolute error rate of both PacBio platforms is low, while the higher ONT error rate is “compensated” by the higher coverage. It is worth mentioning that read quality is not essential for transcriptome analysis if well-annotated genomes are available.

Our transcriptomic survey yielded extremely high coverage across the viral genome (Fig. 3): 290.1 fold for the RSII, 138.6 fold for the Sequel, 550.5 fold for the bar-coded MinION cDNA-Seq, 550.8 fold for the Cap-selected samples, and 302.1 fold for the cDNA sequencing (more detailed information, including quality information, is available in Table 5 and Additional Files 2 and 4). Our data show that the entire VACV genome is transcriptionally active, generating RNAs from both DNA strands. Our dataset also contains 1.56 Gb of raw data from Sequel sequencing, as well as from MinION dRNA-seq.

**Figure 7:**
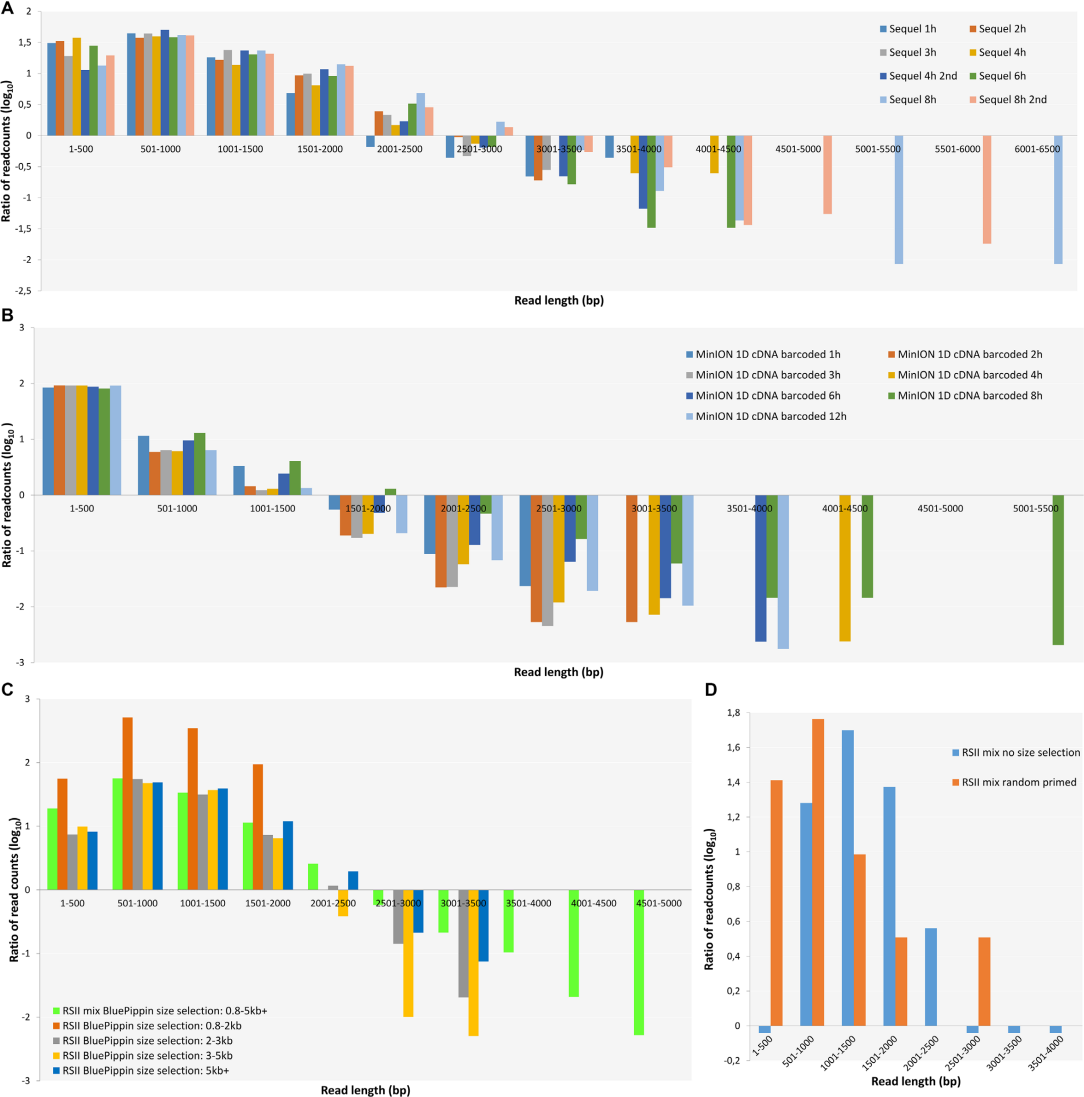
Bar chart representations of read-lengths distributions (depicted for 500 bp long bins, at log10 scale). **(A)***Sequel*. Most of the reads fall within the range of 501–1,000 bp at each time point. There are no substantial length differences between the samples within the first four intervals; however, the earlier time points disappear later; only the samples from 4, 6, 8, and 12 hours contain reads longer than 4,000 bp, while reads longer than 4,500 bp could be detected only within the 8-hour post-infection samples. **(B)***MinION*. Most of the reads fall to the shortest range (1–500 bp), and very few reads are longer than 3,501 bp from the 4-, 6-, 8-, and 12-hour samples. (**C)***RSII size-selected samples*. The shortest and longest reads are overrepresented in the 0.8–5 kb+ sample. The 0.8–2 kb sample represents the shortest read population; no reads are longer than 2 kb. There is no significant difference between the samples at the size ranges 2–3 kb, 3–5 kb, and 5 kb+; the largest amount of transcripts is within the 501–1,000 bp range. The reason for the relatively low read count within the higher size ranges may be that the length of the VACV transcripts are much shorter than, e.g., herpesviruses or baculoviruses. **(D)***RSII no size selected PolyA vs. random-primed samples*.The shorter reads are overrepresented in the random primed sample (< 1,000 bp), while most of the reads from the PolyA-Seq sample fall within the 1,001–2,000 bp interval (this is the typical average read lengths of the PacBio RSII without size selection).

**Figure 8:**
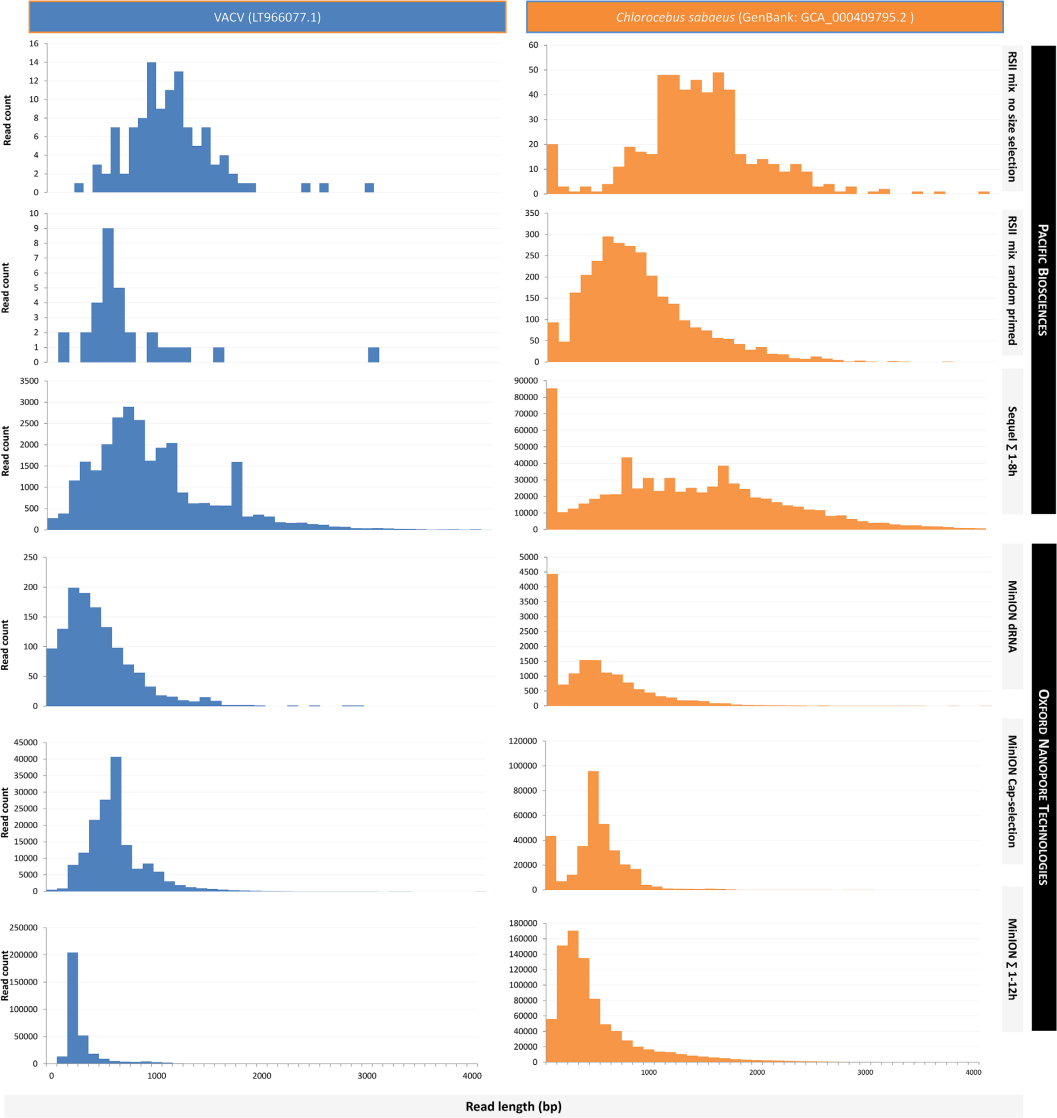
Comparison of the read-length distributions between the VACV and the host (*Chlorocebus sabaeu*s) transcripts within the utilized non-size-selected library preparation methods. Mapped read lengths are expressed in base pairs, and the distribution is shown for 100 bp long bins. The *x-*axis is only presented up to 4,000 base pairs, even though the longest read that was detected was up to as long as 9,000,bp; 99.86% of the alignments fall into this range. In most cases, the PacBio platforms generated longer reads than the ONT methods.

**Figure 9:**
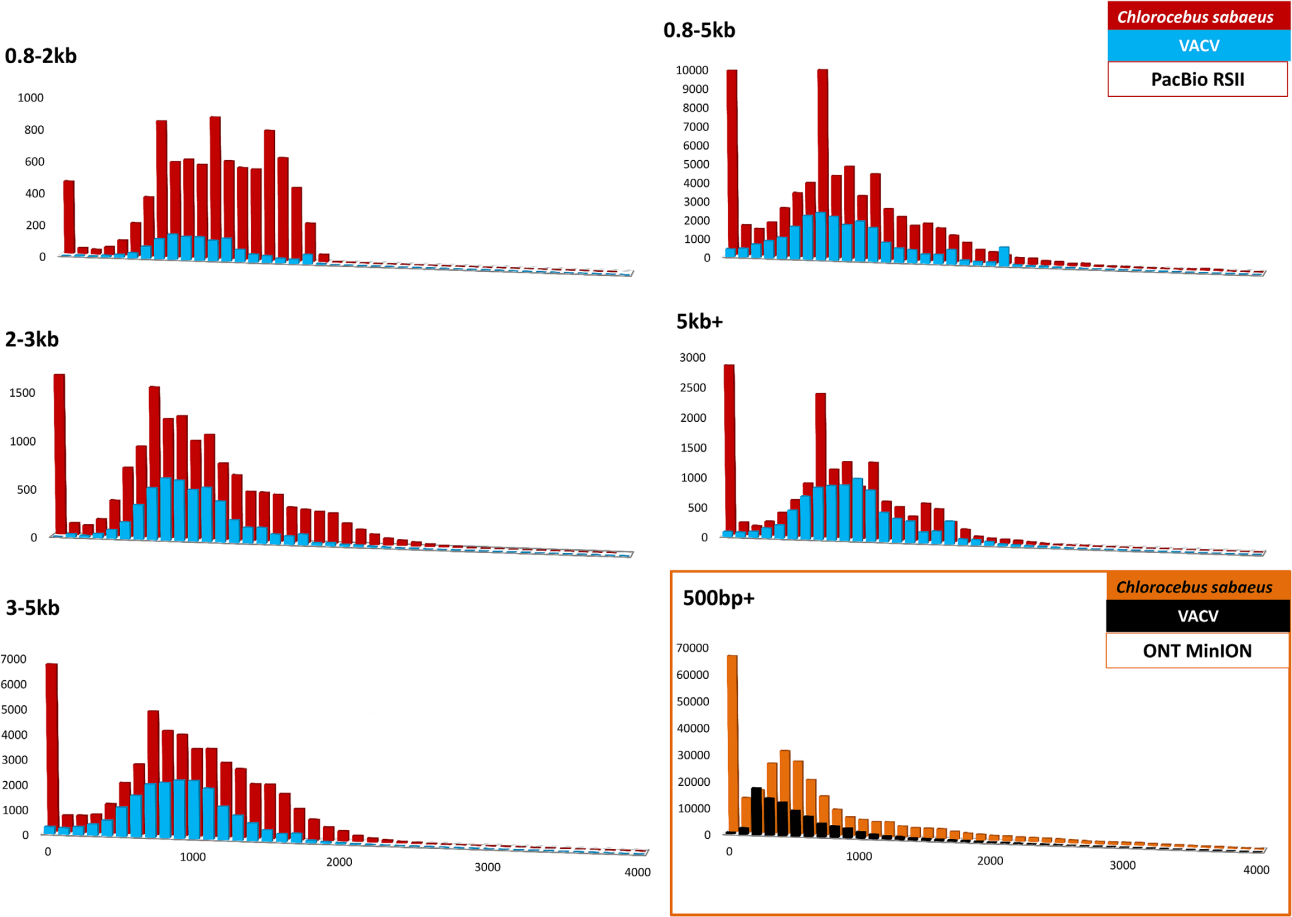
Illustration of the read-length distributions of the VACV and the host (*Chlorocebus sabaeus*) transcripts within the utilized size-selected library preparation methods. Aligned read lengths are shown in base pairs per 100 bp intervals. The distribution patterns of the viral and host cell reads resemble one another in the size-selected RSII samples, especially in the 2–3 kb, 3–5 kb, and 5 kb+ samples. Samples reach their highest peaks around 1,000 bp; however, the peak shifts to the right according to the size selection. There is a significant peak in every sample within the shortest range (1–100 bp) in the host reads. The effect of size selection is the most dramatic in the MinION virus sample; the read counts drastically increase beyond 200 bp.

The read-length distributions for the dataset are shown in Fig. 7 (reads mapped to the VACV genome), as well as in Figs. 8 and 9 (data aligned to the VACV and to the host genome). Detailed information is available in Additional File 5.

The read counts aligned to the mRNAs have been calculated (Additional File 6). Most of the host-specific reads align to the coding region in this dataset (the values vary between 43% and 87% based on the read counts and between 35% and 85% if we compare the number of nucleotides).

**Figure 10:**
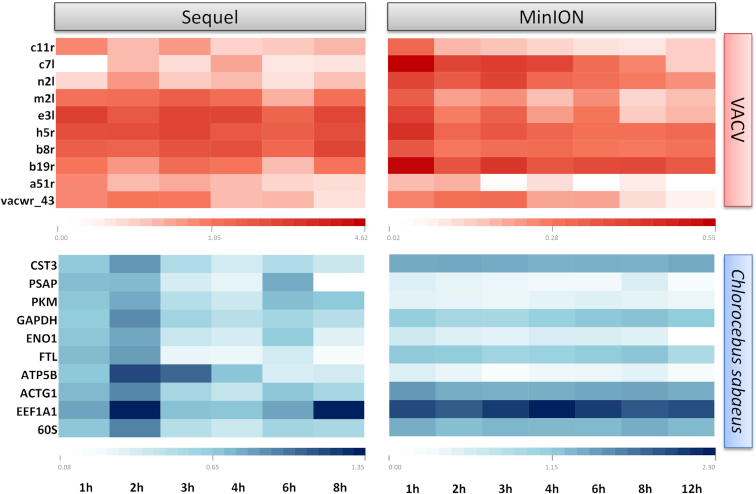
Heat maps depict the relative expression ratios (the proportion of read counts to the total number of reads at a given time point; the values are expressed as percentages [%]). White indicates the lowest relative expression values, while the dark red (VACV) or dark blue (host) represents the highest values. The dynamic profiles of the examined viral genes differ in the two datasets derived from the different sequencing methods. This alteration can be explained by the different read-size preferences of the two methods (however, further data analysis is required for accurate kinetic findings). According to a previous study [13], the examined viral genes belong to the early kinetic class. This is evidenced by the fact that the relative expression values are higher at the early time points, especially in the MinION dataset. The majority of the examined cellular genes show constant expression level (mainly in MinION data), most of them belong to the housekeeping genes: https://hpcwebapps.cit.nih.gov/ESBL/Database/NephronRNAseq/Housekeeping_Genes.html. The expression patterns of the following genes were analyzed: VACV; 1. *c11r*: Epidermal growth factor-like protein (EGF-like protein); 2. *c7l*: Interferon antagonist C7 (host range protein 2); *n2l*: protein N2; m2l: protein M2; *e3l*: protein E3; *h5r*: Late transcription elongation factor H5; *b8r*: Soluble interferon gamma receptor B8; *b19r*: Ankyrin repeat protein B19; *vacwr_4*: Truncated CrmB protein. HOST; *CST3*: *C. sabaeus* cystatin C (XM_007961908.1); *PSAP*: *C. sabaeus* prosaposin, transcript variant X1 (XM_007963126.1); *PKM*: *C. sabaeus* pyruvate kinase PKM (LOC103217002) (XM_007964863.1); *GAPDH*: *C. sabaeus* glyceraldehyde-3-phosphate dehydrogenase (XM_007967342.1); *ENO1*: *C. sabaeus* enolase 1, (alpha), (XM_007980661.1); *FTL*: *C. sabaeus* ferritin, light polypeptide (XM_007997480.1); *ATP5B*: *C. sabaeus* ATP synthase, H+ transporting, mitochondrial F1 complex, beta polypeptide (XM_008003700.1); *ACTG1*: *C. sabaeus* actin, gamma 1 (XM_008013242.1); *EEf1A1*: *C. sabaeus* eukaryotic translation elongation factor 1 alpha 1 transcript variant X1 (XM_008013483.1); *60S*: *C. sabaeus* 60S ribosomal protein L3-like (LOC103247496), mRNA (XM_008019639.1)].

We mapped the raw data to the VACV and to the host mRNAs. Ten viral and 10 host genes that are expressed at every examined time point were chosen for a heat map analysis (Fig. 10). Only the full-length transcripts were calculated for the analysis. A read was considered full length if it contained the 5′ and 3′ adapter sequences as well as the polyA-tail preceding the 3′ adapter. Porechop software v.0.2.3 [38] was used to identify the 5′ and 3′ adapters. Reads lacking an adapter on either the 3′- or the 5′-end, or on both ends, and reads with 5′ or 3′ adapters on both ends were considered as non-full length reads. Reads that were categorized as full length were mapped to the reference sequences by GMAP (Additional File 7). A plus/minus 20 bp range was set to and from the previously annotated transcription start and end sites, and the reads that belonged to this category were used for the analysis. The relative expression ratios of each examined transcript were calculated by dividing the obtained full-length read count of the transcript by the total read count in the given sample.

## Conclusions and Reuse Potential

The present study generated data using state-of-art sequencing technologies (PacBio RSII and Sequel, as well as the ONT MinION platforms, applying a new protocol for barcoding the samples). These data allow a time-course look at the full-length transcriptome of VACV, as well as the CV-1 host cell line.

The dataset was primarily produced for the dynamic characterization of the VACV transcriptome. Another aim was to generate a deep coverage long-read dataset for the analysis of the different transcript isoforms, including length (5′-ends and 3′-ends) variants; mono-, bi-, and polycistronic transcripts; and also to define full-length transcripts produced by the various viral genes. This dataset is useful in understanding the complexity of the genetic regulation of VACV. The provided dataset can also be used to investigate the effect of the viral infection on the gene expression of the host.

The provided binary alignment files contain reads already aligned to the VACV and to the host genome. These aligned reads can be further analyzed by comparing them to the results of various long-read aligners (e.g., BLASR [39], NGMLR [40], Minimap2 [41]) and bioinformatics tools (e.g., samtools [42] or bedtools [43]). Other long-read sequencing programs or pipelines (e.g., SQANTI [44]) can be tested using this dataset.

These data can be visualized by using different programs such as the Geneious [45], Artemis [46], or IGV [32]. Data can be useful for testing novel bioinformatics pipelines or to improve those already available. The files contain terminal polyA sequences as well as the 5′ and 3′ adapter sequences, which can be used to determine the orientations of the reads. The dataset contains the raw dataset from dRNA sequencing (fast5.tar.gz), which can be further analyzed by using the Tombo software package [47], which enables the detection and visualization of modified nucleotides, such as the 6-methyladenine, the most common internal mRNA modification described in eukaryotes [48–50] as well as in viruses [51–53], or the 5-methylcytosine, which is another abundant modification recently confirmed in mRNA [54–57]. To the best of our knowledge, these modifications have not yet been shown in the *Poxviridae* family. The raw data provided from PacBio Sequel sequencing can be used to improve existing base caller algorithms or potentially to develop novel algorithms. Furthermore, the data contain the full set of quality values and kinetic measurements.

This dataset can be used to identify novel VACV and CV-1 transcripts and RNA isoforms including splice variants of the host transcripts, TSS and TES variants, as well as polycistronic transcripts of the virus and the host in order to examine the effect of VACV infection on the host gene expression at the different stages of viral life cycle, as well as for the comparison of the quality and length of the sequencing reads derived from different sequencing platforms. The various library preparation methods can also be compared with one another. The data provided could be used to better understanding the logic of gene expression control of Poxviruses and can also be used to design gene expression vectors.

## Availability of source code and requirements

Project home page: https://github.com/Szunyike/SAM-Statistic-2018

Operating system: Windows

Programming language: VB.NET

Other requirements: NET framework

License: GPL v3

## Availability of supporting data

All of the presented here data were deposited in the European Nucleotide Archive under the accession number PRJEB26434 (Characterization of the Vaccinia virus transcriptome) and PRJEB26430 (Dynamic characterization of the Vaccinia virus transcriptome). Alignments and other data are also available from the *GigaScience* GigaDB repository [58] (**Additional file 8**).

## Additional files

**Additional file 1:** Summary table of the reagents and chemistries used for the sequencing

**Additional file 2:** Summary table of the virus-host ratios within the single runs

**Additional file 3:** Summary statistics of the sequencing reads which mapped to the viral genome (A) and to the host reference genome (B) from each run. SE: standard error. * The difference between the yield of the size-selected and non-size-selected samples might be caused by the underloading of the SMRT Cell and it is independent from the size-selection step. In some cases, PacBio run results in low output, for which the possible reason is the underloading of the Cells.

**Additional file 4:** Summary statistics of the viral and host reads from each run.

**Additional file 5:** Read-length distribution is depicted for 100bp long intervals.

**Additional file 6:** Statistics of the read counts mapped to the host genome versus the host mRNAs.

**Additional file 7:** GenBank accession numbers and URLs of the VACV and host genes selected for heatmap expression analysis.

**Additional file 8:** Correspondence between the file names of alignments deposited in ENA and the names that are used in this manuscript. The table also contains the ENA accession numbers of the study, of the experiments, samples, as well as the runs.

## Abbreviations

m5C: 5-methyl cytosine; m6A: 6-methyl adenine; CV-1: African green monkey (*Chlorocebus sabaeus*) kidney fibroblast cells; ATCC: American Type Culture Collection; CAGE: cap analysis of gene expression; Cap-Seq: Cap-selection; dRNA: direct RNA; E: early; FBS: fetal bovine serum; I: intermediate; IE: immediate-early; INDEL: insertions and deletions; Iso-Seq: Isoform sequencing; L: late; LRS: long-read sequencing; ONT: Oxford Nanopore Technologies; ORF: open reading frame; PacBio: Pacific Biosciences; pi: post-infection; RNA-seq: RNA sequencing; ROI: RPMI 1640: 1640 Roswell Park Memorial Institute medium; RSII: Real-Time Sequencer II; SRS: short-read sequencing; TES: transcription end site; TSS: transcription start site; VACV: vaccinia virus;

## Competing interests

The authors declare that they have no competing interests.

## Funding

This study was supported by the National Research, Development and Innovation Office (NKFIH OTKA K 128 247) and by the Swiss-Hungarian Cooperation Programme (SH/7/2/8) to Zsolt Boldogkői; by the National Research, Development and Innovation Office (NKFIH OTKA FK 128 252), by the Eötvös Scholarship of the Hungarian State and by a Bolyai János Scholarship of the Hungarian Academy of Sciences to Dóra Tombácz. The project was also supported by the National Institutes of Health, Centers of Excellence in Genomic Science Center for Personal Dynamic Regulomes (5P50HG00773502) to Michael Snyder

## Author contributions

D.T., D.B., M.S., and Z.B. conceived and designed the experiments. D.B. propagated the cells and viruses. D.T. and I.P. prepared RNA samples and generated cDNAs. D.T. prepared the sequencing libraries and performed the PacBio and ONT sequencing. D.T., A.S., I.P., and Z.B. analyzed the data. D.T. and Z.B. wrote the manuscript. Z.B. supervised the project. All authors read and approved the final version of the manuscript.

## Supplementary Material

giga-d-18-00175_original_submission.pdf

giga-d-18-00175_revision_1.pdf

giga-d-18-00175_revision_2.pdf

response_to_reviewer_comments_original_submission.pdf

response_to_reviewer_comments_revision_1.pdf

reviewer_1_report_(original_submission) -- Zhilong Yang, Ph.D 6/18/2018 Reviewed

reviewer_1_report_revision_1 -- Zhilong Yang, Ph.D 10/8/2018 Reviewed

reviewer_2_report_(original_submission) -- Rachael Workman 6/25/2018 Reviewed

reviewer_2_report_revision_1 -- Rachael Workman 10/8/2018 Reviewed

Supplemental Files
