# Supplementary material for: Dynamic transcriptome profiling dataset of vaccinia virus obtained from long-read sequencing techniques
Source: Gigascience. 2018 Nov 23;7(12):giy139. doi: 10.1093/gigascience/giy139 (PMC6290886; doi:10.1093/gigascience/giy139)
Supplement: giga-d-18-00175_original_submission.pdf [file giy139_giga-d-18-00175_original_submission.pdf]

GigaScience

Dynamic Transcriptome Profiling Dataset of Vaccinia Virus Obtained from Long-read Sequencing Techniques

--Manuscript Draft--

**Manuscript Number:**	GIGA-D-18-00175
**Full Title:**	Dynamic Transcriptome Profiling Dataset of Vaccinia Virus Obtained from Long-read Sequencing Techniques
**Article Type:**	Data Note
**Funding Information:**	Swiss-Hungarian Cooperation Programme (SH/7/2/8) — Prof. Dr. Zsolt Boldogkői<br>Magyar Tudományos Akadémia (Bolyai János Scholarship 2015-18) — Dr. Dóra Tombácz<br>NIH Centers of Excellence in Genomic Science (CEGS) Center for Personal Dynamic Regulomes (5P50HG00773502) — Prof. Dr. Michael Snyder

Abstract:

Background

Poxviruses are large DNA viruses infecting humans and animals. The Vaccinia virus (VACV) has been successfully applied as a live vaccine for immunization against smallpox, which had been eradicated by 1980 as a result of worldwide vaccination. VACV is the prototype of poxviruses in the investigation of the molecular pathogenesis of the virus. VACV is also used as a gene delivery vector. Short-read sequencing methods have revolutionized transcriptomics; however, the frequently used approaches are not efficient in distinguishing between the RNA isoforms and transcript overlaps. Long-read sequencing (LRS) however is capable of solving these challenges. Despite the scientific relevance of VACV, no LRS data have been generated for the VACV transcriptome.

Findings

For the deep characterization of the VACV RNA profile, various LRS platforms and library preparation approaches were applied. The raw reads were mapped to the VACV reference genome (LT966077.1), and also to the host genome (GCA_000409795.2). The applied Pacific Biosciences RSII and Sequel platforms altogether resulted in 190,564 mapped reads of inserts, while we obtained 843,983 aligned reads from the different library preparation methods, using the MinION device from the Oxford Nanopore Technologies.

Conclusions

Our dataset that has been generated by cutting-edge technologies can serve as a valuable resource for the investigation of the dynamic VACV transcriptome, the virus-host interactions and the RNA base modifications. These data can provide useful information for novel gene annotations in the VACV genome. Our dataset can be applied for analyzing the currently available LRS platforms, library preparation methods and bioinformatics pipelines.

**Corresponding Author:**	Zsolt Boldogkői<br>Szegedi Tudomanyegyetem Altalanos Orvostudomanyi Kar<br>Szeged, HUNGARY
**Corresponding Author Secondary Information:**	
**Corresponding Author's Institution:**	Szegedi Tudomanyegyetem Altalanos Orvostudomanyi Kar
**Corresponding Author's Secondary Institution:**	
**First Author:**	Dóra Tombácz

Powered by Editorial Manager® and ProduXion Manager® from Aries Systems Corporation

**First Author Secondary Information:**	
**Order of Authors:**	Dóra Tombácz
	István Prazsák
	Attila Szűcs
	Béla Dénes
	Michael Snyder
	Zsolt Boldogkői
**Order of Authors Secondary Information:**	
**Additional Information:**	
**Question**	**Response**
Are you submitting this manuscript to a special series or article collection?	No
**Experimental design and statistics**<br><br>Full details of the experimental design and statistical methods used should be given in the Methods section, as detailed in our Minimum Standards Reporting Checklist. Information essential to interpreting the data presented should be made available in the figure legends.<br><br>Have you included all the information requested in your manuscript?	Yes
**Resources**<br><br>A description of all resources used, including antibodies, cell lines, animals and software tools, with enough information to allow them to be uniquely identified, should be included in the Methods section. Authors are strongly encouraged to cite Research Resource Identifiers (RRIDs) for antibodies, model organisms and tools, where possible.<br><br>Have you included the information requested as detailed in our Minimum Standards Reporting Checklist?	Yes
**Availability of data and materials**<br><br>All datasets and code on which the conclusions of the paper rely must be either included in your submission or deposited in publicly available repositories (where available and ethically appropriate), referencing such data using	Yes

a unique identifier in the references and in the “Availability of Data and Materials” section of your manuscript.<br><br>Have you have met the above requirement as detailed in our Minimum Standards Reporting Checklist?	

Powered by Editorial Manager® and ProduXion Manager® from Aries Systems Corporation

Manuscript

Click here to download Manuscript GigaScience - Tombacz et al.docx

Dynamic Transcriptome Profiling Dataset of Vaccinia Virus Obtained from Long-read Sequencing Techniques

Dóra Tombácz[1], István Prazsák[1], Attila Szűcs[1], Béla Dénes[2], Michael Snyder[3], Zsolt Boldogkői[1*]

[1]Department of Medical Biology, Faculty of Medicine, University of Szeged, Somogyi B. u. 4., 6720 Szeged, Hungary

[2]Veterinary Diagnostic Directorate of the National Food Chain Safety Office, Keleti Károly u. 24., 1024 Budapest, Hungary

[3]Department of Genetics, School of Medicine, Stanford University, 300 Pasteur Dr, Stanford, California, USA

E-mails:

DT: tombacz.dora@med.u-szeged.hu

IP: prazsak.istvan@med.u-szeged.hu

AS: szucs.attila.1@med.u-szeged.hu

BD: denesb@nebih.gov.hu

MS: mpsnyder@stanford.edu

ZB: boldogkoi.zsolt@med.u-szeged.hu

*Corresponding author: ZB

Abstract

Background Poxviruses are large DNA viruses infecting humans and animals. The Vaccinia virus (VACV) has been successfully applied as a live vaccine for immunization against smallpox, which had been eradicated by 1980 as a result of worldwide vaccination. VACV is the prototype of poxviruses in the investigation of the molecular pathogenesis of the virus. VACV is also used as a gene delivery vector. Short-read sequencing methods have revolutionized transcriptomics; however, the frequently used approaches are not efficient in distinguishing between the RNA isoforms and transcript overlaps. Long-read sequencing (LRS) however is capable of solving these challenges. Despite the scientific relevance of VACV, no LRS data have been generated for the VACV transcriptome.

Findings For the deep characterization of the VACV RNA profile, various LRS platforms and library preparation approaches were applied. The raw reads were mapped to the VACV reference genome (LT966077.1), and also to the host genome (GCA_000409795.2). The applied Pacific Biosciences RSII and Sequel platforms altogether resulted in 190,564 mapped reads of inserts, while we obtained 843,983 aligned reads from the different library preparation methods, using the MinION device from the Oxford Nanopore Technologies.

Conclusions Our dataset that has been generated by cutting-edge technologies can serve as a valuable resource for the investigation of the dynamic VACV transcriptome, the virus-host interactions and the RNA base modifications. These data can provide useful information for novel gene annotations in the VACV genome. Our dataset can be applied for analyzing the currently available LRS platforms, library preparation methods and bioinformatics pipelines.

Keywords: poxvirus, vaccinia virus, long-read sequencing, full-length transcriptome, Pacific Biosciences, RS II system, Sequel system, Oxford Nanopore Technologies, MinION system, direct RNA sequencing

Data Description

Background

Poxviridae is a large virus family that infects vertebrates and invertebrates with highly pathogenic members, such as the variola virus, which is the causative agent of smallpox [1]. Vaccinia virus (VACV) a cowpox virus is the prototypic member of the Orthopoxvirus genus [2]. It is closely related to the *Variola viruses* [3] that had been eliminated due to successes achieved through global vaccination using live VACV. VACV remains to be considered a weapon against potential smallpox outbreaks [4]. VACV has been extensively utilized as an expression and a gene delivery vector [5]; it also serves as a model system for the analysis of virus-host interactions, transcriptional regulation [6], as well as for other molecular biological studies.

Poxviruses, similarly to the Mimiviruses are able to replicate in the cytoplasm of the host cell because they encode the proteins needed for DNA synthesis [7]. They have relatively large (approximately 195 kbp in length), complex double-stranded DNA genomes coding for about 220 proteins. The VACV genes are categorized into three temporary classes: early (E), intermediate (I) and late (L) genes. A study characterized 35 VACV genes as immediate-early (IE) kinetics [8], but this categorization has not been widely accepted. The promoters of genes belonging to different kinetic classes are recognized by stage-specific transcription factors [9, 10, 11, 12]. VACV genes belonging to the same kinetic group have been shown to be clustered in the genome [8]: E genes are located at the ends of the viral genome, while the I and L genes are found in the middle part. Most of the adjacent VACV genes are in the same orientation, while convergent and divergent orientations are uncommon.

Although the extraordinary complexity of the VACV transcriptome has been well-characterized[13, 14, 15,16,1], traditionally used techniques, such as short-read sequencing (SRS), ribosome profiling, cap analysis of gene expression (CAGE), genome tiling [17] etc. cannot span the entire transcript, nor

distinguish between transcript isoforms, bi-, and polycistronic RNA variants, overlapping gene products and embedded RNAs. The transcriptional overlaps generated by the read-through mechanism are very frequent and cause a major problem in the analysis of individual VACV transcripts using traditional approaches. The transcription patterns of VACV genes exhibit an extreme stochasticity; there is an enormous number of transcriptional start sites (TSSs) and transcription end sites (TESs) even within the open reading frames (ORFs). Therefore, it is essential to use full-length sequencing methods to match the transcript ends. These features of transcription are unique; it might represent a form of gene regulation that is unique within living organisms.

Previous studies have already determined the precise 5′ and 3′ ends of VACV transcripts [15, 18], and the putative TSSs and TESs have also been mapped, but the applied methods were not suitable for detecting the entire transcripts at single-molecule level, and therefore it was impossible to determine which transcripts contains certain 5’-ends and 3’-ends.

The Pacific Biosciences (PacBio) Isoform sequencing (Iso-Seq) protocol (using oligo(d)T or random hexamer primer for the reverse transcription), the cDNA sequencing and direct (d)RNA sequencing methods from the Oxford Nanopore Technologies (ONT), as well as the Cap-selection cDNA preparation method (Lexogen) are able to generate full-length transcripts, and thus they can circumvent the limitations of SRS techniques. By using these techniques for cDNA production and library preparation with the PacBio Real-Time Sequencer (RS)II and Sequel, as well as the ONT MinION platforms, we were able to identify several hundreds of novel RNA isoforms (e.g. TSS and TES variants, mono-, bi-, polycistronic transcripts), dozens of coding and non-coding RNAs, and numerous complex transcripts in various herpesviruses [19, 20, 21, 22, 23, 24] and in a baculovirus [25], and we were also able to generate a comprehensive full-length transcript data catalog of VACV.

The present report provides the first long-read, dynamic RNA profiling dataset from the family of Poxviruses and the host cell line (CV-1), which can redefine the VACV transcriptomic landscape. This study is a very large cohort of data from the currently available third-generation sequencing

methods representing the forefront techniques for transcriptome research; therefore, the data presented herein is helpful not only at the molecular level and not just for virologists, but also with respect to general genomics and bioinformatics.

Methods

A detailed workflow about the different library preparation strategies are shown in **Figure 1, 2, 3 and Table 1**.

Table 1.

Run #	Platform	Infection condition	Time Points (h)	RNA sample	RT priming	Cap-selection	PCR	Size selection	Library prep	Barcodes	Base calling
1	RSII	Static	1, 2, 4, 8	PolyaA(+)	Oligo(d)T	no	Yes	no	Iso-seq	no	SMRT Analysis v2.3.0
2	RSII	Static	1, 2, 4, 8	rRNA depletion	Random hexamer	no	Yes	no	Iso-seq	no	SMRT Analysis v2.3.0
3	RSII	Static	1, 2, 4, 8	PolyaA(+)	Oligo(d)T	no	Yes	BluePippin 0.8kb-5kb+	Iso-seq	no	SMRT Analysis v2.3.0
4	RSII	Static	1, 2, 4, 8	PolyaA(+)	Oligo(d)T	no	Yes	BluePippin 0.8-2kb	Iso-seq	no	SMRT Analysis v2.3.0
5	RSII	Static	1, 2, 4, 8	PolyaA(+)	Oligo(d)T	no	Yes	BluePippin 2-3kb	Iso-seq	no	SMRT Analysis v2.3.0
6	RSII	Static	1, 2, 4, 8	PolyaA(+)	Oligo(d)T	no	Yes	BluePippin 3-5kb	Iso-seq	no	SMRT Analysis v2.3.0
7	RSII	Static	1, 2, 4, 8	PolyaA(+)	Oligo(d)T	no	Yes	BluePippin 5kb+	Iso-seq	no	SMRT Analysis v2.3.0
8	Sequel	Dynamic	1	PolyaA(+)	Oligo(d)T	no	Yes	no	Iso-seq	no	SMRT Link v5.0.1.9585
9	Sequel	Dynamic	2	PolyaA(+)	Oligo(d)T	no	Yes	no	Iso-seq	no	SMRT Link v5.0.1.9585
10	Sequel	Dynamic	3	PolyaA(+)	Oligo(d)T	no	Yes	no	Iso-seq	no	SMRT Link v5.0.1.9585
11	Sequel	Dynamic	4	PolyaA(+)	Oligo(d)T	no	Yes	no	Iso-seq	no	SMRT Link v5.0.1.9585
12	Sequel	Dynamic	4	PolyaA(+)	Oligo(d)T	no	Yes	no	Iso-seq	no	SMRT Link v5.0.1.9585
13	Sequel	Dynamic	6	PolyaA(+)	Oligo(d)T	no	Yes	no	Iso-seq	no	SMRT Link v5.0.1.9585
14	Sequel	Dynamic	8	PolyaA(+)	Oligo(d)T	no	Yes	no	Iso-seq	no	SMRT Link v5.0.1.9585
15	Sequel	Dynamic	8	PolyaA(+)	Oligo(d)T	no	Yes	no	Iso-seq	no	SMRT Link v5.0.1.9585
16	MinION	Static	1, 2, 3, 4, 6, 8, 12, 16	PolyaA(+)	Oligo(d)T	no	Yes	Manual Gel 500bp+	1D cDNA	no	Albacore v.2.0.1
17	MinION	Static	1, 2, 3, 4, 6, 8, 12, 16	Total RNA	Oligo(d)T	yes	Yes	no	Teloprime + 1D	no	Albacore v.2.0.1
18	MinION	Static	1, 2, 3, 4, 6, 8, 12, 16	PolyaA(+)	Oligo(d)T	no	No	no	dRNA	no	Albacore v.2.0.1
19	MinION	Dynamic	1	PolyaA(+)	Oligo(d)T	no	Yes	no	1D cDNA	yes	Albacore v.2.0.1
20	MinION	Dynamic	2	PolyaA(+)	Oligo(d)T	no	Yes	no	1D cDNA	yes	Albacore v.2.0.1
21	MinION	Dynamic	3	PolyaA(+)	Oligo(d)T	no	Yes	no	1D cDNA	yes	Albacore v.2.0.1
22	MinION	Dynamic	4	PolyaA(+)	Oligo(d)T	no	Yes	no	1D cDNA	yes	Albacore v.2.0.1
23	MinION	Dynamic	6	PolyaA(+)	Oligo(d)T	no	Yes	no	1D cDNA	yes	Albacore v.2.0.1
24	MinION	Dynamic	8	PolyaA(+)	Oligo(d)T	no	Yes	no	1D cDNA	yes	Albacore v.2.0.1
25	MinION	Dynamic	12	PolyaA(+)	Oligo(d)T	no	Yes	no	1D cDNA	yes	Albacore v.2.0.1

Cells and viruses African green monkey (*Chlorocebus sabaeus*) kidney fibroblast cells [CV-1; American Type Culture Collection (ATCC)] were cultured in RPMI 1640 medium (Sigma-Aldrich) with 10% fetal bovine serum (FBS) and antibiotic-antimycotic solution (Sigma-Aldrich) at 37°C in a humidified 5% CO_2 atmosphere, until confluence was reached. The cells (~2.6×10^7) were washed with serum-free medium before the infection. The highly virulent Western Reserve (WR) VACV strain was used this study. The virus stock was diluted in serum free RPMI 1640 medium, and then it was used (3 ml of VACV at 10 MOI/cell) for the CV-1 infection. Samples were incubated at 37°C in 5% CO_2 atmosphere for 1 h with brief agitation at 10 min intervals to redistribute the virus. Three milliliters of complete growth medium (RPMI 1640 + 10% FBS) was added to the tissue culture flask and the infected cells were further incubated for 1, 2, 4 and 8 hours for RSII sequencing, 1, 2, 3, 4, 6 and 8 hours for Sequel, or 1, 2, 3, 4, 6, 8, 12 and 16 hours for MinION sequencing (**Table 1**) at 37°C in a humidified 5% CO_2 atmosphere. Incubation was followed by rinsing of the cells with serum free RPMI 1640 medium, and then three times freeze-thaw cycles were applied. Cells were scraped into 2ml of phosphate-buffered saline (PSB), and stored at -80°C until use.

RNA Total RNA was purified from the infected cells at various stages of viral infection from 1 to 16 h post-infection (pi) using an RNA Kit from Macherey-Nagel. Polyadenylated RNAs were purified from the cells by using the Oligotex mRNA Mini Kit (Qiagen, **Additional file 1**). For the analysis of non-polyadenylated RNAs, ribodepletion (Epicentre Ribo-Zero™ Magnetic Kit H/M/R) was carried out on the total RNA samples. RNAs were quantified (**Table 2**) by Qubit 2.0 using the Qubit RNA BR Assay Kit for the total RNAs and the Qubit RNA HS Assay Kit for the polyA(+) RNAs (Life Technologies). The quality of the samples was assessed with an Agilent 2100 Bioanalyzer. Samples used had RNA Integrity Numbers greater than 9.5.

Table 2.

Time (h)	A (ng)	B (ng/µl)	C (ng/µl)
1	27,3	410,0	8,1
2	10,0	112,0	9,0

3	18,9	87,8	11,1
4	51,8	460,0	12,1
4	19,9	98,3	6,8
6	20,3	95,0	7,8
8	39,2	460,0	12,0
8	19,6	120,0	6,1

Library preparation for PacBio RSII & Sequel sequencing The cDNAs were generated from the polyA(+) RNA fractions following PacBio's recommendations in the 'Isoform Sequencing (Iso-Seq) using the Clontech SMARTer PCR cDNA Synthesis Kit and No Size Selection' or the BluePippin size-selection protocol (**Figure 1, 2, Table 1**). The samples collected at various time points (1, 4, 8 and 12h pi) were mixed together for the RSII sequencing; however, different time points (1, 2, 3, 4, 6, and 8h pi) were used individually for the production of cDNA libraries for the Sequel method. An rRNA-depleted sample mixture (1, 4, 8 and 12h) was converted to cDNA with modified random hexamer primers (**Table 3**) instead of the SMARTer Kit's oligo(d)T-containing oligo. The detailed library preparation methods are described in our recent publication [24]. Briefly, SMRTbell Template Prep Kit 1.0 was used for SMRTbell library production, followed by primer annealing using the DNA Sequencing Reagent Kit 4.0 v2 and polymerase (DNA Polymerase P6) binding for RSII sequencing, whereas the Sequel Sequencing Kit 2.1 and Sequel DNA Polymerase 2.0 were applied for the Sequel platform. Samples were bounded to magbeads (MagBead Kit v2) for loading onto the PacBio instruments. 240 minutes were set for the RSII movie lengths, while 600 min were applied for the Sequel movie length. One movie was recorded for each SMRT Cell. Seventeen RSII SMRT Cells v3 and 8 Sequel SMRT Cells v2 (SMRT Cell 1M) were used for sequencing. The cDNA samples and the SMRTbell templates were quantified (**Table 2**) by Qubit using Qubit dsDNA HS (High Sensitivity) Assay Kit.

Table 3.

Sequencing method	Name, availability	Catalog #	Sequence (5' -> 3')
PacBio amplified PolyA	3' SMART CDS primer II A - SMARTer PCR cDNA Synthesis Kit (Clontech)	634925 & 634926	AAGCAGTGGTATCAACGCAGAGTAC(T)$_{30}$VN

PacBio amplified Random	Custome made (IDT DNA)	-	AAGCAGTGGTATCAACGCAGAGTACNNNNNN (G: 37%; C: 37%; A: 13%; T: 13%)
MinION cDNA	Poly(T)-containing anchored primer [(VN)T20 - ONT recommended, custom made (Bio Basic)	-	5phos/ ACTTGCCTGTCGCTCTATCTTC(T)$_{20}$VN
MinION CAP selected	TeloPrime Full-Length cDNA Amplification Kit (Lexogen)	013.08 & 013.24	TCTCAGGCGTTTTTTTTTTTTTTTTTT
MinION RNA	RT adapter - Direct RNA Sequencing Kit (Oxford Nanopore Technologies)	SQK-RNA001	GAGGCGAGCGGTCAATTTTCCTAAGAGCAAGAAGAAGCCTTTTTTTTTT

ONT MinION– cDNA sequencing For the cDNA sequencing on the MinION device, the PolyA(+) RNAs were used. One library was prepared from an RNA mixture (RNA samples from the 1, 2, 3, 4, 6, 8, 12 and 16h pi); however, the various time points were sequenced individually as well (**Figure 1, 3**, **Table 4**). The ONT 1D strand switching cDNA by ligation protocol (Version: SSE_9011_v108_revS_18Oct2016), the Ligation Sequencing 1D kit (SQK-LSK108, Oxford Nanopore Technologies) and the NEBNext End repair / dA-tailing Module NEB Blunt/TA Ligase Master Mix (New England Biolabs) were used for the library preparation according the manufacturers' recommendations. The PCR amplicons derived from the mixed RNA sample were size-selected manually, and then ran on Ultrapure Agarose gel (Thermo Fischer Scientific), followed by the isolation of 500bp+ fragments using the Zymoclean Large Fragment DNA Recovery Kit (Zymo Research). The individually sequenced samples were labeled by barcodes applying a combination of two ONT protocols, for the first time: first, the 1D protocol was used, however, after the first end-prep step, we switched to the 1D PCR barcoding (96) genomic DNA (SQK-LSK108) protocol (version: PBGE96_9015_v108_revS_18Oct2016, updated 25/10/2017), which was then followed by the barcode ligation step. The ONT PCR Barcoding Kit 96; EXP-PBC096 was used. The quantities of the libraries were measured by Qubit 2.0 (**Table 4**).

Table 4.

Library	Starting RNA	Starting RNA amount (ng)	cDNA amount (PCR product, ng)	Library used for sequencing (ng)	Barcode #	Number of flow cells
1D cDNA	polyA(+) mixed	29	253	65	-	1
1D cDNA	polyA(+) mixed	29	251	48	-	
1D cDNA	polyA(+) 1h	50	117	150	A1	1
1D cDNA	polyA(+) 2h	50	387		A2	
1D cDNA	polyA(+) 3h	50	360	300	A3	1

1D cDNA	polyA(+) 4h	50	180		A4	
1D cDNA	polyA(+) 6h	50	207		A5	
1D cDNA	polyA(+) 8h	50	103		A6	
1D cDNA	polyA(+) 12h	50	130		A7	
dRNA	polyA(+) mixed	60	no PCR	10,2	-	1
Cap-seq	total RNA (1, 2, 3h)	2 µg	240	240	-	1
Cap-seq	total RNA (4, 6, 8, 12, 16h)	2 µg	1125	320	-	1

ONT MinION cDNA-sequencing on Cap-selected samples For more precise identification of the 5'-ends of the full-length transcripts, a Cap-selection method was applied and combined with the ONT 1D cDNA library preparation protocol. The cDNAs were generated from a mixed total RNA sample (containing RNA from 1, 2, 3, 4, 6, 8, 12 and 16h pi, **Table 1 and 4**) by using the TeloPrime Full-Length cDNA Amplification Kit (Lexogen). The protocol contains a PCR amplification step, the specificity of the products was checked by qPCR (Rotor-Gene Q). A VACV gene specific primer (D1R gene, **Table 5**) and ABsolute qPCR SYBR Green Mix (Thermo Fisher Scientific) was used. The amplified, PolyA(+)- and Cap-selected samples were subjected to the ONT's 1D Strand switching cDNA by ligation method (ONT Ligation Sequencing 1D kit); they were end-repaired, then ligated to the 1D adapters (NEBNext End repair / dA-tailing Module NEB Blunt/TA Ligase Master Mix)

Table 5.

	5' →3'
fw	CGAACTAGAGGACCGTTGGG
rev	TTTCCAGGTCAGCACCGTTT

ONT MinION – dRNA sequencing In order to avoid the potential PCR biases, the amplification-free Direct RNA sequencing (DRS) protocol (Version: DRS_9026_v1_revM_15Dec2016) protocol from the ONT's was applied. The library was prepared from a PolyA(+) mixture of 8 time points (1, 2, 3, 4, 6, 8, 12 and 16h pi, **Table 4**).

The ONT cDNA libraries, the Cap-selected samples, and the direct RNA libraries were loaded on 3, 2 and 1 ONT R9.4 SpotON Flow Cells for sequencing, respectively. The runs were carried out using MinKNOW. Voltage levels were set and reset in line with the suppliers' recommendations.

Data analysis and visualization

The PacBio RSII reads of insert (ROI) reads were generated using the RS_ReadsOfInsert protocol of the SMRT Analysis v2.3.0, with the following settings: Minimum Full Passes = 1, Minimum Predicted Accuracy = 90, Minimum Length of Reads of Insert = 1, Maximum Length of Reads of Insert = No Limit. These consensus reads were mapped using GMAP [26] (version 2017-09-30), with default settings. The ROIs from the Sequel data were created by using SMRT Link 5.0.1.9585. For the MinION base calling, the ONT's Albacore software v.2.0.1 was applied. This basecaller is able identify the nucleotide sequences directly from raw sequencing data. The reads were aligned with GMAP program using the same setting as described above. The raw reads were aligned to the reference genome of the virus (LT966077.1) and the host cell (*Chlorocebus sabaeus*): GenBank assembly accession: GCA_000409795.2 (latest); RefSeq assembly accession: GCF_000409795.2 (latest)]. In-house routines were used to acquire the quality information presented in this data note. The codes have been archived on Github [27]. Bedtools genomecov software [28] was used to generate coverage files with these following parameters: -split – ibam. The output bed files were visualized by Circos plot [29] (**Figure 4**).

Data summary

The raw sequencing reads were mapped to the VACV reference genome, as well as to the host genome. In this study, we generated full-length transcripts of VACV and the CV-1 cells, yielding about 0.75 Gb of mapped sequencing data. Sequencing on the RSII and Sequel platforms yielded 71,008 and 119,556 ROIs, aligned to the viral and the host genome, respectively. The utilized nanopore-based cDNA sequencing approaches resulted in altogether 390,781 VACV specific reads (**Table 6**, **Table 7**), while we obtained 140,243 reads from the Cap-selected samples. The average

lengths of ROIs aligning to the VACV genome were 1,103 bp for PacBio RSII, 1,212 bp for the Sequel. The MinION average read lengths are as follows: 550 bp for ONT barcoded cDNA sequencing, 783bp for the cDNA-seq, and 966 bp for the Cap-selected samples (**Table 6**). The average read length produced by the direct RNA sequencing is 535bp. It should be noted that the library preparation and size-selection methods resulted in different samples in length; all library preparation methods resulted in longer average read length aligning to the host genome than to the viral genome (**Table 6, Table 7, Figure 5, Figure 6 and Figure 7**). Our transcriptomic survey yielded in an extreme high coverage across the viral genome (**Figure 4**): 261 fold for the RSII, 135.5 fold for the Sequel, 544 fold for the barcoded MinION cDNA-seq, 501 fold for the Cap-selected samples and 289 fold for the cDNA sequencing (more detailed information, including quality information are available in **Table 6, Table 7 and Additional file 2 and 3**) and data shows that the entire VACV genome is transcriptionally active, generating RNAs from both DNA strands. Our dataset also contains 1.56 Gb of raw data from Sequel sequencing, as well as from MinION dRNA-sequencing.

Table 6.

Run	Specificities	Mapped reads	Avg read length ± SE	Avg aligning read length ± SE	Avg insertion frequency ± SE	Avg deletion frequency ± SE	Avg mismatch frequency ± SE	Coverage
1	RSII mix no size selection	110	1385.66 ± 46.40	1361.50 ± 46.58	0.04% ± 0.0096%	0.22% ± 0.0603%	0.02% ± 0.0056%	0.77
2	RSII mix random primed	31	799.74 ± 88.58	714.87 ± 89.19	0.09% ± 0.0447%	0.14% ± 0.0634%	0.02% ± 0.0117%	0.11
3	RSII BluePippin: 0.8-5kb+	19176	1146.55 ± 10.20	938.74 ± 3.52	0.25% ± 0.0096%	0.30% ± 0.0063%	0.10% ± 0.0042%	92.37
4	RSII BluePippin: 0.8-2kb	127	1104.09 ± 10.98	978.17 ± 9.67	0.08% ± 0.0222%	0.15% ± 0.0134%	0.08% ± 0.0929%	6.37
5	RSII BluePippin: 2-3kb	4907	1063.88 ± 5.87	949.30 ± 5.31	0.12% ± 0.0071%	0.11% ± 0.0059%	0.03% ± 0.0050%	23.90
6	RSII BluePippin: 3-5kb	19775	1062.12 ± 2.82	946.58 ± 2.6	0.14% ± 0.0038%	0.16% ± 0.0043%	0.04% ± 0.0032%	96.05
7	RSII BluePippin: 5kb+	8	1122.71 ± 4.95	1027.33 ± 4.7	0.11% ± 0.1584%	0.19% ± 0.0065%	0.04% ± 0.1015%	42.17
8	Sequel 1h	413	1124.87 ± 40.39	773.88 ± 23.99	0.32% ± 0.0607%	0.18% ± 0.0569%	0.50% ± 0.1350%	1.64
9	Sequel 2h	489	1064.17 ± 30.40	828.46 ± 24.18	0.20% ± 0.0487%	0.10% ± 0.0124%	0.29% ± 0.0997%	2.08
10	Sequel 3h	1049	1118.35 ± 19.57	908.10 ± 15.12	0.21% ± 0.0467%	0.11% ± 0.0091%	0.26% ± 0.0607%	4.89
11	Sequel 4h	786	1112.09 ± 36.40	725.07 ± 20.15	0.33% ± 0.0679%	0.14% ± 0.0144%	0.46% ± 0.0969%	2.92
12	Sequel 4h 2nd	4405	1216.98 ± 10.28	966.28 ± 7.02	0.49% ± 0.0212%	0.18% ± 0.0058%	0.47% ± 0.0381%	21.84
13	Sequel 6h	2967	1120.25 ± 13.76	864.78 ± 10.13	0.23% ± 0.0186%	0.10% ± 0.0050%	0.25% ± 0.0344%	13.17

14	Sequel 8h	5386	1176.00 ± 9.67	982.33 ± 8.07	0.21% ± 0.0135%	0.11% ± 0.0042%	0.12% ± 0.0143%	27.15
15	Sequel 8h 2nd	11379	1276.74 ± 6.89	1059.17 ± 5.53	0.26% ± 0.0094%	0.13% ± 0.0032%	0.14% ± 0.0091%	61.84
16	MinION cDNA Manual: 500bp+	86565	783.23 ± 1.90	651.58 ± 1.67	3.25% ± 0.0063%	6.03% ± 0.0069%	6.61% ± 0.0093%	289.42
17	MinION dRNA	1214	534.60 ± 10.01	522.86 ± 10.74	1.98% ± 0.0537%	8.80% ± 0.0839%	5.56% ± 0.0634%	3.26
18	MinION Cap-selection	140243	965.91 ± 1.38	696.77 ± 0.78	3.28% ± 0.0049%	5.30% ± 0.0045%	6.73% ± 0.0069%	501.40
19	MinION cDNA barcoded 1h	1694	587.50 ± 2.19	390.83 ± 1.99	2.96% ± 0.0496%	4.54% ± 0.0170%	4.62% ± 0.0590%	31.02
20	MinION cDNA barcoded 2h	93753	517.68 ± 0.68	334.28 ± 0.59	3.05% ± 0.0071%	4.57% ± 0.0073%	4.66% ± 0.0081%	146.83
21	MinION cDNA barcoded 3h	21922	521.16 ± 1.32	337.50 ± 1.18	2.81% ± 0.0138%	4.39% ± 0.0142%	4.51% ± 0.0163%	34.66
22	MinION cDNA barcoded 4h	41203	523.76 ± 1.00	340.99 ± 0.90	3.02% ± 0.0124%	4.46% ± 0.0106%	4.64% ± 0.120%	65.82
23	MinION cDNA barcoded 6h	41664	568.71 ± 1.27	382.47 ± 1.18	2.84% ± 0.0110%	4.47% ± 0.0107%	4.88% ± 0.0119%	74.66
24	MinION cDNA barcoded 8h	47775	646.14 ± 1.69	446.08 ± 1.55	2.93% ± 0.0109%	4.37% ± 0.0098%	4.70% ± 0.0110%	99.84
25	MinION cDNA barcoded 12h	56205	526.12 ± 0.85	346.09 ± 0.88	2.85% ± 0.0093%	4.48% ± 0.0089%	4.55% ± 0.0101%	91.13

Table 7.

**Run #**	**Specificities**	**Mapped reads**	**Avg read length ± SE**	**Avg aligning read length ± SE**	**Avg insertion frequency ± SE**	**Avg deletion frequency ± SE**	**Avg mismatch frequency ± SE**
8	Sequel 1h	8720	1506.64 ± 9.30	1157.06 ± 9.33	0.44% ± 0.0183%	0.33% ± 0.0149%	1.14% ± 0.0231%
9	Sequel 2h	8192	1362.82 ± 9.42	1234.66 ± 8.67	0.35% ± 0.0151%	0.18% ± 0.0058%	1.18% ± 0.0217%
10	Sequel 3h	25592	1551.37 ± 5.75	1389.94 ± 5.02	0.52% ± 0.0111%	0.21% ± 0.0034%	0.57% ± 0.0072%
12	Sequel 4h 2nd	12695	1521.64 ± 9.92	1259.80 ± 7.50	0.63% ± 0.0185%	0.27% ± 0.0064%	0.72% ± 0.0125%
13	Sequel 6h	26481	1626.59 ± 5.80	1461.76 ± 5.18	0.51% ± 0.0109%	0.20% ± 0.0031%	0.80% ± 0.0093%
14	Sequel 8h	21922	1745.01 ± 6.57	1622.62 ± 6.46	0.44% ± 0.0113%	0.20% ± 0.0037%	1.51% ± 0.0147%
15	Sequel 8h 2nd	15954	1782.93 ± 6.78	1247.91 ± 7.95	0.97% ± 0.0398%	0.27% ± 0.0094%	0.68% ± 0.0112%
19	MinION cDNA barcoded 1h	23250	937.70 ± 3.88	709.25 ± 3.84	3.37% ± 0.0201%	5.27% ± 0.0195%	5.58% ± 0.0167%
20	MinION cDNA barcoded 2h	210190	788.71 ± 1.23	584.11 ± 1.21	3.29% ± 0.0066%	5.27% ± 0.0064%	5.44% ± 0.0056%
21	MinION cDNA barcoded 3h	4654	819.90 ± 7.38	609.87 ± 7.43	2.89% ± 0.0405%	5.25% ± 0.0433%	5.85% ± 0.0435%
22	MinION cDNA barcoded 4h	42750	732.19 ± 2.31	524.55 ± 2.29	3.03% ± 0.0152%	5.11% ± 0.0147%	5.19% ± 0.0129%
23	MinION cDNA barcoded 6h	16076	763.49 ± 3.99	546.83 ± 3.98	3.13% ± 0.0263%	5.11% ± 0.0239%	5.30% ± 0.0218%
24	MinION cDNA barcoded 8h	8789	853.27 ± 5.83	566.24 ± 6.03	3.01% ± 0.0448%	4.69% ± 0.0377%	5.04% ± 0.0339%
25	MinION cDNA barcoded 12h	6036	642.62 ± 4.69	307.90 ± 5.25	2.95% ± 0.0787%	3.97% ± 0.0555%	4.11% ± 0.0464%

Conclusion and Reuse Potential

The present study, derived from state-of-art sequencing technologies (PacBio RSII and Sequel, as well as the ONT MinION platforms, applying a new protocol for barcoding the samples), provides a time-course look at the full-length transcriptome of VACV, as well as to the CV-1 host cell line.

The dataset was primarily generated for the dynamic characterization of VACV transcriptome. Another aim was to generate a deep coverage long-read dataset for the analysis of the different transcript isoforms, including length- (5', 3'-ends) variants, mono-, bi-, polycistronic transcripts, as well as to define the exact transcripts produced by the various viral genes. This dataset helps to understand the complexity of the VACV. The provided dataset can also be used to investigate the effect of the viral infection on the gene expression level of the host.

The provided binary alignment (BAM) files contain reads already aligned to the VACV and to the host genome. These aligned reads can be further analyzed by using different long-read aligners (e.g. BLASR [30,]; NGMLR [31]), and bioinformatics tools (e.g. samtools [32] or bedtools [33]). These data can be visualized by using different programs such as the Geneious [34], Artemis [35], or IGV [36]. Data can be useful to test novel bioinformatics pipelines or to improve the currently available ones. The files contain terminal poly(A) sequences as well as the 5'and 3' adapter sequences, which can be utilized to determine the orientations of the reads. The dataset contains the raw dataset from dRNA sequencing (fast5.tar.gz), which can be further analyzed by using Tombo software package [37], which enables the detection and visualization of modified nucleotides, such as the most widespread of epigenetic modifications: 5-methyl cytosine (5mC), or the less frequent base modifications, e.g. 6-methyl adenine (6mA). The raw data provided from PacBio Sequel sequencing can be used to improve existing base caller algorithms or potentially to develop novel algorithms, and further, the data contain the full set of quality values and kinetic measurements.

This dataset can be used to identify novel VACV and CV-1 transcripts and RNA isoforms (splice variants of the host transcripts, TSS and TES variants and polycistronic transcripts of the virus and the host), to examine de effect the viral infection on the expression level host genes, as well as for the comparison of the quality and length of the sequencing reads derived from different sequencing platforms. The utilized various library preparation methods can also be compared with one another.

The data could lead to increasing our knowledge on the particular gene expression profile of Poxviruses, and also can be used to design gene expression vectors.

Availability of Supporting Data

All of the presented data was deposited in the European Nucleotide Archive under the accession number of PRJEB26434 (Characterization of the Vaccinia virus transcriptome) and PRJEB26430 (Dynamic characterization of the Vaccinia virus transcriptome).

Competing interests

The authors declare that there are no conflicts of interest.

Funding

This study was supported by the Swiss-Hungarian Cooperation Programme [SH/7/2/8] to ZB, by the Eötvös Scholarship of the Hungarian State to DT and by Bolyai János Scholarship of the Hungarian Academy of Sciences to DT. The project was also supported by the NIH Centers of Excellence in Genomic Science (CEGS) Center for Personal Dynamic Regulomes [5P50HG00773502] to MS.

Author Contributions

DT, DB, MS and ZB conceived and designed the experiments. DB propagated the cells and viruses. DT and IP prepared RNA samples and generated cDNAs. DT prepared the sequencing libraries and performed the PacBio and ONT sequencing. AS DT, IP and ZB analyzed the data. DT and ZB wrote the manuscript. ZB supervised the project. All authors have read and approved the final version of the manuscript.

Abbreviations

5mC - 5-methyl cytosine

6mA - 6-methyl adenine

CV-1 - African green monkey (*Chlorocebus sabaeus*) kidney fibroblast cells

ATCC - American Type Culture Collection

CAGE - cap analysis of gene expression

dRNA - direct RNA

E - early

FBS - fetal bovine serum

IE - immediate-early

I - intermediate

Iso-Seq - Isoform sequencing

L - late

LRS - Long-read sequencing

ORF - open reading frame

ONT - Oxford Nanopore Technologies

PacBio - Pacific Biosciences

PBS - phosphate-buffered saline

pi - post-infection

RSII - Real-Time Sequencer II

SRS - short-read sequencing

TES - transcription end site

TSS - transcriptional start sites

VACV - Vaccinia virus

WR - Western Reserve

References

1. Yang Z, Cao S, Martens CA et al. Deciphering Poxvirus Gene Expression by RNA Sequencing and Ribosome Profiling. *J Virol*. 2015;89(13): 6874–6886. doi: 10.1128/JVI.00528-15

2. Benfield CT, Ren H, Lucas SJ et al. Vaccinia virus protein K7 is a virulence factor that alters the acute immune response to infection. *J Gen Virol*. 2013;94(Pt 7): 1647–1657. doi: 10.1099/vir.0.052670-0

3. Pauli G, Blümel J, Burger R et al. Orthopox Viruses: Infections in Humans. *Transfus Med Hemother*. 2010;37(6): 351–364. doi: 10.1159/000322101

4. Moutaftsi M, Tscharke DC, Vaughan K et al. Uncovering the interplay between CD8, CD4 and antibody responses to complex pathogens. *Future Microbiol*. 2010;5(2):221-239. doi: 10.2217/fmb.09.110

5. Wyatt LS, Xiao W, Americo JL et al. Novel Nonreplicating Vaccinia Virus Vector Enhances Expression of Heterologous Genes and Suppresses Synthesis of Endogenous Viral Proteins. *mBio*. 2017;8(3): e00790-17. doi: 10.1128/mBio.00790-17

6. Broyles SS. Vaccinia virus transcription. *J Gen Virol*. 2003;84:2293–2303. doi: 10.1099/vir.0.18942-0

7. Schramm B, Locker JK. Cytoplasmic Organization of POXvirus DNA Replication. *Traffic* 2005;6:839–846. doi: 10.1111/j.1600-0854.2005.00324.x

8. Assarsson E, Greenbaum JA, Sundström M et al. Kinetic analysis of a complete poxvirus transcriptome reveals an immediate-early class of genes. *Proc Natl Acad Sci U S A*. 2008;105(6):2140-5. doi: 10.1073/pnas.0711573105.

9. Davison AJ, Moss B. Structure of vaccinia virus early promoters. *J Mol Biol*. 1989; 210(4):749–769.

10. Davison AJ, Moss B. Structure of vaccinia virus late promoters. *J Mol Biol*. 1989; 210(4):771–784.

11. Baldick CJ, Jr, Keck JG, Moss B. Mutational analysis of the core, spacer, and initiator regions of vaccinia virus intermediate-class promoters. *J Virol*. 1992;66:4710–4719.

12. Broyles SS, Moss B. Homology between RNA polymerases of poxviruses, prokaryotes, and eukaryotes: nucleotide sequence and transcriptional analysis of vaccinia virus genes encoding 147-kDa and 22-kDa subunits. *Proc Natl Acad Sci U S A*. 1986;83(10):3141-5.

13. Wittek R, Cooper JA, Barbosa E et al. Expression of the vaccinia virus genome: Analysis and mapping of mRNAs encoded within the inverted terminal repetition. *Cell*. 1980;21(2):487–493.

14. Yang Z, Bruno DP, Martens CA et al. Simultaneous high-resolution analysis of vaccinia virus and host cell transcriptomes by deep RNA sequencing. *PNAS*. 2010;107(25):11513-11518. https://doi.org/10.1073/pnas.1006594107

15. Yang Z, Bruno DP, Martens CA et al. Genome-Wide Analysis of the 5′ and 3′ Ends of Vaccinia Virus Early mRNAs Delineates Regulatory Sequences of Annotated and Anomalous Transcripts. *J Virol*. 2011;85(12): 5897–5909. doi: 10.1128/JVI.00428-11

16. Yang Z, Maruri-Avidal L, Sisler J et al. Cascade regulation of vaccinia virus gene expression is modulated by multistage promoters. *Virology* 2013;447(1–2):213-220. doi.org/10.1016/j.virol.2013.09.007

17. Rubins KH, Hensley LE, Bell GW et al. Comparative analysis of viral gene expression programs during poxvirus infection: a transcriptional map of the vaccinia and monkey pox genomes. *PLoS One*. 2008;3(7):e2628. 10.1371/journal.pone.0002628

18. Yang Z, Martens CA, Bruno DP et al. Pervasive initiation and 3′ end formation of poxvirus post-replicative RNAs. *J Biol Chem*. 2012;287:31050–31060. doi: 10.1074/jbc.M112.390054.

19. Tombácz D, Csabai Z, Oláh P et al. Full-Length Isoform Sequencing Reveals Novel Transcripts and Substantial Transcriptional Overlaps in a Herpesvirus. *PLoS One*. 2016;11(9) e0162868. doi: 10.1371/journal.pone.0162868.

20. Tombácz D, Csabai Z, Szűcs A et al. Long-Read Isoform Sequencing Reveals a Hidden Complexity of the Transcriptional Landscape of Herpes Simplex Virus Type 1. *Front Microbiol*. 2017;8:1079. doi: 10.3389/fmicb.2017.01079.

21. Balázs Z, Tombácz D, Szűcs A et al. Long-Read Sequencing of Human Cytomegalovirus Transcriptome Reveals RNA Isoforms Carrying Distinct Coding Potentials. *Sci Rep*. 2017;7(1):15989. doi: 10.1038/s41598-017-16262-z.

22. Balázs Z, Tombácz D, Szűcs A et al. Long-read sequencing of the human cytomegalovirus transcriptome with the Pacific Biosciences RSII platform. *Sci Data*. 2017;4:170194. doi: 10.1038/sdata.2017.194.

23. Moldován N, Tombácz D, Szűcs A et al. Multi-Platform Sequencing Approach Reveals a Novel Transcriptome Profile in Pseudorabies Virus. *Front Microbiol*. 2018;8:2708. doi: 10.3389/fmicb.2017.02708.

24. Tombácz D, Sharon D, Szűcs A et al. Transcriptome-wide survey of pseudorabies virus using next- and third-generation sequencing platforms. *Sci Data*. 2018. in press

25. Moldován N, Tombácz D, Szűcs A et al. Third-generation Sequencing Reveals Extensive Polycistronism and Transcriptional Overlapping in a Baculovirus. *Sci. Rep*. under review

26. Wu TD, Watanabe CK. GMAP: a genomic mapping and alignment program for mRNA and EST sequences. *Bioinformatics*. 2005;21(9):1859–75. doi: 10.1093/bioinformatics/bti310

27. Long-read sequencing data statistics. doi: 10.5281/zenodo.1034511. Accessed 21 Oct 2017.

28. Quinlan AR. BEDTools: The Swiss-Army Tool for Genome Feature Analysis. *Curr Protoc Bioinformatics*. 2014;47:11.12.1-34. doi: 10.1002/0471250953.bi1112s47.

29. Krzywinski M, Schein J, Birol I et al. Circos: an information aesthetic for comparative genomics. *Genome Res*. 2009;19(9):1639-45. doi: 10.1101/gr.092759.109.

30. Chaisson M, Tesler G. Mapping single molecule sequencing reads using Basic Local Alignment with Successive Refinement (BLASR): Theory and Application. *BMC Bioinformatics*. 2012;13:238. doi: 10.1186/1471-2105-13-238.

31. Sedlazeck FJ, Rescheneder P, Smolka M et al. Accurate detection of complex structural variations using single-molecule sequencing. *Nat Methods*. 2018; doi: 10.1038/s41592-018-0001-7.

32. Li H, Handsaker B, Wysoker A et al. The Sequence Alignment/Map format and SAMtools. *Bioinformatics.* 2009;25(16):2078–2079. doi: 10.1093/bioinformatics/btp352.

33. Quinlan AR, Hall I. M. BEDTools: a flexible suite of utilities for comparing genomic features. *Bioinformatics.* 2010;26(6):841–842. doi: 10.1093/bioinformatics/btq033.

34. Kearse M, Moir R, Wilson A et al. Geneious Basic: an integrated and extendable desktop software platform for the organization and analysis of sequence data. *Bioinformatics*. 2012;28(12):1647–1649. doi: 10.1093/bioinformatics/bts199.

35. Rutherford K, Parkhill J, Crook J et al. Artemis: sequence visualization and annotation. *Bioinformatics.* 2010;16(10):944-5.

36. Robinson, J. T. *et al.* Integrative genomics viewer. *Nat. Biotechnol.***29**, 24–26 (2011).

37. Stoiber MH, Quick J, Egan R et al. De novo Identification of DNA Modifications Enabled by Genome-Guided Nanopore Signal Processing. *bioRxiv*. 2017;094672. doi: https://doi.org/10.1101/094672

Legend to Figures

Figure 1. Flowchart diagram shows an overview of the experimental design

Figure 2. Detailed layout of the PacBio wet-lab experiments

Figure 3. Comprehensive experimental workflow of the MinION sequencing

Figure 4. The Circos plot showing the genome-wide transcriptome profile of VACV. The gray boxes represent the ORFs. Data derived from the five different library preparation and sequencing methods used in this study are shown on the histogram as follows: green: Sequel all data (data from different time points are mixed together); blue: RSII mixed sample; yellow: MinION 1D cDNA mixed sample; orange: MinION Cap-selected mixed sample; black: MinION 1D cDNA barcoded all data (data from different time points are mixed together).

Figure 5. The average read lengths aligned to the viral genome in the various library preparation and sequencing methods. Error bars represent the standard deviance (SD).

Figure 6. The average read lengths mapped to the host genome in the different library preparation and sequencing techniques. Error bars represent the standard deviance (SD).

Figure 7. The comparative bar chart shows the differences between the average lengths of sequencing reads derived from the transcripts of VACV and the host cell at the same samples. Data show that the host cell transcripts are 1.19 to 1.38-fold longer than the VACV transcripts.

Tables

Table 1. Summary table of the different wet lab approaches applied in this study.

Table 2. Summary table of the amount of RNA, cDNA and library samples used for PacBio Sequel sequencing: A: amount of PolyA(+) RNA used for cDNA preparation. B: concentration of obtained PCR products. C: concentration of SMRTbell libraries

Table 3. The list of primers sequences used in this study for the reverse transcription reactions

Table 4. Summary table of the amount of RNA, cDNA and library samples used for ONT MinION sequencing.

Table 5. Sequence of the gene-specific primer pair used for the amplification of *D1R* gene of VACV

Table 6. Summary statistics of the sequencing reads which mapped to the viral genome from each run. SE: standard error

Table 7. Summary statistics of the sequencing reads which aligned to the host reference genome from each run of individual time points. SE: standard error

Additional Files

Additional file 1. Summary table of the reagents and chemistries used for the sequencing.

Additional file 2. Summary statistics of the viral reads from each run.

Additional file 3. Summary statistics of the host reads from each run.

Figure 1

Click here to download Figure Fig1.pdf

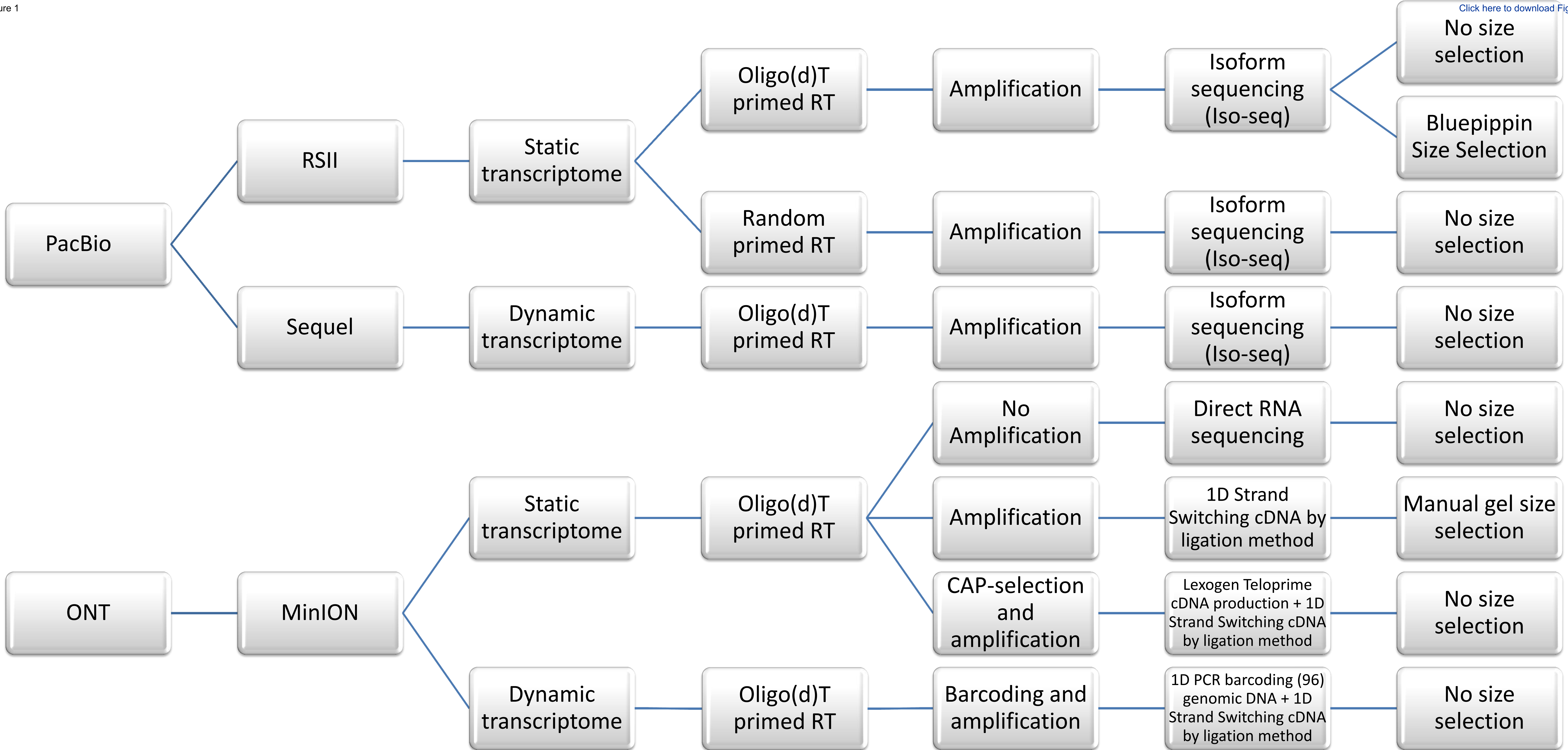

Click here to download Figure Fig2.pdf

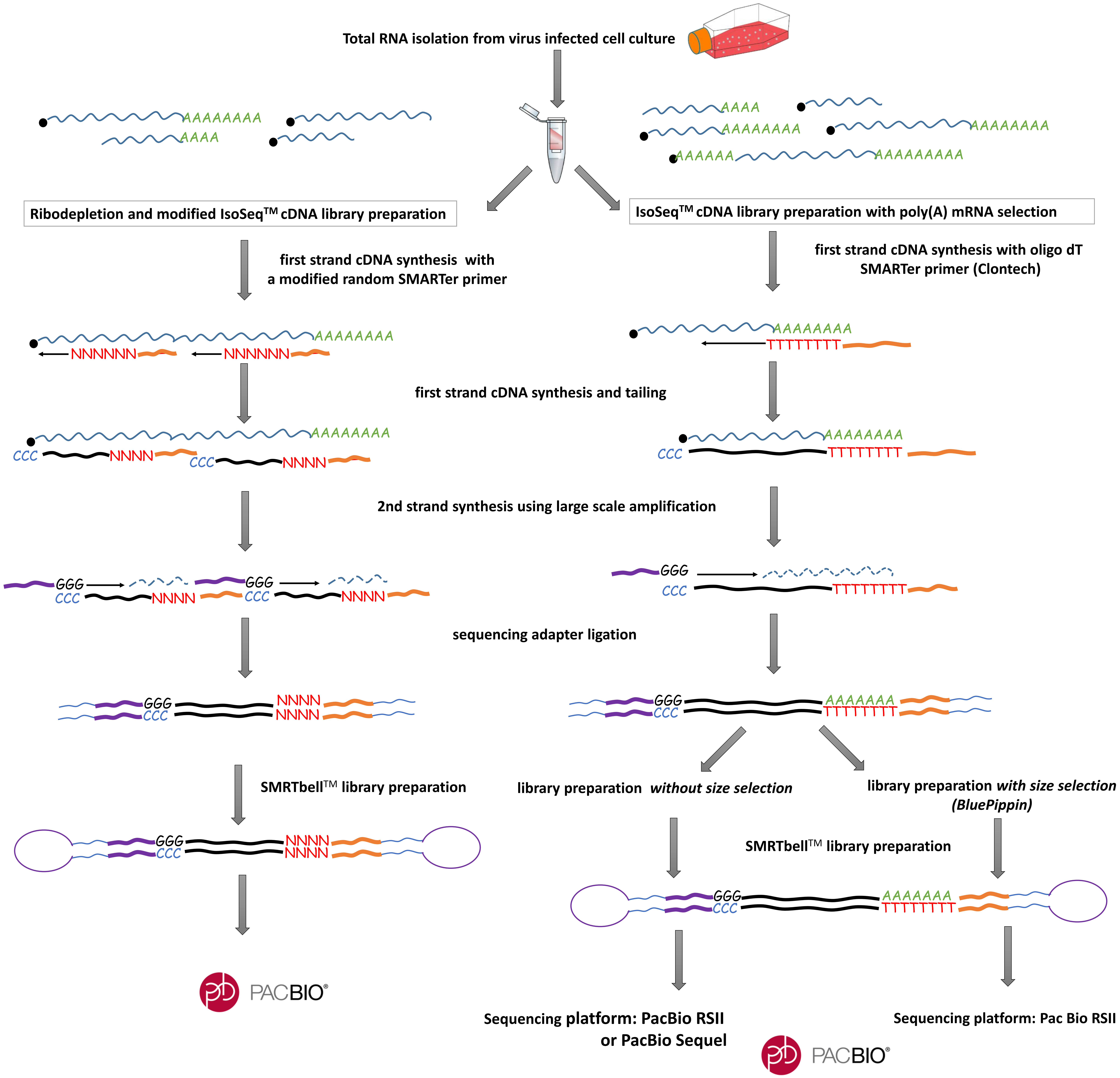

Click here to download Figure Fig3.pdf

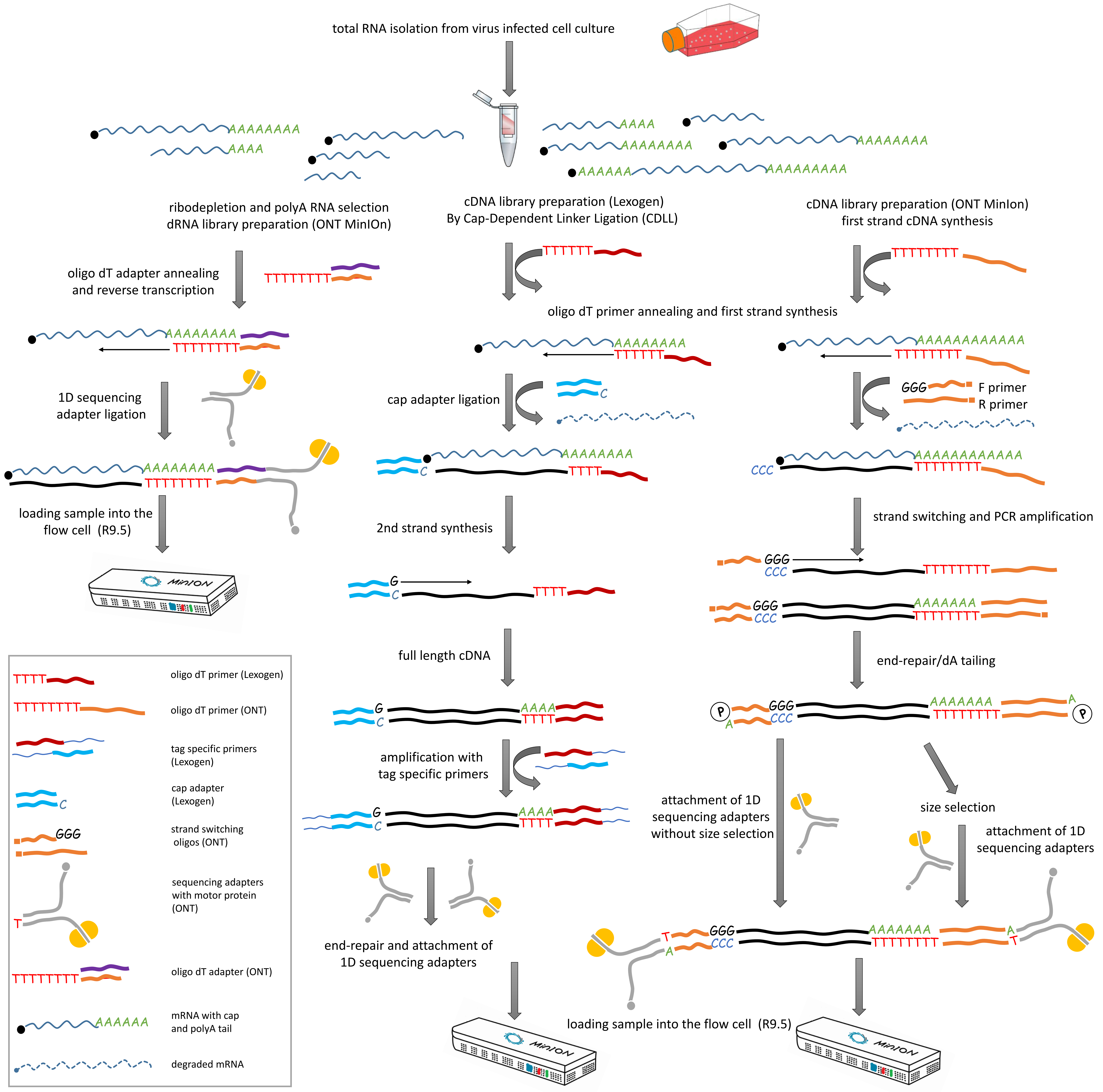

Click here to download Figure Fig4.pdf

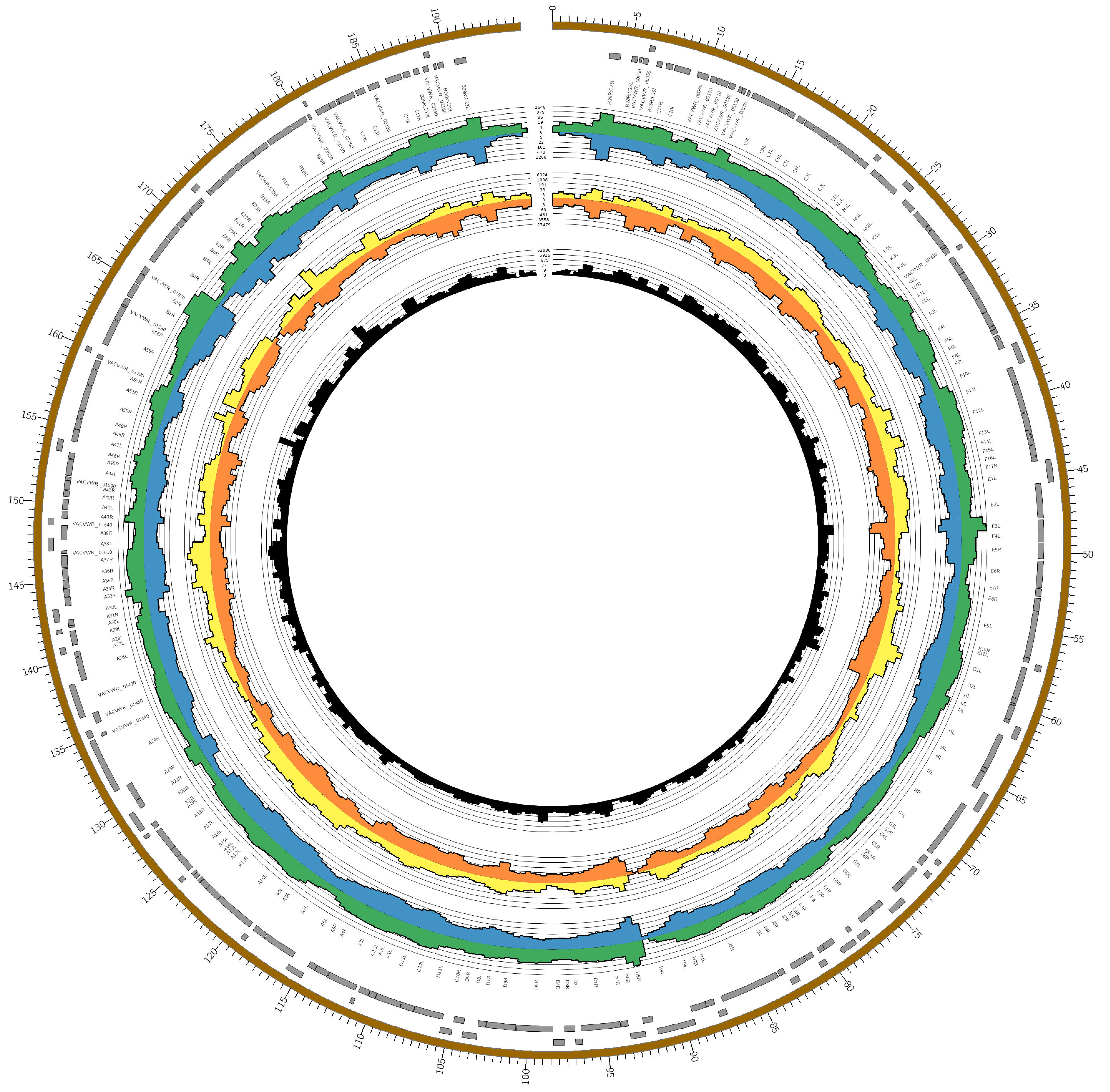

LT966077

Figure 5

Click here to download Figure Fig5.pdf

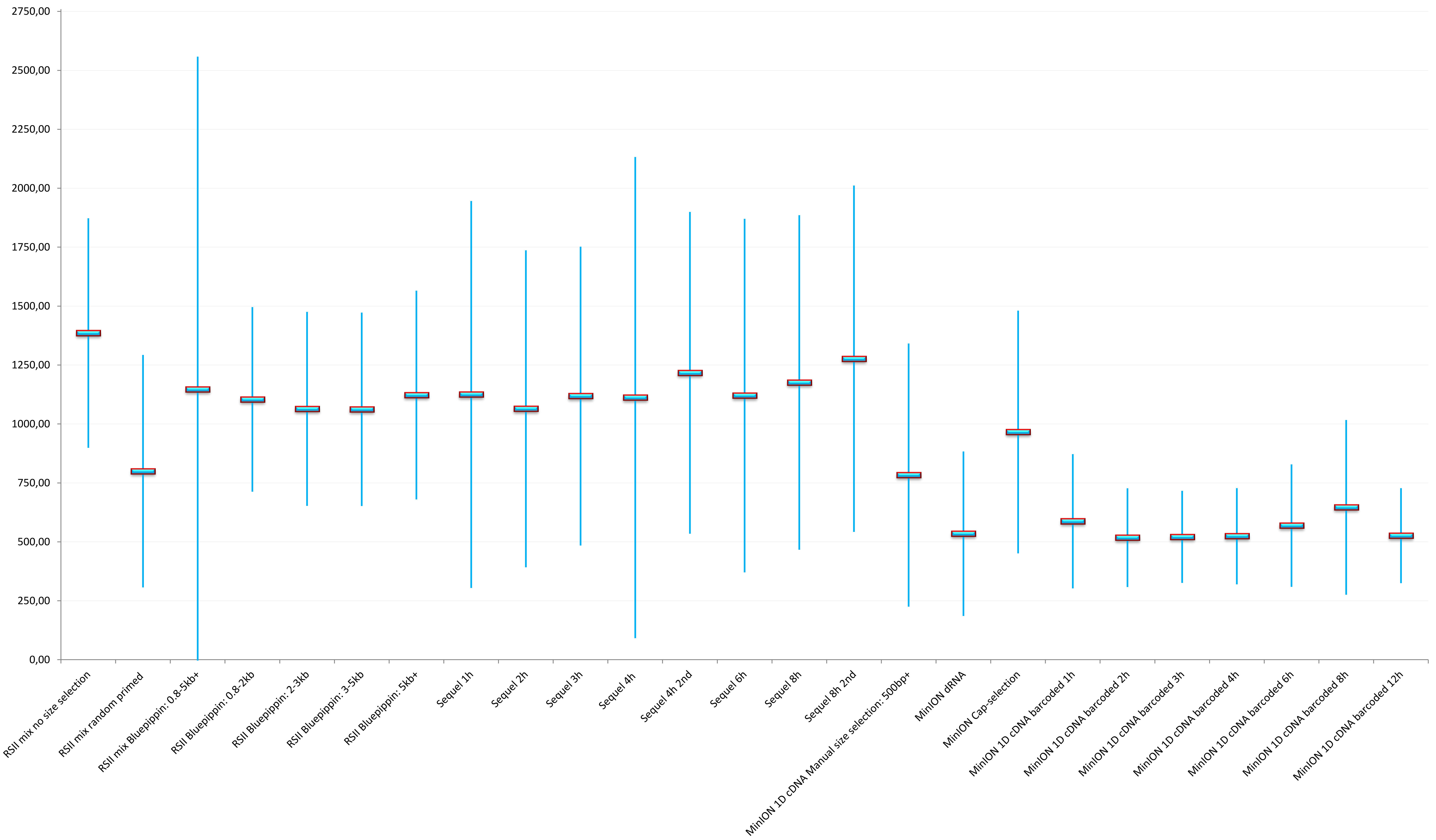

Figure 6

Click here to download Figure Fig6.pdf

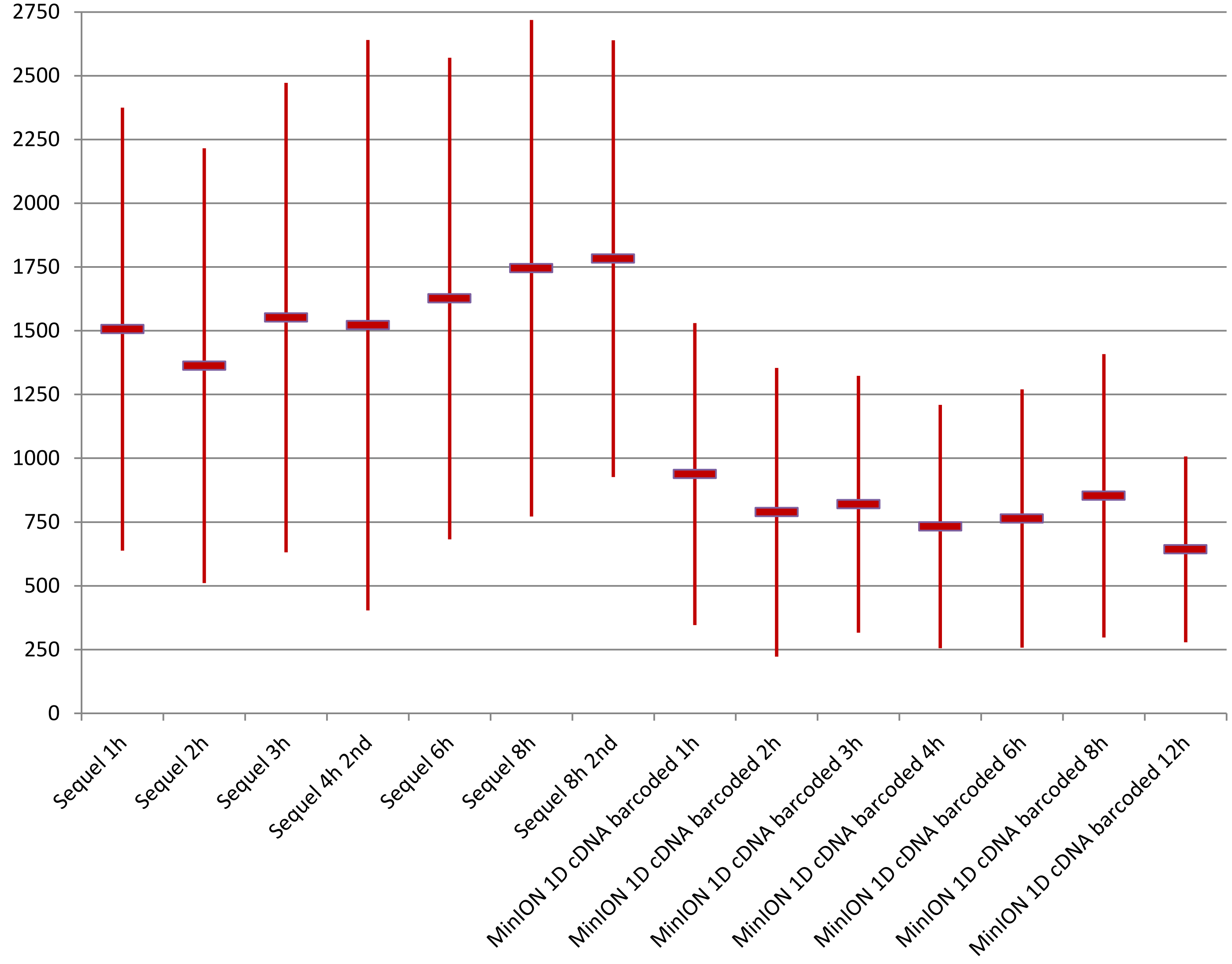

 Click here to download Figure Fig7.pdf

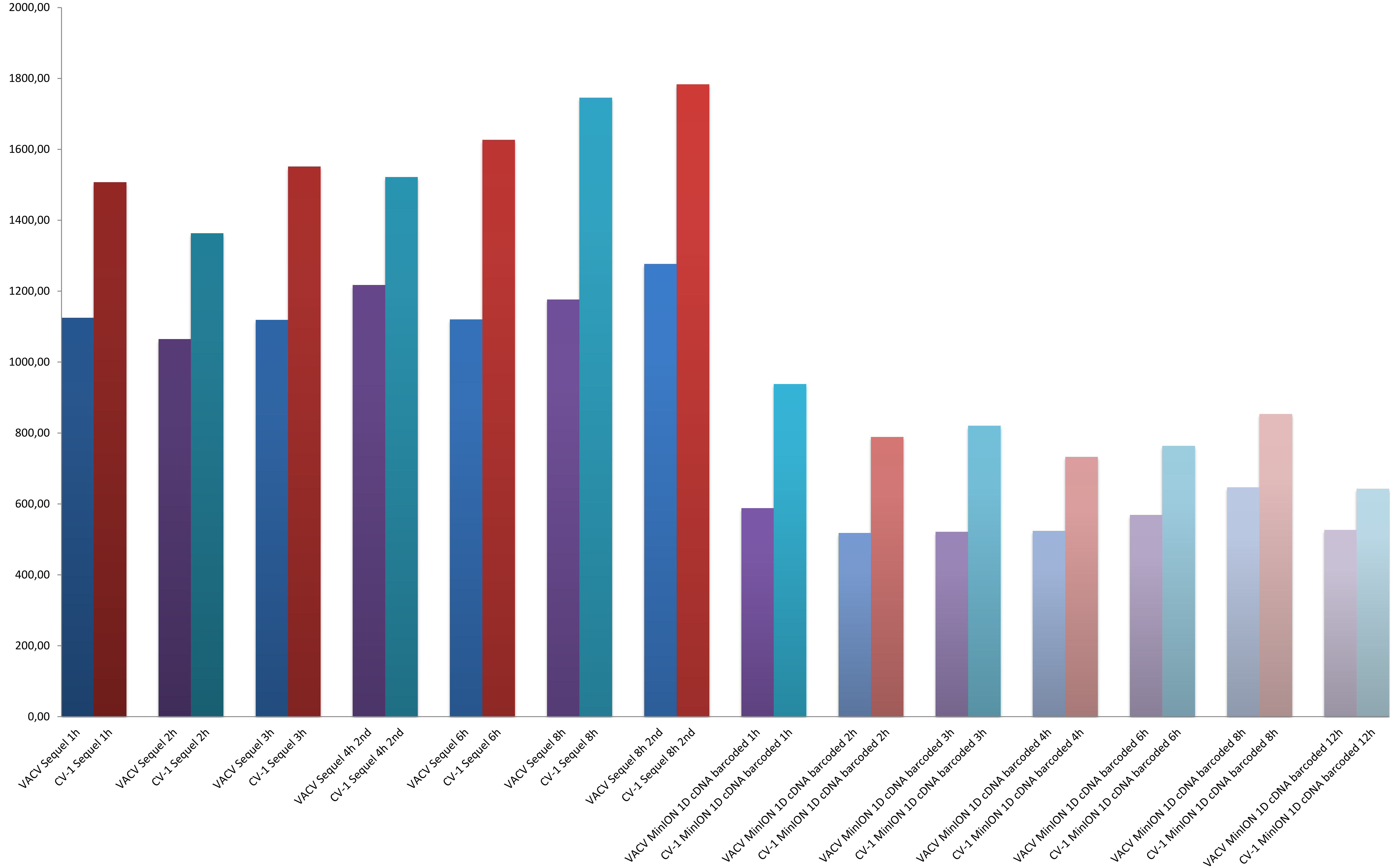

Supplementary Table 1

Click here to access/download
Supplementary Material
Additional_file1.xlsx

Supplementary Table 2

Click here to access/download
Supplementary Material
Additional_file2.xlsx

Supplementary Table 3

Click here to access/download
Supplementary Material
Additional_file3.xlsx
