# Supplementary material for: Dynamic transcriptome profiling dataset of vaccinia virus obtained from long-read sequencing techniques
Source: Gigascience. 2018 Nov 23;7(12):giy139. doi: 10.1093/gigascience/giy139 (PMC6290886; doi:10.1093/gigascience/giy139)
Supplement: giga-d-18-00175_revision_1.pdf [file giy139_giga-d-18-00175_revision_1.pdf]

GigaScience

Dynamic Transcriptome Profiling Dataset of Vaccinia Virus Obtained from Long-read Sequencing Techniques

--Manuscript Draft--

**Manuscript Number:**	GIGA-D-18-00175R1
**Full Title:**	Dynamic Transcriptome Profiling Dataset of Vaccinia Virus Obtained from Long-read Sequencing Techniques
**Article Type:**	Data Note
**Funding Information:**	Swiss-Hungarian Cooperation Programme (SH/7/2/8) — Prof. Dr. Zsolt Boldogkői<br>Magyar Tudományos Akadémia (Bolyai János Scholarship 2015-18) — Dr. Dóra Tombácz<br>NIH Centers of Excellence in Genomic Science (CEGS) Center for Personal Dynamic Regulomes (5P50HG00773502) — Prof. Dr. Michael Snyder<br>Nemzeti Kutatási, Fejlesztési és Innovációs Hivatal (OTKA K 128247) — Prof. Dr. Zsolt Boldogkői<br>Nemzeti Kutatási, Fejlesztési és Innovációs Hivatal (OTKA FK K 128252) — Dr. Dóra Tombácz<br>Tempus Public Foundation (HU) (Eötvös Scholarship of the Hungarian State) — Dr. Dóra Tombácz
**Abstract:**	Background Poxviruses are large DNA viruses infecting humans and animals. Vaccinia virus (VACV) has been applied as a live vaccine for immunization against smallpox, which was eradicated by 1980 as a result of worldwide vaccination. VACV is the prototype of poxviruses in the investigation of the molecular pathogenesis of the virus. Short-read sequencing methods have revolutionized transcriptomics; but, they are not efficient in distinguishing between the RNA isoforms and transcript overlaps. Long-read sequencing (LRS) is much better suited to solve these problems. Despite the scientific relevance of VACV, no LRS data have been generated for the viral transcriptome so far.<br><br>Findings For the deep characterization of the VACV RNA profile, various LRS platforms and library preparation approaches were applied. The raw reads were mapped to the VACV reference genome and also to the host (Chlorocebus sabaeus) genome. In this study, we applied the Pacific Biosciences RSII and Sequel platforms, which altogether resulted in 937,531 mapped reads of inserts (1.42 Gb), while we obtained 2,160,348 aligned reads (1.75 Gb) from the different library preparation methods, using the MinION device from Oxford Nanopore Technologies.<br><br>Conclusions By applying cutting-edge technologies, we were able to generate a large dataset that can serve as a valuable resource for the investigation of the dynamic VACV transcriptome, the virus-host interactions and the RNA base modifications. These data can provide useful information for novel gene annotations in the VACV genome. Our dataset can also be applied for analyzing the currently available LRS platforms, library preparation methods and bioinformatics pipelines.
**Corresponding Author:**	Zsolt Boldogkői<br>Szegedi Tudomanyegyetem Altalanos Orvostudomanyi Kar<br>Szeged, HUNGARY
**Corresponding Author Secondary Information:**	
**Corresponding Author's Institution:**	Szegedi Tudomanyegyetem Altalanos Orvostudomanyi Kar
**Corresponding Author's Secondary Institution:**	

Powered by Editorial Manager® and ProduXion Manager® from Aries Systems Corporation

**First Author:**	Dóra Tombácz
**First Author Secondary Information:**	
**Order of Authors:**	Dóra Tombácz
	István Prazsák
	Attila Szűcs
	Béla Dénes
	Michael Snyder
	Zsolt Boldogkői
**Order of Authors Secondary Information:**	
**Response to Reviewers:**	Dear Editor,<br><br>We would like to thank you and the three referees for the helpful comments and suggestions to our manuscript. We have made the recommended corrections and we hope that the manuscript will now be acceptable for publication in GigaScience. The changes have been marked in blue in the revised version of the manuscript. Our point-by-point responses to the comments are outlined below.<br><br>Reviewer #1:<br><br>Tombacz et al. used multiple long-read sequencing techniques to sequence VACV and its host cell transcripts during infection. This study obtained over one million reads. Full-length long deep-sequencing reads are critically needed in poxvirus research due to the complexity of poxvirus transcripts: pervasive transcription initiation, termination, extensive read-throughs, tightly spaced ORFs et al. Some of these issues could not be adequately addressed using sequencing technologies yielding relatively short reads. While these data likely provide useful dataset for poxvirus study, more information is required to access the quality of the data and its utilities.<br><br>Major points<br>1. Based on the information provided, it is not clear whether the reads (or what percentage of the reads) are full-length transcripts (although the authors claimed that they obtained full-length reads in line 91). Especially, the lengths from different sequencing platforms varied greatly. If there are only a very small portions of the reads are full-length transcripts, the advance of this study is limited as compared to those earlier studies.<br><br>We have added this information to the text of the revised version of the manuscript. Furthermore, a new figure (Figure 1) has also been added to the manuscript.<br><br>2. Read length distribution analysis, in addition to the average/median read lengths, will be informative to access the quality of the reads.<br><br>We have added this information to the text. Three new figures (Figures 9, 10 and 11) have been added to the manuscript. The read-length distributions for the dataset are shown in Figure 9 (reads mapped to the VACV genome), as well as Figures 10 and 11 (data aligned to the VACV and to the host genome).<br><br>3. Please discuss different read lengths using different sequencing platforms. They are unlikely all full-length transcripts. What are the advantages and disadvantages in poxvirus transcriptome analyses of the reads from different sequencing platforms?<br><br>We have added this information to the text of the revised version of the manuscript. The various sample preparation and sequencing techniques produce different read-length, read number and precision. Additionally, the various techniques produce different artifacts. There is a relatively large difference between the PacBio and ONT sequencing approaches concerning the quality of the sequencing reads: PacBio produces much fewer mismatches and INDELs than nanopore sequencing. However, high quality reads are unnecessary for transcriptome studies if the genome of the organism is known. The various sequencing platforms recommend different cDNA

production kits, which contain different enzymes and primers for the RT and PCR. The different primers and library preparation conditions may produce different artifacts; however, these can be easily filtered out if we compare the results of different methods. The PacBio MagBead loading selectively eliminates the short fragments (<1,000bp). While on the one hand, removal of incomplete cDNAs can be advantageous, at the same time, it is unfavorable, as we are unable to detect the shorter transcripts and RNA isoforms.

4. How is the average length of cellular transcripts in this study as compared to those in other studies?

A new figure (Figure 8) has been added to the manuscript. In this figure, we have compared the average aligned read-length of cellular transcripts obtained in this and other studies.

5. Specifically evaluate the reads (length, coverage, full-length or not, coverage in coding and non-coding regions, et al) of a few VACV ORFs and cellular genes with high resolution at different replication stages will help access the quality of the dataset.

A new table (Table 6) has been added to the manuscript. We have also added a heatmap illustration (Figure 12) on the expression dynamics of ten viral and ten host-specific genes.

6. What are the percentages of viral and cellular reads at each time point?

We have provided this information in the revised manuscript (see Figure 6).

7. Fig. 4. Visualization of reads coverage on VACV genome at individual time points is needed to access the quality of reads at different stages of VACV replication.

We have added an additional figure panel (Figure 5B) that presents the individual time points from Sequel and MinION sequencing. We have also retained the previous figure as Figure 5A, which includes the five different platforms and/or cDNA library preparation approaches. There are no individual time points from RSII and MinION Cap-Seq, and there is also an RSII dataset from mixed time points samples.

8. Sequencing error rates were not discussed in the manuscript.

We have added this information to the text.

Minor points.
1. Lines 49-50, is VACV a cowpox virus? From what I understand, it is not clear.

Yes, the VACV is the cowpox. We have modified the text for better clarification.

2. Table 1, what is Polya(A)(+) in the RNA sample column? Poly(A)(+)?

Yes, it is Poly(A)(+). We have corrected the misspellings.

3. Please correct some errors or typos throughout the manuscript. For example, line 116, PSB -PBS. Line 99, A detailed workflow-detailed workflows.

We have corrected the errors and typos.

4. Fig 5. 6. 7, Please label Y-axis.

Figure 5 and 6 have been changed to box plot and the Y-axis has been labeled, but Figure 7 has been removed as recommended by Reviewer #2.

Reviewer #2:

In the manuscript "Dynamic Transcriptome Profiling Dataset of Vaccinia Virus Obtained from Long-read Sequencing Techniques" by Tombácz et al., the authors describe a

dataset produced by Pacbio and Nanopore sequencing of VACV, with multiple approaches taken in both sample collection and sequencing library preparation in order to profile several features of the transcriptome. The dataset is of obvious importance to the field and contains many interesting features, but there are some concerns which would need to be addressed before this manuscript is ready for publication.
Specific points:
1. It would be beneficial to include a brief statement defining "dynamic" and "static" sample collection strategies in your data description to clarify how you are using these terms. It is unclear what infection condition means in the Table 1 context, and should either be clarified or replaced by something along the lines of "sample collection strategy".

We have defined the terms ‘static’ and ‘dynamic’ in the Table legend, and replaced ‘infection condition’ with ‘sample collection strategy’ as suggested.

2. How do the samples in Table 2 (A, B, C) correlate with the sequencing run in Table 1?

An additional column has been added to Table 2. Furthermore, details from RSII sequencing have also been added.

3. Why was yield so high for the viral genome given the lack of selection? Is the level of viral transcript typically this high, and what you would expect?

We have developed a novel program recently published on Github, which was used for the reanalysis of our data in the revised manuscript: https://github.com/Szunyike/SAM-Statistic-2018
The ratio of viral transcripts is 21.9% on average in our samples. The exact ratio is dependent on the titer of the virus used for the infection, as well as on the stage of the viral life cycle at the examination period. The sequencing method affects the ratio of read counts between the virus and host cell: e.g. the MinION 1D-Seq method yields a higher amount of shorter reads compared to the PacBio Sequel technique. The VACV transcripts are relatively short compared to the host or to other large DNA viruses (such as herpesviruses and baculoviruses), which is assumed to result in the relatively high ratio of viral reads compared to the host reads in the MinION samples.

4. Tables 6 and 7 should be combined for ease of interpretation. Tables are not as useful to show read/insert length distribution as actually plotting the distribution, and box and whiskers are inadequate. We recommend pulling out error rates, and representing instead as violin plots, or at least box plots, and include a column for "total reads" and either the count of mapped VACV reads, or percent reads mapped to VACV. Similarly, figures 5 and 6 should be combined to allow for direct comparison, and figure 7 should be removed.

Tables 6 and 7 have been combined into a single table (Table 5). Figures 5 and 6 have been changed to a box plot and they are combined into a single figure (Figure 7). Read-length distribution has also been presented in the revised manuscript (Figures 9, 10, 11 and Additional file 3). Figure 7 of the old version of the manuscript has been removed. We have provided a new figure (Figure 6) showing the percentages of viral and cellular reads at each sample.

5. Regarding alignments, it is unclear why certain sequencing were aligned to VACV and why others were aligned to both VACV and CV-1. For example, why aren't the RSII and direct RNA runs included in Table 7 and Figure 6 host mappings?

We have aligned the RSII and dRNA data to the CV-1 cell line and the statistical data has been added to Table 5 (Table 7 in the old version).

6. On your Circos plot, it would be interesting to color genes by early/intermediate/late stage. Also regarding the Circos plot - where is the dRNA data?

The Circos plot has been modified according to the recommendation: the various kinetic classes have been labeled in different colors (Figure 5A). Another picture of coverage on VACV genome at individual time points has also been added (Figure 5B)

Powered by Editorial Manager® and ProduXion Manager® from Aries Systems Corporation

as was suggested by another Reviewer. The coverage in dRNA sequencing is very low compared to the other five (Sequel, RSII, MinION 1D cDNA, MinION 1D barcoded, and MinION Cap) approaches, and it is not visible at the scale applied for the generation of circus plot. Therefore, we have added a Shashimi plot as a part of this figure (Figure 5C) instead of adding the dRNA values to the same circus plot.

7. In Table 6, your coverage calculations seem improbable in places. For example, how could the RSII 5kb+ BluePippin run with 8 mapped reads have 42.17X coverage?

We have corrected this mistake.

8. Your statement about searching for modifications in dRNA should be amended - although 5mC is widespread in DNA, it is not as ubiquitous in RNA. Additionally, 6mA has been most extensively profiled in viruses: http://jvi.asm.org/content/91/9/e02263-16.full, and is actually quite common.

We have corrected this part of the manuscript.

9. In the 1D ONT-cDNA sequencing section, instead of telling the reader that different steps were used from the ONT protocol, I suggest describing what enzymes were used for the end-polishing. The ONT protocols are not publicly accessible, and since the preparations done on these samples were so complex, it may make sense to just say what was done.

We have added detailed information about the ONT 1D cDNA and direct RNA protocols to the revised manuscript.

10. Why not minimap2 instead of GMAP? Choice of GMAP is perfectly acceptable, but for readers it would help to explain the choice.

We have chosen GMAP because we have found it the best long-read aligner in our earlier publications [18, 19, 20, 21, 22, 23, 24]. GMAP have also produced the best alignment results in other studies [e.g. 28]. We generated the PacBio RSII dataset in 2016, when Minimap2 had not yet existed. In the beginning, Minimap2 did not support for RNA mapping (version 1: https://arxiv.org/abs/1708.01492v1). Our MinION and Sequel data are newer, and later versions of the Minimap2 program now support RNA mapping. However, our opinion is that the correct approach would be to use the same program for mapping that we had used to attain our existing data. We have added the Minimap2 program as a recommended long-read aligner to the ‘Conclusion and Reuse Potential’ section of the manuscript.

11. Language occasionally awkward, for example "VACV remains to be considered a weapon against potential smallpox outbreaks". Careful editing for grammar, subject-verb agreement will greatly improve readability.

The manuscript has been thoroughly edited in terms of grammar, and was proofread by a native speaker of English.

Some minor points:
1. Fastqs (unsorted by organism, just of all reads, for each run) should be hosted on gigascience for ease of access - this would be the first piece of information we would want to download.

We have uploaded the fastq.qz files to FigShare:
https://figshare.com/s/675f5f71c633473b7445

2. In findings section of the abstract, it is more useful to include the genus/species of host, and leave the accession numbers to the methods section. Additionally, it would be more useful to include Gb data produced, as well as the size of the genome, upfront.

We have modified the text as recommended.

3. In table 1, please make yes/no capitalization consistent. A label of some kind linking

the workflows in Fig 1 and Fig 2 and 3 - or linking to the Table would be very helpful. Many different preparations are represented here, and parsing them in the current format is challenging at best. Perhaps some sort of encoding like ONT-cDNA-polyA_1 at the bottom of fig 3 for the relevant workflow and labeled as a column in Table 1? Additionally, please make clear for fig 3 what is dRNA and what is cDNA.

The recommended corrections have been made in Table 1. We have also made some minor modifications on Figures 3 and 4 (Figures 2 and 3 in the old version of the MS) according to the reviewer's suggestion. Abbreviations of dRNA and cDNA are now explained and we have provided a linking label between Figures 2, 3 and 4 (Figure 1, 2 and 3 in the old version of the manuscript). The labels are as follows: „Workflow A, B, C, D, E, F, G and H", and can clearly identify the different methods depicted on Figures 3 and 4 (Figures 2 and 3 in the old version of the MS).

4. Sometimes you denote 5'/3', sometimes 5′/3′, please make consistent to 5′/3′.

We have modified the text with consistent labeling of 5'/3' throughout the manuscript.

5. All of your figures should have y-axis labels and, in the case of figures 4 and 7 - a legend explaining the colors.

The rectangles are colored according to the known kinetic properties of the genes: red: early 1; green: early 2 (early-late; and yellow: postreplicative (late). Y-axis labels have been added to the figures. Figure 7 has been removed as recommended by Reviewer #2

6. Please add "availability of source code and requirements" as per https://academic.oup.com/gigascience/pages/data_note

We have added the required information to the manuscript.

7. Standard deviance should be changed to standard deviation.

We have corrected the wording as recommended.

8. The statement in line 235 "These aligned reads can be further analyzed by using different long read aligners" should be modified to "These aligned reads can be further analyzed by comparing to results of different long read aligners"

This sentence has been changed as recommended.

9. Alignment file names should be updated to a consistent format

A supplementary table (Additional file 4) has been added which explains the file names of alignments deposited in ENA paired with the names that are used in this manuscript.

10. Tables 3 and 5 should be combined.

These two tables have been combined. Additional data have also been added.

11. Table 1 and Figure 1 are redundant, consider removing Figure 1 or adding additional unique information to it.

We have provided additional information to Figure 2 (Figure 1 in the old version of the MS) according to the reviewer's recommendation: we have labeled the unique workflows (A, B, C, D, E, F, G and H) on Figure 2, which helps to identify the different methods presented in the Figures 3 and 4 (Figures 2 and 3 in the old version of the manuscript).

12. Your text mentions a "fast5.tar.gz" file for ONT dRNA, but listed in the dRNA folder of archives is just a "tar.gz" file in addition to the BAMs - please clarify that this is the same. Also, you only included the raw reads which aligned to VCAV - but it is worthwhile to include all reads as well, because your alignments might not be all-inclusive.

Powered by Editorial Manager® and ProduXion Manager® from Aries Systems Corporation

The files Fast5.tar.gz and tar.gz files are the same. The reads aligned to the host genome have been deposited in the ENA.

13. What size is the tissue culture flask for the CV-1s?

We used 25 cm2 culture flasks for the propagation of CV-1 cells. This information has been added to the MS.

14. Why were 3x freeze-thaw cycles applied? What is the reasoning for that?

Freezing and thawing were carried out to help the degradation of cell membrane and nuclear envelope for more efficient isolation of RNA molecules.

Third report:
In this Data Note, the authors present full-length transcriptome data generated by third-generation single-molecule long-read sequencing technologies including PacBio (Pacific Biosciences) and ONT (Oxford Nanopore Technologies) for VACV (Vaccinia virus). Two full-length cDNA synthesis methods (Clontech SMARTer and Lexogen TeloPrime) and three platforms (PacBio RS II, PacBio Sequel and ONT MinION) were applied in this study. The authors also generated transcriptome data using ONT direct RNA sequencing technology. These data will benefit the identification of novel VACV RNA isoforms for virologists and the development and evaluation of bioinformatics tools. Below are some comments on this manuscript.

1. The authors used two strategies ("no size selection" and "Bluepippin Size Selection") for PacBio RS II platform. Why the number of ROIs generated by "no size selection" is significantly smaller than "Bluepippin Size Selection" (Table 6)?

The size-selected samples do not necessarily produce higher yields than the non-size-selected samples. In some cases, PacBio run results in low output, for example because of underloading of the SMRT Cell. This sample is a minor part of our dataset. We have labeled this sample with an asterisk (*) in Table 5 (Table 6 in the previous version of the MS) and indicated the possible cause for the yield differences in the figure legend.

2. In Tables 6 and 7, three columns "insertion frequency", "deletion frequency" and "mismatch frequency" are shown, but the authors did not describe them in "Data summary" section. More details should be included.

We have added more details to the Data summary section of the revised manuscript.

3. The grammar should be improved. For example, in Page 4 Line 81, "determine which transcripts contains certain 5'-ends and 3'-ends."; in Page 4 Line 86, "By using these techniques for cDNA production and"; Page 7 Line 140, "240 minutes were set for the RSII movie lengths, while 600 min were applied"; in Page 10 Line 191-192, "This basecaller is able identify the nucleotide sequences directly from raw sequencing data."

We have corrected the grammar.

4. In Table 2, the comma should be converted to the dot.

We have corrected this error.

5. In Page 6 Line 42-44, to the best of our knowledge, m6A is an abundant modification in mRNA. Could the authors check whether m6A or 5mC is most widespread in RNA modifications? In addition, the abbreviation should be "m6A" for RNA N6-Methyladenosine. The "6mA" is the abbreviation of DNA N6-methyladenine.

We have modified the appropriate part of the manuscript. We also have added new text and new references to the revised MS. Furthermore, we have changed the abbreviation of N6-Methyladenosine to “m6A”.

Powered by Editorial Manager® and ProduXion Manager® from Aries Systems Corporation

	6. In Page 15 Line 293, the full name of "TSS" should be "transcription start site".<br><br>We have corrected this error.<br><br>https://giga.editorialmanager.com/l.asp?i=41827&l=SE8GCNWN
**Additional Information:**	
**Question**	**Response**
Are you submitting this manuscript to a special series or article collection?	No
**Experimental design and statistics**<br><br>Full details of the experimental design and statistical methods used should be given in the Methods section, as detailed in our Minimum Standards Reporting Checklist. Information essential to interpreting the data presented should be made available in the figure legends.<br><br>Have you included all the information requested in your manuscript?	Yes
**Resources**<br><br>A description of all resources used, including antibodies, cell lines, animals and software tools, with enough information to allow them to be uniquely identified, should be included in the Methods section. Authors are strongly encouraged to cite Research Resource Identifiers (RRIDs) for antibodies, model organisms and tools, where possible.<br><br>Have you included the information requested as detailed in our Minimum Standards Reporting Checklist?	Yes
**Availability of data and materials**<br><br>All datasets and code on which the conclusions of the paper rely must be either included in your submission or deposited in publicly available repositories (where available and ethically appropriate), referencing such data using	Yes

Powered by Editorial Manager® and ProduXion Manager® from Aries Systems Corporation

a unique identifier in the references and in the “Availability of Data and Materials” section of your manuscript.<br><br>Have you have met the above requirement as detailed in our Minimum Standards Reporting Checklist?	

Manuscript
Click here to access/download;Manuscript;GigaScience-Tombacz.docx
Click here to view linked References

Dynamic Transcriptome Profiling Dataset of Vaccinia Virus Obtained from Long-read Sequencing Techniques

Dóra Tombácz[1], István Prazsák[1], Attila Szűcs[1], Béla Dénes[2], Michael Snyder[3], Zsolt Boldogkői[1*]

[1]Department of Medical Biology, Faculty of Medicine, University of Szeged, Somogyi B. u. 4., 6720 Szeged, Hungary

[2]Veterinary Diagnostic Directorate of the National Food Chain Safety Office, Keleti Károly u. 24., 1024 Budapest, Hungary

[3]Department of Genetics, School of Medicine, Stanford University, 300 Pasteur Dr, Stanford, California, USA

E-mails:

DT: tombacz.dora@med.u-szeged.hu

IP: prazsak.istvan@med.u-szeged.hu

AS: szucs.attila.1@med.u-szeged.hu

BD: denesb@nebih.gov.hu

MS: mpsnyder@stanford.edu

ZB: boldogkoi.zsolt@med.u-szeged.hu

*Corresponding author: ZB

Abstract

Background Poxviruses are large DNA viruses infecting humans and animals. Vaccinia virus (VACV) has been applied as a live vaccine for immunization against smallpox, which was eradicated by 1980 as a result of worldwide vaccination. VACV is the prototype of poxviruses in the investigation of the molecular pathogenesis of the virus. Short-read sequencing methods have revolutionized transcriptomics; but, they are not efficient in distinguishing between the RNA isoforms and transcript overlaps. Long-read sequencing (LRS) is much better suited to solve these problems. Despite the scientific relevance of VACV, no LRS data have been generated for the viral transcriptome so far.

Findings For the deep characterization of the VACV RNA profile, various LRS platforms and library preparation approaches were applied. The raw reads were mapped to the VACV reference genome and also to the host (*Chlorocebus sabaeus*) genome. In this study, we applied the Pacific Biosciences RSII and Sequel platforms, which altogether resulted in 937,531 mapped reads of inserts (1.42 Gb), while we obtained 2,160,348 aligned reads (1.75 Gb) from the different library preparation methods, using the MinION device from Oxford Nanopore Technologies.

Conclusions By applying cutting-edge technologies, we were able to generate a large dataset that can serve as a valuable resource for the investigation of the dynamic VACV transcriptome, the virus-host interactions and the RNA base modifications. These data can provide useful information for novel gene annotations in the VACV genome. Our dataset can also be applied for analyzing the currently available LRS platforms, library preparation methods and bioinformatics pipelines.

Keywords: poxvirus, vaccinia virus, long-read sequencing, full-length transcriptome, Pacific Biosciences, RS II system, Sequel system, Oxford Nanopore Technologies, MinION system, direct RNA sequencing

Data Description

Background

Poxviridae is a large virus family that infects vertebrates and invertebrates with highly pathogenic members, such as the Variola virus, which is the causative agent of smallpox [1]. Vaccinia virus (VACV; cowpox virus) is the prototypic member of the Orthopoxvirus genus [2]. It is closely related to the *Variola viruses* [3] that was eradicated as a result of successful global vaccination program using live VACV. Vaccinia virus has been extensively utilized as an expression and a gene delivery vector [4]. It also serves as a model system for the analysis of virus-host interactions, transcriptional regulation, and for other molecular biological studies [5].

Poxviruses are able to replicate in the cytoplasm of the host cell because they encode the proteins needed for DNA synthesis [6]. They have a relatively large (approximately 195 kbp) double-stranded DNA genomes coding for about 220 proteins. The VACV genes are divided into three temporal classes: early (E), intermediate (I) and late (L) genes. A study characterized 35 VACV genes as immediate-early (IE) kinetics [7], but this categorization has not been widely accepted. The promoters of genes belonging to different kinetic classes are recognized by stage-specific transcription factors [8, 9, 10, 11]. VACV genes belonging to the same kinetic group have been shown to be clustered in the genome [7]: E genes are located at the termini of the viral genome, while I and L genes are situated in the middle genomic region. Most of the adjacent VACV genes are oriented in the same direction, while convergent and divergent positionings are uncommon.

Although the extraordinary complexity of the VACV transcriptome has been thought to be well-characterized [12, 13, 14, 15], traditionally used techniques such as short-read sequencing (SRS), ribosome profiling, cap analysis of gene expression (CAGE), genome tiling [16] are not able to span the entire transcript, nor to distinguish between transcript isoforms, bi-, and polycistronic RNA variants, overlapping gene products and embedded RNAs. Transcriptional overlaps generated by the

read-through mechanism are very frequent in VACV and cause a major problem in the analysis of individual viral transcripts using traditional approaches. The transcription patterns of VACV genes exhibit an extreme stochasticity, which includes an enormous number of transcriptional start sites (TSSs) and transcription end sites (TESs) even within the open reading frames (ORFs). These features of transcription are uncommon even among large DNA viruses; it might represent a form of gene regulation that is unique to living organisms. Therefore, it is especially important to use full-length sequencing methods in order to match the transcript ends.

Previous studies have already determined the precise TSSs and TESs of VACV transcripts [14, 17], but the methods that were applied were not suitable for detecting the entire transcripts at single-molecule level, and therefore it was impossible to determine which TSSs are paired by certain TESs.

The Pacific Biosciences (PacBio) isoform sequencing (Iso-Seq) protocol (using oligo(d)T or random hexamer primers for the reverse transcription), the cDNA sequencing and direct (d)RNA sequencing methods from the Oxford Nanopore Technologies (ONT), as well as the Cap-selection (Cap-Seq) cDNA preparation method (Lexogen) are able to generate full-length transcripts, and thus they can circumvent the limitations of SRS techniques. By using these techniques for cDNA productions and library preparations with the PacBio Real-Time Sequencer (RS)II and Sequel, as well as the ONT MinION platforms, we were able to identify hundreds of novel RNA isoforms (e.g. TSS and TES variants, mono-, bi-, polycistronic transcripts), dozens of coding and non-coding RNAs, and numerous complex transcripts in various herpesviruses [18, 19, 20, 21, 22, 23] and in a baculovirus [24], and we were also able to generate a comprehensive full-length transcript data catalog of VACV. The PacBio Sequel and the ONT Cap-Seq methods yielded the highest amount of full-lengths reads in our experiments (**Figure 1**). The ratio between the complete and partial reads varies within the size-selected RSII samples. ONT 1D cDNA sequencing yielded the lowest ratio of full-length transcripts. However, as these samples yielded the highest number of read counts, full-length transcripts are also present in a large number. Even if a large proportion of the reads are incomplete,

they can be utilized for e.g. distinguishing between the different transcript isoforms, or for identifying overlapping transcripts. The latter is essential for the kinetic classification of the overlapping transcripts: for example, if an early transcript overlaps a late transcript, the latter one could be miscategorized as early [13]. A large number of incomplete reads have been obtained from the dRNA-Seq, which were consistent with our previous results [24]. The current method of this technique produces sequencing reads missing varying size short sequences from both ends. Random-primed RT-based sequencing rarely gains complete reads, the reason for which is that the primers seldom bind to exactly the 3'-ends of the transcripts. However, these samples provide further significant value to the dataset: for example, random-primed sequencing may result in novel, non-polyadenylated transcripts [25, 26], while direct RNA sequencing data may provide epitranscriptomic information by detecting base modifications (e. g.: m7G). Furthermore, the dRNA-Seq method is free of artifacts produced by RT and PCR in cDNA sequencing.

The present report provides the first long-read, dynamic RNA profiling dataset from the family of Poxviruses and the host cell line (CV-1), which can redefine the VACV transcriptomic landscape. This study is a very large cohort of data from the currently available third-generation sequencing methods representing the forefront techniques for transcriptome research. As such, the data presented herein can provide to be useful not only at the molecular level and not just for virologists, but also with respect to general genomics and bioinformatics.

Methods

A detailed workflow pertaining to the different library preparation strategies is presented in **Figure 2, 3, 4 and Table 1**.

Table 1.

Run #	Platform	Sample collection strategy	Time Points (h)	RNA sample	RT priming	Cap-selection	PCR	Size selection	Library prep	Barcodes	Base calling

1	RSII	Static	1, 2, 4, 8	PolyA(+)	Oligo(d)T	No	Yes	No	Iso-Seq	No	SMRT Analysis v2.3.0
2	RSII	Static	1, 2, 4, 8	rRNA depletion	Random hexamer	No	Yes	No	Iso-Seq	No	SMRT Analysis v2.3.0
3	RSII	Static	1, 2, 4, 8	PolyA(+)	Oligo(d)T	No	Yes	BluePippin 0.8kb-5kb+	Iso-Seq	No	SMRT Analysis v2.3.0
4	RSII	Static	1, 2, 4, 8	PolyA(+)	Oligo(d)T	No	Yes	BluePippin 0.8-2kb	Iso-Seq	No	SMRT Analysis v2.3.0
5	RSII	Static	1, 2, 4, 8	PolyA(+)	Oligo(d)T	No	Yes	BluePippin 2-3kb	Iso-Seq	No	SMRT Analysis v2.3.0
6	RSII	Static	1, 2, 4, 8	PolyA(+)	Oligo(d)T	No	Yes	BluePippin 3-5kb	Iso-Seq	No	SMRT Analysis v2.3.0
7	RSII	Static	1, 2, 4, 8	PolyA(+)	Oligo(d)T	No	Yes	BluePippin 5kb+	Iso-Seq	No	SMRT Analysis v2.3.0
8	Sequel	Dynamic	1	PolyA(+)	Oligo(d)T	No	Yes	No	Iso-Seq	No	SMRT Link v5.0.1.9585
9	Sequel	Dynamic	2	PolyA(+)	Oligo(d)T	No	Yes	No	Iso-Seq	No	SMRT Link v5.0.1.9585
10	Sequel	Dynamic	3	PolyA(+)	Oligo(d)T	No	Yes	No	Iso-Seq	No	SMRT Link v5.0.1.9585
11	Sequel	Dynamic	4	PolyA(+)	Oligo(d)T	No	Yes	No	Iso-Seq	No	SMRT Link v5.0.1.9585
12	Sequel	Dynamic	4	PolyA(+)	Oligo(d)T	No	Yes	No	Iso-Seq	No	SMRT Link v5.0.1.9585
13	Sequel	Dynamic	6	PolyA(+)	Oligo(d)T	No	Yes	No	Iso-Seq	No	SMRT Link v5.0.1.9585
14	Sequel	Dynamic	8	PolyA(+)	Oligo(d)T	No	Yes	No	Iso-Seq	No	SMRT Link v5.0.1.9585
15	Sequel	Dynamic	8	PolyA(+)	Oligo(d)T	No	Yes	No	Iso-Seq	No	SMRT Link v5.0.1.9585
16	MinION	Static	1, 2, 3, 4, 6, 8, 12, 16	PolyA(+)	Oligo(d)T	No	Yes	Manual Gel 500bp+	1D cDNA	No	Albacore v.2.0.1
17	MinION	Static	1, 2, 3, 4, 6, 8, 12, 16	Total RNA	Oligo(d)T	Yes	Yes	No	Teloprime + 1D	No	Albacore v.2.0.1
18	MinION	Static	1, 2, 3, 4, 6, 8, 12, 16	PolyA(+)	Oligo(d)T	No	No	No	dRNA	No	Albacore v.2.0.1
19	MinION	Dynamic	1	PolyA(+)	Oligo(d)T	No	Yes	No	1D cDNA	Yes	Albacore v.2.0.1
20	MinION	Dynamic	2	PolyA(+)	Oligo(d)T	No	Yes	No	1D cDNA	Yes	Albacore v.2.0.1
21	MinION	Dynamic	3	PolyA(+)	Oligo(d)T	No	Yes	No	1D cDNA	Yes	Albacore v.2.0.1
22	MinION	Dynamic	4	PolyA(+)	Oligo(d)T	No	Yes	No	1D cDNA	Yes	Albacore v.2.0.1
23	MinION	Dynamic	6	PolyA(+)	Oligo(d)T	No	Yes	No	1D cDNA	Yes	Albacore v.2.0.1
24	MinION	Dynamic	8	PolyA(+)	Oligo(d)T	No	Yes	No	1D cDNA	Yes	Albacore v.2.0.1
25	MinION	Dynamic	12	PolyA(+)	Oligo(d)T	No	Yes	No	1D cDNA	Yes	Albacore v.2.0.1

Cells and viruses African green monkey (*Chlorocebus sabaeus*) kidney fibroblast cells [CV-1; American Type Culture Collection (ATCC)] were cultured in RPMI 1640 medium (Sigma-Aldrich) supplemented with 10% fetal bovine serum (FBS) and antibiotic-antimycotic solution (Sigma-Aldrich) in a 25 cm^2 culture flask at 37°C in a humidified 5% CO_2 atmosphere, until confluence was reached. The cells (~2.6 × 10^7) were washed with serum-free medium before the infection. The highly virulent Western Reserve (WR) VACV strain was used this study. The virus stock was diluted in serum-free RPMI 1640 medium, and then it was used (3 ml of VACV at 10 MOI/cell) for the CV-1 infection. Samples were incubated at 37°C in 5% CO_2 atmosphere for 1 h with brief agitation at 10 min intervals to redistribute the virus. Three milliliters of complete growth medium (RPMI 1640 +

10% FBS) was added to the tissue culture flask and the infected cells were further incubated for 1, 2, 4 and 8 hours for RSII sequencing, 1, 2, 3, 4, 6 and 8 hours for Sequel, or 1, 2, 3, 4, 6, 8, 12 and 16 hours for MinION sequencing (**Table 1**) at 37°C in a humidified 5% CO_2 atmosphere. After the incubation the cells were rinsed with serum-free RPMI 1640 medium, which was followed by the application of three freeze-thaw cycles. Cells were scraped into 2ml of phosphate-buffered saline (PBS), and stored at -80°C until use.

RNA Total RNA was purified from the infected cells at various stages of viral infection from 1 to 16h post-infection (pi) using an RNA Kit from Macherey-Nagel. Polyadenylated RNAs were purified from the cells by using the Oligotex mRNA Mini Kit (Qiagen, **Additional file 1**). For the analysis of non-polyadenylated RNAs, ribodepletion (Epicentre Ribo-Zero™ Magnetic Kit H/M/R) was carried out on the total RNA samples. RNAs were quantified (**Table 2**) by Qubit 2.0 using the Qubit RNA BR Assay Kit for the total RNAs and the Qubit RNA HS Assay Kit for the polyA(+) RNAs (Life Technologies). The quality of the samples was assessed with an Agilent 2100 Bioanalyzer. The samples used had RNA Integrity Numbers greater than 9.5.

Table 2.

Run #	Time points (h)	A (ng)	B (ng/µl)	C (ng/µl)
1	mixed	16.2	86.1	5.8
2	mixed	1000 *	109.1	5.2
3	mixed	14.6	75.5	6.9
4	mixed	14.6	98.2	7.2
5	mixed	14.6	84.1	7.1
6	mixed	14.6	89.4	9.1
7	mixed	14.6	77.9	7.9
8	1	27.3	410.0	8.1
9	2	10.0	112.0	9.0
10	3	18.9	87.8	11.1
11	4	51.8	460.0	12.1
12	4	19.9	98.3	6.8
13	6	20.3	95.0	7.8
14	8	39.2	460.0	12.0
15	8	19.6	120.0	6.1

Library preparation for PacBio RSII & Sequel sequencing The cDNAs were generated from the polyA(+) RNA fractions in accordance with PacBio's recommendations for isoform sequencing (Iso-Seq) method using the Clontech SMARTer PCR cDNA Synthesis Kit and No Size Selection' or the 'BluePippin size-selection' protocol (**Figure 2, 3, Table 1**). The samples collected at various time points (1, 4, 8 and 12h pi) were mixed together for the RSII sequencing; however, different time points (1, 2, 3, 4, 6, and 8h pi) were used individually for the production of cDNA libraries for the Sequel method. An rRNA-depleted sample mixture (1, 4, 8 and 12h) was converted to cDNA with modified random hexamer primers (**Table 3**) instead of the SMARTer Kit's oligo(d)T-containing oligo. The detailed library preparation methods are described in our recent publication [23]. Briefly, SMRTbell Template Prep Kit 1.0 was used for SMRTbell library production, followed by primer annealing using the DNA Sequencing Reagent Kit 4.0 v2 and polymerase (DNA Polymerase P6) binding for RSII sequencing, whereas the Sequel Sequencing Kit 2.1 and Sequel DNA Polymerase 2.0 were applied for the Sequel platform. Samples were bound to magbeads (MagBead Kit v2) for loading onto the PacBio instruments. The RSII movie lengths were set for 240 minutes, while 600-minutes movies were captured using the Sequel technique. A single movie was recorded for each SMRT Cell. Seventeen RSII SMRT Cells v3 and 8 Sequel SMRT Cells v2 (SMRT Cell 1M) were used for sequencing. The cDNA samples and the SMRTbell templates were quantified (**Table 2**) by Qubit using Qubit dsDNA HS (High Sensitivity) Assay Kit.

Table 3.

Sequencing method	Library prep step	Name, availability	Catalog #	Sequence (5' -> 3')
PacBio amplified PolyA	RT	3' SMART CDS primer II A - SMARTer PCR cDNA Synthesis Kit (Clontech)	634925 & 634926	AAGCAGTGGTATCAACGCAGAGTAC$(T)_{30}$VN
PacBio amplified Random	RT	Custom-made (IDT DNA)	-	AAGCAGTGGTATCAACGCAGAGTACNNNNNN (G: 37%; C: 37%; A: 13%; T: 13%)
MinION cDNA	RT	PolyT-containing anchored primer [(VN)T20 - ONT recommended, custom-made (Bio Basic)	-	5phos/ ACTTGCCTGTCGCTCTATCTTC$(T)_{20}$VN
MinION CAP-Seq	RT	TeloPrime Full-Length cDNA Amplification Kit (Lexogen)	013.08 & 013.24	TCTCAGGCGTTTTTTTTTTTTTTTTTT
MinION dRNA	RT	RT adapter - Direct RNA Sequencing Kit (Oxford Nanopore Technologies)	SQK-RNA001	GAGGCGAGCGGTCAATTTTCCTAAGAGCAAGAAGAAGCCTTTTTTTTTT
MinION CAP-Seq	test qPCR	D1R fw – custom-made (IDT DNA)	-	CGAACTAGAGGACCGTTGGG
MinION CAP-	test qPCR	D1R rev – custom-made (IDT DNA)	-	TTTCCAGGTCAGCACCGTTT

Seq				
MinION cDNA barcoded	barcoding	A1 />BC01/ (ONT PCR Barcoding Kit 96)	EXP-PBC096	AAGAAAGTTGTCGGTGTCTTTGTG
MinION cDNA barcoded	barcoding	A2 />BC02/ (ONT PCR Barcoding Kit 96)	EXP-PBC096	TCGATTCCGTTTGTAGTCGTCTGT
MinION cDNA barcoded	barcoding	A3 />BC03/ (ONT PCR Barcoding Kit 96)	EXP-PBC096	GAGTCTTGTGTCCCAGTTACCAGG
MinION cDNA barcoded	barcoding	A4 />BC04/ (ONT PCR Barcoding Kit 96)	EXP-PBC096	TTCGGATTCTATCGTGTTTCCCTA
MinION cDNA barcoded	barcoding	A5 />BC05/ (ONT PCR Barcoding Kit 96)	EXP-PBC096	CTTGTCCAGGGTTTGTGTAACCTT
MinION cDNA barcoded	barcoding	A6 />BC06/ (ONT PCR Barcoding Kit 96)	EXP-PBC096	TTCTCGCAAAGGCAGAAAGTAGTC
MinION cDNA barcoded	barcoding	A7 />BC07/ (ONT PCR Barcoding Kit 96)	EXP-PBC096	GTGTTACCGTGGGAATGAATCCTT
PacBio	adapter ligation	PacBio blunt adapter (PacBio Template Prep Kit 1.0)	PN 100-222-300	ATCTCTCTCTTTTCCTCCTCCTCCGTTGTTGTTGTTGAGAGAGAT
MinION	adapter ligation	5' adapter (ONT Ligation Sequencing 1D kit)	SQK-LSK108	GGTGCTG
MinION	adapter ligation	3' adapter (ONT Ligation Sequencing 1D kit)	SQK-LSK108	TTAACCT

ONT MinION cDNA sequencing The polyA(+) RNAs were used for cDNA sequencing on the MinION device. We prepared one library from the RNA mixture (RNA samples from the 1, 2, 3, 4, 6, 8, 12 and 16h pi); but the various time points were also sequenced individually (**Figure 2, 4**, **Table 4**). For the library preparation, we used the ONT 1D strand-switching cDNA by ligation protocol (Version: SSE_9011_v108_revS_18Oct2016), the Ligation Sequencing 1D kit (SQK-LSK108, Oxford Nanopore Technologies) and the NEBNext End repair / dA-tailing Module NEB Blunt/TA Ligase Master Mix (New England Biolabs), according the manufacturers' recommendations. Briefly, 50ng of the polyA(+)-selected RNA samples were subjected to RT using PolyT-containing anchored oligonucleotides [(VN)T20; ordered from Bio Basic, Canada], (**Table 3**), dNTPs (10mM, Thermo Scientific), Superscript IV Reverse Transcriptase Kit (Life Technologies), RNase OUT (Life Technologies) and strand-switching oligonucleotides with three O-methyl-guanine RNA bases (PCR_Sw_mod_3G; ordered from Bio Basic, Canada). First-strand cDNAs were generated at 50°C for 10min incubation, which was followed by the strand-switching step at 42°C for 10min and a final inactivation step at 80°C for 10min. Double-stranded cDNAs (5μl from each) were amplified by using KAPA HiFi DNA Polymerase (Kapa Biosystems), Ligation Sequencing Kit Primer Mix (supplied by the 1D Kit) and a Veriti Thermal Cycler (Applied Biosystems). The initial denaturation was conducted at 95°C for 30sec (1 cycle), the denaturation was carried out at 95°C for 15sec (15

cycles), the annealing step was set to 62°C for 15sec (15 cycles), while the elongation was set to 65°C for 4min (15 cycles). The final extension step was carried out at 65°C for 1 min. NEBNext End repair / dA-tailing Module (New England Biolabs), and the NEB Blunt/TA Ligase Master Mix (New England Biolabs) was used for end-repair and for adapter ligations, respectively. The adapter sequences were provided by the 1D kit. Agencourt AMPure XP magnetic beads (Beckman Coulter) were used to purify the samples following each enzymatic step. The Qubit Fluorometer (Life Technologies Qubit 2.0) and the Qubit (ds)DNA HS Assay Kit were applied to measure the quantity of the libraries. Samples were loaded on R9.4 SpotON Flow Cells, and base calling was performed using Albacore v1.2.6. The PCR amplicons derived from the mixed RNA sample were size-selected manually, and then ran on Ultrapure Agarose gel (Thermo Fischer Scientific), followed by the isolation of 500bp+ fragments using the Zymoclean Large Fragment DNA Recovery Kit (Zymo Research). The individually sequenced samples were labeled with barcodes applying a combination of two ONT protocols: first, the 1D protocol was used, but after the first end-prep step, we switched to the 1D PCR barcoding (96) genomic DNA (SQK-LSK108) protocol (version: PBGE96_9015_v108_revS_18Oct2016, updated 25/10/2017), which was then followed by the barcode ligation step using the ONT PCR Barcoding Kit 96 (EXP-PBC096): the barcode adapters (**Table 3**) were ligated to the end-prepped cDNA samples using the NEB Blunt/TA Ligase Master Mix (New England Biolabs), then they were amplified by PCR with Kapa HiFi DNA Polymerase. The quantities of the libraries were measured by Qubit 2.0 (**Table 4**).

Table 4.

Library	Starting RNA	Starting RNA amount (ng)	cDNA amount (PCR product, ng)	Library used for sequencing (ng)	Barcode #	Number of flow cells
1D cDNA	polyA(+) mixed	29	253	65	-	1
1D cDNA	polyA(+) mixed	29	251	48	-	
1D cDNA	polyA(+) 1h	50	117	150	A1	1
1D cDNA	polyA(+) 2h	50	387		A2	
1D cDNA	polyA(+) 3h	50	360	300	A3	1
1D cDNA	polyA(+) 4h	50	180		A4	
1D cDNA	polyA(+) 6h	50	207		A5	
1D cDNA	polyA(+) 8h	50	103		A6	

1D cDNA	polyA(+) 12h	50	130		A7	
dRNA	polyA(+) mixed	60	no PCR	10,2	-	1
Cap-Seq	total RNA (1, 2, 3h)	2 µg	240	240	-	1
Cap-Seq	total RNA (4, 6, 8, 12, 16h)	2 µg	1125	320	-	1

ONT MinION cDNA-sequencing on Cap-selected samples For more precise identification of the 5'-ends of the full-length transcripts, a Cap-selection method was applied and combined with the ONT 1D cDNA library preparation protocol. The cDNAs were generated from a mixed total RNA sample (containing RNAs from 1, 2, 3, 4, 6, 8, 12 and 16h pi, **Table 1 and 4**) by using the TeloPrime Full-Length cDNA Amplification Kit (Lexogen). The protocol contains a PCR amplification step. The specificity of the products was checked by qPCR (Rotor-Gene Q). A VACV gene-specific primer (D1R gene, **Table 3**) and ABsolute qPCR SYBR Green Mix (Thermo Fisher Scientific) were used. The amplified PolyA(+)- and Cap-selected samples were subjected to the ONT's 1D strand-switching cDNA by a ligation method (ONT Ligation Sequencing 1D kit); they were end-repaired, then ligated to the 1D adapters (NEBNext End repair / dA-tailing Module NEB Blunt/TA Ligase Master Mix).

ONT MinION – dRNA sequencing In order to avoid the potential PCR biases, the amplification-free Direct RNA sequencing (DRS) protocol (Version: DRS_9026_v1_revM_15Dec2016) from the ONT's was applied. The library was prepared from a PolyA(+) mixture of 8 time points (1, 2, 3, 4, 6, 8, 12 and 16h pi, **Table 4**). RNA was mixed with the RT (oligodT-containing T10) adapter (provided by the ONT Direct RNA Sequencing Kit; SQK-RNA001) and T4 DNA ligase (2M U/ml; New England BioLabs). Following a 10-minute incubation, the first-strand cDNAs were generated with SuperScript III Reverse Transcriptase (Life Technologies), according to the DRS protocol, at 50°C for 50min, then at 70°C for 10min in a Veriti Thermal Cycler. Samples were purified by using Agencourt AMPure XP Beads (Beckman Coulter). XP Beads were handled before use with RNase OUT (40 U/µl; Life Technologies; 2U enzyme/1 µl bead). Washed samples were eluted in Ambion Nuclease-Free Water (Thermo Fisher Scientific). An RMX sequencing adapter was ligated to the

samples with NEBNext Quick Ligation Reaction Buffer (New England BiceoLabs) T4 DNA ligase. Samples were washed with RNase OUT-treated XP beads and Wash Buffer (part of the DRS Kit). Finally, the samples were eluted in 21µl Elution Buffer (provided by the DRS Kit). The concentrations of the reverse-transcribed and adapter-ligated RNAs were measured using the Qubit 2.0 Fluorometer and Qubit dsDNA HS Assay Kit (Life Technologies). The ONT cDNA libraries, the Cap-selected samples, and the direct RNA libraries were loaded on 3, 2 and 1 ONT R9.4 SpotON Flow Cells for sequencing, respectively. The runs were carried out using MinKNOW. Voltage levels were set and reset in line with the suppliers' recommendations.

Data analysis and visualization

The PacBio RSII reads of insert (ROI) reads were generated using the RS_ReadsOfInsert protocol of the SMRT Analysis v2.3.0, with the following settings: Minimum Full Passes = 1, Minimum Predicted Accuracy = 90, Minimum Length of Reads of Insert = 1, Maximum Length of Reads of Insert = No Limit. These consensus reads were mapped using GMAP [27] (version 2017-09-30) with the default settings. GMAP was chosen in this work because we have found it the best long-read aligner in our earlier publications [18, 19, 20, 21, 22, 23, 24]. GMAP have also produced to the best alignment results in other studies [e.g. 28]. The ROIs from the Sequel data were created using SMRT Link5.0.1.9585. The ONT's Albacore software v.2.0.1 was applied for the MinION base calling. This basecaller is able to identify the nucleotide sequences directly from raw sequencing data. The reads were aligned with the GMAP program using the same setting as described above. The raw reads were aligned to the reference genome of the virus (LT966077.1) and the host cell (*Chlorocebus sabaeus*): GenBank assembly accession: GCA_000409795.2 (latest); RefSeq assembly accession: GCF_000409795.2 (latest)]. In-house routines were used to acquire the quality information presented in this data note. The codes have been archived on Github [29]. Bedtools genomecov software [30] was used to generate coverage files with the following parameters: -split – ibam. The output bed files

from cDNA sequencing were visualized by Circos plot [31] (**Figure 5**), while the low-coverage dRNA-Seq data was shown by Integrative Genomics Viewer (IGV, [32]).

Data summary

The raw sequencing reads were mapped to both the VACV reference genome and to the host genome. In this study, we generated full-length transcripts of VACV and the CV-1 cells, yielding about 3,17 Gb of mapped sequencing data. Sequencing on the RSII and Sequel platforms yielded 86,728 and 850,803 ROIs aligned to the viral and the host genome, respectively. The utilized nanopore-based cDNA sequencing approaches resulted in altogether 413,497 VACV specific reads (**Table 5**), while we obtained 155,876 reads from the Cap-selected samples. The different MinION sequencing methods yielded altogether 1,590,975 reads that mapped to the host genome. The ratio of viral transcripts is 21.9% on average in our samples. The exact ratio is dependent on the titer of the virus used for the infection, as well as on the stage of the viral life cycle at the examination period. The sequencing method affects the ratio of read counts between the virus and host cell: e.g. the MinION 1D-Seq method yields a higher amount of shorter reads compared to the PacBio Sequel technique. The VACV transcripts are relatively short compared to the host or to other large DNA viruses (such as herpesviruses and baculoviruses), which is assumed to result in the relatively high ratio of viral reads compared to the host reads in the MinION samples (**Figure 6**).

The average lengths of ROIs aligning to the VACV genome were 1,098 bp for PacBio RSII, 1,157 bp for the Sequel. The MinION average read-lengths were as follows: 557 bp for ONT barcoded cDNA sequencing, 792bp for the cDNA-Seq, and 965bp for the Cap-selected samples (**Table 5**). The average read-length produced by dRNA sequencing was 537bp. It should be noted that the library preparation and size-selection methods resulted in different samples in terms of length; all library preparation methods resulted in longer average read-length aligning to the host genome than to the

viral genome (**Table 5, Figure 7**). We have compared the average aligned read-length of cellular transcripts obtained in this and in other studies in **Figure 8** [18, 19, 21, 24, 33, 34, 35].

The various sample preparation and sequencing techniques produced different read-length, read number and precision, as well as different artifacts. There is a relatively large difference between the PacBio and ONT sequencing approaches concerning the quality of the sequencing reads: PacBio technique produces much fewer mismatches and INDELs than nanopore sequencing. The various sequencing platforms recommend different cDNA production kits, which contain different enzymes and primers for both the RT and PCR. The various primers and library preparation conditions could produce artifacts; however, these can be easily filtered out if we compare the results of different methods. The PacBio MagBead loading selectively eliminates the short fragments (<1,000bp). While on the one hand, removal of incomplete cDNAs can be advantageous, at the same time, it is unfavorable, as we are unable to detect the shorter transcripts and RNA isoforms. Our data demonstrates that the ONT MinION sequencing resulted in higher error rates for both INDELS and mismatches in comparison to the PacBio systems (**Table 5**). The composition of the errors of the three platforms (RSII, Sequel, and MinION) and the various library preparation techniques (dRNA-Seq, Cap-Seq, etc.) are different. Mismatches are the most common errors in ONT cDNA-Seq, which is consistent with others' data [36]. In agreement with the previously published data [36], our results also indicate that insertions are the least frequent errors in ONT MinION sequencing. In accordance with others' results [37], our dRNA reads have higher deletion error rate than either of the cDNA data sets and lower than those of the ONT cDNA-Seq samples, which might be the result of the lower coverage of the dRNA-Seq. In contrast to others' results [36], deletions are the major errors in our PacBio RSII dataset. The quality of the Sequel dataset shows 'coverage-specificity': mismatches are the major errors in the lower-coverage samples, which complies with others' data [36], while, contrary to the same report in that the insertions are more frequent in the higher coverage samples in our data set. The RSII and the Sequel platforms produces the same error rate. Conversely, our data show somewhat higher error rate for the Sequel, which might be the result of the different library

preparation approaches. In sum, the absolute error rate of both PacBio platforms is low, while the higher ONT error rate is "compensated" by the higher coverage. It is worth mentioning that read quality is not essential for transcriptome analysis if well-annotated genomes are available.

Our transcriptomic survey yielded an extremely high-coverage across the viral genome (**Figure 5**): 290.1 fold for the RSII, 138.6 fold for the Sequel, 550.5 fold for the barcoded MinION cDNA-Seq, 550.8 fold for the Cap-selected samples and 302.1 fold for the cDNA sequencing (more detailed information, including quality information are available in **Table 5** and **Additional file 2**). Our data show that the entire VACV genome is transcriptionally active, generating RNAs from both DNA strands. Our dataset also contains 1.56 Gb of raw data from Sequel sequencing, as well as from MinION dRNA-sequencing.

Table 5.

**A** Run #	**Major specificities of libraries**	**Number of mapped reads**	**Median Read-length**	**Median Aligned Read-length**	**Average Read-length ± SE**	**Average Aligned Read-length ± SE**	**Average insertion frequency ± SE**	**Average deletion frequency ± SE**	**Average mismatch frequency ± SE**	**Coverage**
1	RSII mix no size selection	110 *	1331.5	1310	1385.66 ± 46.40	1361.5 ± 46.58	0.04 ± 0.0096	0.21 ± 0.0603	0.02 ± 0.0056	0,7684
2	RSII mix random primed	31	660	572	799.74 ± 88.58	714.87 ± 89.19	0.09 ± 0.0447	0.13 ± 0.0634	0.02 ± 0.0117	0,1137
3	RSII mix BluePippin size selection: 0.8-5kb+	23802	1008	885	1152.67 ± 9.28	937.94 ± 3.12	0.25 ± 0.0084	0.30 ± 0.0056	0.11 ± 0.0047	114,55
4	RSII BluePippin size selection: 0.8-2kb	1283	1055	938	1103.85 ± 10.87	978.35 ± 9.58	0.07 ± 0.0070	0.15 ± 0.0136	0.07 ± 0.0291	6,4407
5	RSII BluePippin size selection: 2-3kb	5029	998	897	1062.55 ± 5.78	947.92 ± 5.23	0.12 ± 0.0071	0.11 ± 0.0059	0.03 ± 0.0053	24,460
6	RSII BluePippin size selection: 3-5kb	20103	1053	942	1063.17 ± 2.89	946.56 ± 2.57	0.14 ± 0.0038	0.16 ± 0.0042	0.04 ± 0.0032	97,639
7	RSII BluePippin size selection: 5kb+	8848	1082	990	1118.95 ± 4.70	1022.00 ± 4.45	0.11 ± 0.0050	0.19 ± 0.0062	0.04 ± 0.0039	46,399
8	Sequel 1h	455	902	701	1149.16 ± 40.59	774.48 ± 22.65	0.44 ± 0.0909	0.19 ± 0.0544	0.55 ± 0.1298	1,8081
9	Sequel 2h	527	882	725	1054.95 ± 29.44	821.98 ± 23.19	0.19 ± 0.0453	0.09 ± 0.0120	0.28 ± 0.0925	2,2227
10	Sequel 3h	1068	988.5	828.5	1125.82 ± 19.84	909.89 ± 15.13	0.20 ± 0.0459	0.10 ± 0.0090	0.25 ± 0.0597	4,9862
11	Sequel 4h	809	840	676	1128.46 ± 36.37	727.83 ± 19.74	0.34 ± 0.0672	0.13 ± 0.0141	0.45 ± 0.0942	3,0213
12	Sequel 4h 2nd	4522	1006	885	1227.22 ± 10.32	969.31 ± 6.94	0.50 ± 0.0213	0.17 ± 0.0057	0.49 ± 0.0390	22,490
13	Sequel 6h	3031	953	753	1116.97 ± 13.58	861.71 ± 10.00	0.23 ± 0.0184	0.09 ± 0.0049	0.24 ± 0.0337	13,401
14	Sequel 8h	5482	996	878	1177.50 ± 9.61	981.88 ± 7.95	0.21 ± 0.0133	0.10 ± 0.0041	0.12 ± 0.0141	27,619
15	Sequel 8h 2nd	11628	1089	907.5	1277.91 ± 6.85	1057.00 ± 5.46	0.26 ± 0.0101	0.13 ± 0.0031	0.14 ± 0.0089	63,066
16	MinION 1D cDNA Manual size selection: 500bp+	89778	606	479	792.48 ± 1.89	655.79 ± 1.63	3.26 ± 0.0063	6.04 ± 0.0068	6.63 ± 0.0091	302,10
17	MinION dRNA	1259	465	408	537.17 ± 9.76	524.67 ± 10.47	2.01 ± 0.0537	8.84 ± 0.0832	5.57 ± 0.0620	3,3894
18	MinION Cap-selection	155876	783	637	965.34 ± 1.36	688.73 ± 0.74	3.29 ± 0.0047	5.28 ± 0.0042	6.74 ± 0.0066	550,86
19	MinION 1D cDNA barcoded 1h	17048	485	286	589.68 ±	392.62 ±	2.96 ±	4.54 ±	4.61 ±	31,358

					2.19	2.00	0.0156	0.0169	0.0186	
20	MinION 1D cDNA barcoded 2h	94125	462	281	518.55 ± 0.69	334.78 ± 0.59	3.04 ± 0.0071	4.57 ± 0.0073	4.66 ± 0.0081	147,62
21	MinION 1D cDNA barcoded 3h	22029	466	283	521.58 ± 1.32	337.82 ± 1.18	2.80 ± 0.0138	4.39 ± 0.0142	4.51 ± 0.0163	34,865
22	MinION 1D cDNA barcoded 4h	41700	469	284	526.01 ± 1.01	342.85 ± 0.91	3.01 ± 0.0123	4.46 ± 0.0105	4.64 ± 0.0120	66,981
23	MinION 1D cDNA barcoded 6h	42082	487	291	570.35 ± 1.29	383.47 ± 1.19	2.84 ± 0.0109	4.47 ± 0.0107	4.88 ± 0.0118	75,602
24	MinION 1D cDNA barcoded 8h	48437	513	303	648.63 1.70	447.74 ± 1.55	2.92 ± 0.0109	4.37 ± 0.0098	4.70 ± 0.0109	101,60
25	MinION 1D cDNA barcoded 12h	57039	469	284	526.76 ± 0.85	346.08 ± 0.79	2.85 ± 0.0092	4.47 ± 0.008	4.55 ± 0.0101	92,483

B

Run #	Major specificities of libraries	Number of mapped reads	Median Read-length	Median Aligned Read-length	Average Read-length ± SE	Average Aligned-lead Length ± SE	Average insertion frequency ± SE	Average deletion frequency ± SE	Average mismatch frequency ± SE	Coverage
1	RSII mix no size selection	512 *	1393.5	1354	1446.21 ± 23.62	1374.1 ± 24.84	0.29 ± 0.0968	0.66 ± 0.063	1.24 ± 0.0957	0,0002
2	RSII mix random primed	2905	847	744	945.83 ± 9.04	840.11 ± 9.32	0.22 ± 0.0399	0.46 ± 0.0200	1.03 ± 0.0524	0,0008
3	RSII mix BluePippin size selection: 0.8-5kb+	50200	1081	933	1132.69 ± 2.05	933.03 ± 2.40	0.60 ± 0.0171	0.57 ± 0.0895	1.27 ± 0.0099	0,0167
4	RSII BluePippin size selection: 0.8-2kb	15206	1090	956	1199.54 ± 4.16	1016.52 ± 4.77	0.57 ± 0.0299	0.49 ± 0.0159	1.22 ± 0.0173	0,0055
5	RSII BluePippin size selection: 2-3kb	8752	1238	1141	1257.92 ± 4.21	1129.90 ± 4.83	0.32 ± 0.0255	0.52 ± 0.0143	1.45 ± 0.0241	0,0035
6	RSII BluePippin size selection: 3-5kb	68766	1015	789	1151.05 ± 2.51	881.73 ± 2.33	0.68 ± 0.0151	0.73 ± 0.0085	1.82 ± 0.0102	0,0217
7	RSII BluePippin size selection: 5kb+	16024	1033	830	1092.51 ± 3.52	852.45 ± 4.33	0.68 ± 0.0348	0.65 ± 0.0180	2.30 ± 0.0221	0,0048
8	Sequel 1h	38239	1341	922	1488.88 ± 4.38	1063.58 ± 4.33	0.53 ± 0.0131	0.29 ± 0.0063	1.49 ± 0.0138	0,0145
9	Sequel 2h	38255	1112	997	1318.41 ± 4.40	1191.07 ± 4.09	0.38 ± 0.0081	0.18 ± 0.0026	1.41 ± 0.0115	0,0163
10	Sequel 3h	68500	1317	1196	1453.95 ± 3.34	1304.42 ± 2.95	0.51 ± 0.0070	0.22 ± 0.0024	1.15 ± 0.0075	0,0320
11	Sequel 4h	42379	1240	1088	1448.78 ± 5.32	1185.50 ± 4.01	0.68 ± 0.0114	0.28 ± 0.0041	1.13 ± 0.0096	0,0180
12	Sequel 4h 2nd	233709	1796	1340	1865.73 ± 1.69	1302.18 ± 1.96	0.84 ± 0.0071	0.26 ± 0.0022	0.71 ± 0.0031	0,1090
13	Sequel 6h	101745	1403	1268	1530.68 ± 2.86	1368.60 ± 2.57	0.53 ± 0.0065	0.22 ± 0.0020	1.25 ± 0.0064	0,0499
14	Sequel 8h	101624	1531	1394	1642.16 ± 3.03	1505.74 ± 3.00	0.50 ± 0.0067	0.21 ± 0.0020	1.85 ± 0.0078	0,0548
15	Sequel 8h 2nd	63987	1656	1058	1720.46 ± 3.48	1152.01 ± 3.82	1.04 ± 0.0208	0.33 ± 0.0056	1.16 ± 0.0079	0,0264
16	MinION 1D cDNA Manual size selection: 500bp+	293048	775	481	1127.15 ± 2.36	763.42 ± 1.65	4.25 ± 0.0120	5.63 ± 0.0072	6.62 ± 0.0073	0,0801
17	MinION dRNA	14757	510	376	606.26 ± 4.10	495.05 ± 3.93	3.06 ± 0.0453	7.86 ± 0.0402	5.92 ± 0.0305	0,0026
18	MinION Cap-selection	327964	664	476	815.83 ± 0.87	502.38 ± 0.48	3.81 ± 0.0091	5.60 ± 0.0053	7.02 ± 0.0061	0,0590
19	MinION 1D cDNA barcoded 1h	69060	712	478	914.17 ± 2.18	674.09 ± 2.18	3.35 ± 0.0125	5.11 ± 0.0117	5.72 ± 0.0105	0,0166
20	MinION 1D cDNA barcoded 2h	474008	593	389	775.40 ± 0.77	558.52 ± 0.76	3.30 ± 0.0047	5.15 ± 0.0045	5.64 ± 0.0040	0,0949
21	MinION 1D cDNA barcoded 3h	88064	596	395	755.74 ± 1.62	539.09 ± 1.62	3.00 ± 0.0113	5.03 ± 0.0103	5.37 ± 0.0094	0,0170
22	MinION 1D cDNA barcoded 4h	134090	595	390	743.67 ± 1.25	520.05 ± 1.26	3.03 ± 0.0096	5.06 ± 0.0086	5.39 ± 0.0078	0,0249
23	MinION 1D cDNA barcoded 6h	106989	610	396	769.19 ± 1.49	535.50 ± 1.49	3.04 ± 0.0109	5.06 ± 0.0099	5.42 ± 0.0088	0,0205
24	MinION 1D cDNA barcoded 8h	51071	645	390	852.11 ± 2.46	543.46 ± 2.54	3.02 ± 0.0214	4.49 ± 0.165	4.92 ± 0.0142	0,0099
25	MinION 1D cDNA barcoded 12h	31924	497	45	611.37 ± 1.81	252.51 ± 2.07	2.93 ± 0.0352	3.26 ± 0.0242	3.76 ± 0.0198	0,0028

The read-length distributions for the dataset are shown in **Figure 9** (reads mapped to the VACV genome), as well as in **Figure 10** and **Figure 11** (data aligned to the VACV and to the host genome). Detailed information are available in **Additional file 3**.

The read counts aligned to the mRNAs have been calculated (**Table 6**). Most of the host-specific reads align to the coding region in this dataset (the values vary between 43-87% based on the read counts and between 35-85% if we compare the number of nucleotides).

Table 6.

Run #	Sample	FULL GENOME			CODING REGION			Ratio of mRNAs (%)	
		Read count	Read-length	Number of nucleotides	Read count	Read-length	Number of nucleotides	Nucleotides	Read counts
1	RSII mix no size selection	512	1,446.21	740,464	417	1,469.98	612,985	82.78	81.44
2	RSII mix random primed	2,905	945.83	2,747,655	2,025	994.69	2,014,263	73.30	69.70
3	RSII mix BluePippin size selection: 0.8-5kb+	68,766	1,151.05	79,153,724	46,804	1,208.04	56,541,459	71.43	68.06
4	RSII BluePippin size selection: 0.8-2kb	8,752	1,257.92	11,009,363	7,207	1,293.62	9,323,162	84.68	82.34
5	RSII BluePippin size selection: 2-3kb	15,206	1,199.54	18,240,240	11,653	1,261.14	14,696,178	80.57	76.63
6	RSII BluePippin size selection: 3-5kb	50,200	1,132.69	56,861,382	37,469	1,184.09	44,367,034	78.02	74.63
7	RSII BluePippin size selection: 5kb+	16,024	1,092.51	17,506,399	9,887	1,167.09	11,539,097	65.91	61.70
8	Sequel 1h	38,239	1,488.88	56,933,507	28,278	1,486.10	42,023,982	73.81	73.95
9	Sequel 2h	38,255	1,318.41	50,435,790	31,032	1,376.16	42,705,033	84.67	81.11
10	Sequel 3h	68,500	1,453.95	99,595,716	58,023	1,495.74	86,787,487	87.13	84.70
11	Sequel 4h	42,379	1,448.78	61,397,855	35,167	1,503.90	52,887,891	86.13	82.98
12	Sequel 4h 2nd	233,709	1,865.73	436,038,400	168,856	1,815.09	306,489,055	70.28	72.25
13	Sequel 6h	101,745	1,530.68	155,739,727	83,390	1,584.56	132,137,271	84.84	81.95
14	Sequel 8h	101,624	1,642.16	166,883,807	77,056	1,775.76	136,833,599	81.99	75.82
15	Sequel 8h 2nd	63,987	1,720.46	110,087,571	42,229	1,742.19	73,571,350	66.82	65.99
16	MinION 1D cDNA Manual size selection: 500bp+	293,048	1,127.15	330,309,951	153,439	1,227.20	188,301,397	57.00	52.35
17	MinION dRNA	14,757	606.26	8,946,617	8,024	704.64	5,654,034	63.19	54.37
18	MinION Cap-selection	327,964	815.83	267,566,060	258,212	801.90	207,062,394	77.38	78.73
19	MinION 1D cDNA barcoded 1h	69,060	914.17	63,133,198	54,218	975.11	52,868,770	83.74	78.50
20	MinION 1D cDNA barcoded 2h	474,008	775.40	367,550,004	367,112	817.77	300,215,563	81.68	77.44
21	MinION 1D cDNA barcoded 3h	88,064	755.74	66,553,829	66,256	800.55	53,041,624	79.69	75.23
22	MinION 1D cDNA barcoded 4h	134,090	743.67	99,719,660	99,611	792.43	78,935,516	79.15	74.28
23	MinION 1D cDNA barcoded 6h	106,989	769.19	82,295,542	78,669	821.03	64,590,390	78.48	73.52
24	MinION 1D cDNA barcoded 8h	51,071	852.11	43,518,439	30,935	981.50	30,362,708	69.76	60.57
25	MinION 1D cDNA barcoded 12h	31,924	611.37	19,517,535	11,094	773.70	8,583,438	43.97	34.75

We mapped the raw data to the VACV and to the host mRNAs. Ten viral and ten host genes that are expressed at every examined time point were chosen for a heatmap analysis (**Figure 12**). Only the full-length transcripts were calculated for the analysis. A read was considered as full-length if it contained the polyA-tail as well as the 5'- and 3' adapter sequences. Plus/minus 20bp range was set to and from the transcription start and end sites.

Conclusions and Reuse Potential

The present study generated data using state-of-art sequencing technologies (PacBio RSII and Sequel, as well as the ONT MinION platforms, applying a new protocol for barcoding the samples), These data allow a time-course look at the full-length transcriptome of VACV, as well as the CV-1 host cell line.

The dataset was primarily produced for the dynamic characterization of VACV transcriptome. Another aim was to generate a deep coverage long-read dataset for the analysis of the different transcript isoforms, including length- (5'-ends and 3'-ends) variants, mono-, bi-, polycistronic transcripts, and also to define full-length transcripts produced by the various viral genes. This dataset is useful in understanding the complexity of the genetic regulation of VACV. The provided dataset can also be used to investigate the effect of the viral infection on the gene expression of the host.

The provided binary alignment (BAM) files contain reads already aligned to the VACV and to the host genome. These aligned reads can be further analyzed by comparing them to the results of various long read aligners (e.g. BLASR [38]; NGMLR [39]; Minimap2 [40]), and bioinformatics tools (e.g. samtools [41] or bedtools [42]). Other long-read sequencing programs or pipelines (e.g.: SQANTI [43]) can be tested using this dataset.

These data can be visualized by using different programs such as the Geneious [44], Artemis [45], or IGV [32]. Data can be useful for testing novel bioinformatics pipelines or to improve those already available. The files contain terminal polyA sequences as well as the 5'and 3' adapter sequences,

which can be used to determine the orientations of the reads. The dataset contains the raw dataset from dRNA sequencing (fast5.tar.gz), which can be further analyzed by using the Tombo software package [46], which enables the detection and visualization of modified nucleotides, such as the 6-methyladednine (m6A), the most common internal mRNA modification described in eukaryotes [47, 48, 49], as well as in viruses [50, 51, 52], or the 5-methylcitosine (m5C), which is another abundant modification recently confirmed in mRNA [53, 54, 55, 56]. According to our best knowledge, these modifications have not yet been shown in the *Poxviridae* family. The raw data provided from PacBio Sequel sequencing can be used to improve existing base caller algorithms or potentially to develop novel algorithms, and further, the data contain the full set of quality values and kinetic measurements.

This dataset can be used to identify novel VACV and CV-1 transcripts and RNA isoforms including splice variants of the host transcripts, TSS and TES variants, as well as polycistronic transcripts of the virus and the host, to examine the effect of VACV infection on the host gene expression at the different stages of viral life cycle, as well as for the comparison of the quality and length of the sequencing reads derived from different sequencing platforms. The various library preparation methods can also be compared with one another. The provided data could help understanding the logic of gene expression control of Poxviruses, and can also be used to design gene expression vectors.

Availability of source code

Project home page: e.g. https://github.com/Szunyike/SAM-Statistic-2018

Operating system(s): Windows

Programming language: VB.NET

Other requirements: NET framework

License: free

Availability of Supporting Data

All of the presented data was deposited in the European Nucleotide Archive under the accession number of PRJEB26434 (Characterization of the Vaccinia virus transcriptome) and PRJEB26430 (Dynamic characterization of the Vaccinia virus transcriptome).

Additional file 4. Correspondence between the file names of alignments deposited in ENA and the names that are used in this manuscript.

Competing interests

The authors declare that there are no conflicts of interest.

Funding

This study was supported by the NKFIH OTKA [K 128247] and by the Swiss-Hungarian Cooperation Programme [SH/7/2/8] to ZB, by the NKFIH OTKA [FK 128252], by the Eötvös Scholarship of the Hungarian State to DT and by Bolyai János Scholarship of the Hungarian Academy of Sciences to DT. The project was also supported by the NIH Centers of Excellence in Genomic Science (CEGS) Center for Personal Dynamic Regulomes [5P50HG00773502] to MS.

Author Contributions

DT, DB, MS and ZB conceived and designed the experiments. DB propagated the cells and viruses. DT and IP prepared RNA samples and generated cDNAs. DT prepared the sequencing libraries and performed the PacBio and ONT sequencing. DT, AS, IP and ZB analyzed the data. DT and ZB wrote the manuscript. ZB supervised the project. All authors have read and approved the final version of the manuscript.

Abbreviations

m5C - 5-methyl cytosine

m6A - 6-methyl adenine

CV-1 - African green monkey (*Chlorocebus sabaeus*) kidney fibroblast cells

ATCC - American Type Culture Collection

CAGE - cap analysis of gene expression

dRNA - direct RNA

E - early

FBS - fetal bovine serum

IE - immediate-early

I - intermediate

Iso-Seq - Isoform sequencing

L - late

LRS - Long-read sequencing

ORF - open reading frame

ONT - Oxford Nanopore Technologies

PacBio - Pacific Biosciences

PBS - phosphate-buffered saline

pi - post-infection

RSII - Real-Time Sequencer II

SRS - short-read sequencing

TES - transcription end site

TSS - transcription start site

VACV - Vaccinia virus

WR - Western Reserve

References

1. Yang Z, Cao S, Martens CA et al. Deciphering Poxvirus Gene Expression by RNA Sequencing and Ribosome Profiling. *J Virol*. 2015;89(13): 6874–6886. doi: 10.1128/JVI.00528-15

2. Benfield CT, Ren H, Lucas SJ et al. Vaccinia virus protein K7 is a virulence factor that alters the acute immune response to infection. *J Gen Virol*. 2013;94(Pt 7): 1647–1657. doi: 10.1099/vir.0.052670-0

3. Pauli G, Blümel J, Burger R et al. Orthopox Viruses: Infections in Humans. *Transfus Med Hemother*. 2010;37(6): 351–364. doi: 10.1159/000322101

4. Wyatt LS, Xiao W, Americo JL et al. Novel Nonreplicating Vaccinia Virus Vector Enhances Expression of Heterologous Genes and Suppresses Synthesis of Endogenous Viral Proteins. *mBio*. 2017;8(3): e00790-17. doi: 10.1128/mBio.00790-17

5. Broyles SS. Vaccinia virus transcription. *J Gen Virol*. 2003;84:2293–2303. doi: 10.1099/vir.0.18942-0

6. Schramm B, Locker JK. Cytoplasmic Organization of POXvirus DNA Replication. *Traffic* 2005;6:839–846. doi: 10.1111/j.1600-0854.2005.00324.x

7. Assarsson E, Greenbaum JA, Sundström M et al. Kinetic analysis of a complete poxvirus transcriptome reveals an immediate-early class of genes. *Proc Natl Acad Sci U S A*. 2008;105(6):2140-5. doi: 10.1073/pnas.0711573105.

8. Davison AJ, Moss B. Structure of vaccinia virus early promoters. *J Mol Biol*. 1989; 210(4):749–769.

9. Davison AJ, Moss B. Structure of vaccinia virus late promoters. *J Mol Biol*. 1989; 210(4):771–784.

10. Baldick CJ, Jr, Keck JG, Moss B. Mutational analysis of the core, spacer, and initiator regions of vaccinia virus intermediate-class promoters. *J Virol*. 1992;66:4710–4719.

11. Broyles SS, Moss B. Homology between RNA polymerases of poxviruses, prokaryotes, and eukaryotes: nucleotide sequence and transcriptional analysis of vaccinia virus genes encoding 147-kDa and 22-kDa subunits. *Proc Natl Acad Sci U S A*. 1986;83(10):3141-5.

12. Wittek R, Cooper JA, Barbosa E et al. Expression of the vaccinia virus genome: Analysis and mapping of mRNAs encoded within the inverted terminal repetition. *Cell*. 1980;21(2):487–493.

13. Yang Z, Bruno DP, Martens CA et al. Simultaneous high-resolution analysis of vaccinia virus and host cell transcriptomes by deep RNA sequencing. *PNAS*. 2010;107(25):11513-11518. https://doi.org/10.1073/pnas.1006594107

14. Yang Z, Bruno DP, Martens CA et al. Genome-Wide Analysis of the 5′ and 3′ Ends of Vaccinia Virus Early mRNAs Delineates Regulatory Sequences of Annotated and Anomalous Transcripts. *J Virol*. 2011;85(12): 5897–5909. doi: 10.1128/JVI.00428-11

15. Yang Z, Maruri-Avidal L, Sisler J et al. Cascade regulation of vaccinia virus gene expression is modulated by multistage promoters. *Virology* 2013;447(1–2):213-220. doi.org/10.1016/j.virol.2013.09.007

16. Rubins KH, Hensley LE, Bell GW et al. Comparative analysis of viral gene expression programs during poxvirus infection: a transcriptional map of the vaccinia and monkey pox genomes. *PLoS One*. 2008;3(7):e2628. 10.1371/journal.pone.0002628

17. Yang Z, Martens CA, Bruno DP et al. Pervasive initiation and 3′ end formation of poxvirus post-replicative RNAs. *J Biol Chem*. 2012;287:31050–31060. doi: 10.1074/jbc.M112.390054.

18. Tombácz D, Csabai Z, Oláh P et al. Full-Length Isoform Sequencing Reveals Novel Transcripts and Substantial Transcriptional Overlaps in a Herpesvirus. *PLoS One*. 2016;11(9) e0162868. doi: 10.1371/journal.pone.0162868.

19. Tombácz D, Csabai Z, Szűcs A et al. Long-Read Isoform Sequencing Reveals a Hidden Complexity of the Transcriptional Landscape of Herpes Simplex Virus Type 1. *Front Microbiol*. 2017;8:1079. doi: 10.3389/fmicb.2017.01079.

20. Balázs Z, Tombácz D, Szűcs A et al. Long-Read Sequencing of Human Cytomegalovirus Transcriptome Reveals RNA Isoforms Carrying Distinct Coding Potentials. *Sci Rep*. 2017;7(1):15989. doi: 10.1038/s41598-017-16262-z.

21. Balázs Z, Tombácz D, Szűcs A et al. Long-read sequencing of the human cytomegalovirus transcriptome with the Pacific Biosciences RSII platform. *Sci Data*. 2017;4:170194. doi: 10.1038/sdata.2017.194.

22. Moldován N, Tombácz D, Szűcs A et al. Multi-Platform Sequencing Approach Reveals a Novel Transcriptome Profile in Pseudorabies Virus. *Front Microbiol*. 2018;8:2708. doi: 10.3389/fmicb.2017.02708.

23. Tombácz D, Sharon D, Szűcs A et al. Transcriptome-wide survey of pseudorabies virus using next- and third-generation sequencing platforms. *Sci Data*. 2018;5:180119. doi: 10.1038/sdata.2018.119.

24. Moldován N, Tombácz D, Szűcs A et al. Third-generation Sequencing Reveals Extensive Polycistronism and Transcriptional Overlapping in a Baculovirus. *Sci Rep*. 2018;8(1):8604. doi: 10.1038/s41598-018-26955-8.

25. Yang L, Duff MO, Graveley BR et al. Genome-wide characterization of non-polyadenylated RNAs. *Genome Biol*. 2011;12(2):R16. doi: 10.1186/gb-2011-12-2-r16.

26. Zhang Y, Yang L, Chen LL. Life without A tail: new formats of long noncoding RNAs. Int J Biochem. *Cell Biol*. 2014;54:338-49. doi: 10.1016/j.biocel.2013.10.009.

27. Wu TD, Watanabe CK. GMAP: a genomic mapping and alignment program for mRNA and EST sequences. *Bioinformatics*. 2005;21(9):1859–75. doi: 10.1093/bioinformatics/bti310

28. Križanovic K, Echchiki A, Roux J, Šikic M: Evaluation of tools for long read RNA-seq splice-aware alignment. *Bioinformatics*. 2018;34(5):748-754. doi: 10.1093/bioinformatics/btx668.

29. Long-read sequencing data statistics. https://github.com/Szunyike/SAM-Statistic-2018. Accessed: Sept. 2018.

30. Quinlan AR. BEDTools: The Swiss-Army Tool for Genome Feature Analysis. *Curr Protoc Bioinformatics*. 2014;47:11.12.1-34. doi: 10.1002/0471250953.bi1112s47.

31. Krzywinski M, Schein J, Birol I et al. Circos: an information aesthetic for comparative genomics. *Genome Res*. 2009;19(9):1639-45. doi: 10.1101/gr.092759.109.

32. Robinson JT, Thorvaldsdóttir H, Winckler W et *al*. Integrative genomics viewer. *Nat. Biotechnol.* 2011;29: 24–26. doi: 10.1038/nbt.1754.

33. Prazsak I, Moldovan N, Tombacz D et al., Long-read Sequencing Uncovers a Complex Transcriptome Topology in Varicella Zoster Virus. *bioRxiv* 2018;399048; doi: https://doi.org/10.1101/399048.

34. Balázs Z, Tombácz D, Szűcs A et al. Dual platform long-read RNA-sequencing dataset of the human cytomegalovirus lytic transcriptome. *Front Genet*. 2018; In press | doi: 10.3389/fgene.2018.00432.

35. Tombácz D, Prazsák I, Moldován N et al. Lytic Transcriptome Dataset of Varicella Zoster Virus Generated by Long-read Sequencing. *Front Genet*. 2018; In press | doi: 10.3389/fgene.2018.00460.

36. Weirather JL, de Cesare M, Wang Ye et al. Comprehensive comparison of Pacific Biosciences and Oxford Nanopore Technologies and their applications to transcriptome analysis. Version 2. *F1000Res*. 2017; 6:100. doi: 10.12688/f1000research.

37. Garalde DR, Snell EA, Jachimowicz D et al.: Highly parallel direct RNA sequencing on an array of nanopores. *Nat Methods*. 2018;15:201–206. doi: 10.1038/nmeth.4577.

38. Chaisson M, Tesler G. Mapping single molecule sequencing reads using Basic Local Alignment with Successive Refinement (BLASR): Theory and Application. *BMC Bioinformatics*. 2012;13:238. doi: 10.1186/1471-2105-13-238.

39. Sedlazeck FJ, Rescheneder P, Smolka M et al. Accurate detection of complex structural variations using single-molecule sequencing. *Nat Methods*. 2018;15:461–468. doi: 10.1038/s41592-018-0001-7.

40. Li H. Minimap2: pairwise alignment for nucleotide sequences. *Bioinformatics*. 2018;34(18):3094-3100. doi: 10.1093/bioinformatics/bty191.

41. Li H, Handsaker B, Wysoker A et al. The Sequence Alignment/Map format and SAMtools. *Bioinformatics*. 2009;25(16):2078–2079. doi: 10.1093/bioinformatics/btp352.

42. Quinlan AR, Hall I. M. BEDTools: a flexible suite of utilities for comparing genomic features. *Bioinformatics*. 2010;26(6):841–842. doi: 10.1093/bioinformatics/btq033.

43. Tardaguilla M, de la Fuente L, Marti C et al. SQANTI: extensive characterization of long-read transcript sequences for quality control in full-length transcriptome identification and quantification. *Genome Res*. 2018;28, 396-411. doi: 10.1101/gr.222976.117.

44. Kearse M, Moir R, Wilson A et al. Geneious Basic: an integrated and extendable desktop software platform for the organization and analysis of sequence data. *Bioinformatics*. 2012;28(12):1647–1649. doi: 10.1093/bioinformatics/bts199.

45. Rutherford K, Parkhill J, Crook J et al. Artemis: sequence visualization and annotation. *Bioinformatics*. 2010;16(10):944-5.

46. Stoiber MH, Quick J, Egan R et al. De novo Identification of DNA Modifications Enabled by Genome-Guided Nanopore Signal Processing. *bioRxiv*. 2017;094672. doi: https://doi.org/10.1101/094672.

47. Desrosiers R, Friderici K, Rottman F. Identification of methylated nucleosides in messenger RNA from Novikoff hepatoma cells. *Proc Natl Acad Sci U S A*. 1974;71:3971–3975. doi: 10.1073/pnas.71.10.3971

48. Dominissini D, Moshitch-Moshkovitz S, Schwartz S et al. Topology of the human and mouse m6A RNA methylomes revealed by m6A-seq. *Nature*. 2012;485(7397):201-6. doi: 10.1038/nature11112.

49. Liu J, Jia G: Methylation modifications in eukaryotic messenger RNA. *J Genet Genomics*. 2014;41(1):21-33. doi: 10.1016/j.jgg.2013.10.002.

50. Fengchun Ye: RNA N6-adenosine methylation (m6A) steers epitranscriptomic control of herpesvirus replication. *Inflamm Cell Signal*. 2017;4(3): e1604.

51. Kennedy EM, Courtney DG, Tsai K, Cullen BR. Viral Epitranscriptomics. *J Virol*. 2017;91(9). pii: e02263-16. doi: 10.1128/JVI.02263-16.

52. Tsai K, Courtney DG, Cullen BR: Addition of m6A to SV40 late mRNAs enhances viral structural gene expression and replication. *PLoS Pathog*. 2018;14(2):e1006919. doi: 10.1371/journal.ppat.1006919.

53. Edelheit S, Schwartz S, Mumbach MR et al. Transcriptome-wide mapping of 5-methylcytidine RNA modifications in bacteria, archaea, and yeast reveals m5C within archaeal mRNAs. *PLoS Genet*. 2013;9(6):e1003602. doi: 10.1371/journal.pgen.1003602.

54. Khoddami V, Cairns BR: Identification of direct targets and modified bases of RNA cytosine methyltransferases. *Nat Biotechnol*. 2013;31(5):458-64. doi: 10.1038/nbt.2566.

55. Amort T, Rieder D, Wille A et al. Distinct 5-methylcytosine profiles in PolyA RNA from mouse embryonic stem cells and brain. *Genome Biol*. 2017;18(1):1. doi: 10.1186/s13059-016-1139-1.

56. Hussain S, Aleksic J, Blanco S et al. Characterizing 5-methylcytosine in the mammalian epitranscriptome. *Genome Biol*. 2013;14(11):215. doi: 10.1186/gb4143.

Legend to Figures

Figure 1. Distribution of sequencing reads.

A. The stacked bar chart of the proportion of full-length and partial reads from PolyA cDNA-sequencing shows large differences between the various library-preparation and sequencing methods. All of the PacBio methods and the Cap-selected ONT approach resulted in a higher percentage of full-length reads. The weakest ratio of complete/incomplete reads are from MinION 1D sequencing. The explanation of this result is the lack of size-selection. In PacBio sequencing, even in the non-size-selected samples the short RNA fragments were eliminated by MagBead loading protocol.

B. The horizontal bar graph shows the proportion of full-length/partial reads derived from oligo(d)T-primed, non-size-selected cDNA sequencing, generated by the three different library preparation kits utilized in this study (the same kits were used for PacBio RSII and Sequel libraries). The sum of the read counts was taken from individual time points of Sequel and MinION 1D sequencing. In order to obtain a full set of transcripts, we mixed RNA samples obtained from various time points for the Cap-Seq analysis. No significant difference between the Sequel and the Cap-selected MinION libraries can be observed, while the MinION 1D-Seq produced much fewer complete sequencing reads.

C. This figure shows the methods that generated a very low amount of complete reads (<10%). The weak result of the non-size selected RSII is not to be considered significant because of the very low yield of this run. However, due to technical reasons, this phenomenon is to be expected from the dRNA-Seq and from the random primed sequencing.

Figure 2. Flowchart diagram shows an overview of the experimental design

Figure 3. Detailed layout of the PacBio wet-lab experiments

Figure 4. Comprehensive experimental workflow of the MinION sequencing

Figure 5. Representation of the depth of viral read coverages generated from different LRS techniques.

A. Circos plot showing the genome-wide transcriptome profile of VACV. The colored boxes represent the genes belong to different kinetic classes [red: early 1 (early); green: early 2 (early-late); yellow: postreplicative (late); grey: unknown)], [13]. Data derived from the five different library preparation and sequencing methods used in this study are shown on the histogram as follows: green: Sequel all data (data from different time points are mixed together); blue: RSII mixed sample; yellow: MinION 1D cDNA mixed sample; orange: MinION Cap-selected mixed sample; black: MinION 1D cDNA barcoded all data (data from different time points are mixed together).

B. Visualization of reads coverage on VACV genome at individual time points. Six time points that were sequenced by PacBio Sequel (inner radius) and ONT MinION (outer radius) have been visualized in a segmented circos plot (every segment represents an individual time point).

C. Sashimi plot presentation of the dRNA-Seq data across the VACV genome.

Figure 6. Polar plot representation of the percentages of virus-host read counts (including the detailed information).

Figure 7. Box plot presentation of the average of aligned read-lengths obtained from the applied sequencing methods. The reads were mapped to the VACV and to the host genome, and the average lengths were plotted with the standard deviation (SD) values.

Figure 8. Comparison of the read-lengths mapped to the host genome of this and other studies. It must be noted here that the analyzed cell lines are from different organisms and/or they were infected with different viruses using different incubation time points.

A-C. RSII and Sequel platforms provide relatively fix read-length (RSII: 800-1,400bp, Sequel: 1,050-1,500bp). The average read-length of CV-1 samples are longer than those of the MRC-5 in the

RSII PolyA-sequencing, however, the opposite result has been obtained with the random-primed RSII and the Sequel PolyA-Seq.

D-G: The MinION platform produces greater length variance (250-1,200bp), except the Cap-Seq approach which shows very small difference between the read-lengths among the four different cell lines.

Cell lines: African green monkey kidney fibroblast cells (CV-1) infected with VACV or Herpes simplex virus type 1 (HSV-1); human lung fibroblast cells (MRC-5) infected with Human cytomegalovirus (HCMV) or Varicella-zoster virus (VZV); Porcine kidney 15 cell line infected with Pseudorabies virus (PRV); and Sf9 insect cell line infected with the baculovirus Autographa californica multiple nucleopolyhedrovirus (AcMNPV).

Figure 9. Bar chart representations of read-lengths distributions (depicted for 500bp long bins, at log10 scale).

A. Sequel. Most of the reads fall within the range of 501-1,000bp at each time point! There are no substantial length differences between the samples within the first four intervals, however the earlier time points disappear later: only the samples from 4h, 6h, 8h and 12h contain reads longer than 4,000bp, while reads longer than 4,500bp could be detected only within the 8h post-infection samples.

B. MinION. Most of the reads falls to the shortest range (1-500bp), and very few reads are longer than 3,501bp from the 4, 6, 8 and 12h samples.

C. RSII size-selected samples. The shortest and longest reads are overrepresented in the 0.8-5kb+ sample. The 0.8-2kb sample represents the shortest read population: no reads are longer than 2kb. There is no significant difference between the samples at the size ranges 2-3kb, 3-5kb and 5kb+; the highest amount of transcripts is within the 501-1,000bp range. The reason of the relatively low read count within the higher size ranges may be that the length of the VACV transcripts are much shorter than e.g. herpesviruses or baculoviruses.

D. RSII no size selected PolyA vs. random-primed samples: the shorter reads are overrepresented in the random primed sample (< 1,000bp); while most of the reads from the PolyA-Seq sample fall within the 1,001-2,000bp interval (this is the typical average read-lengths of the PacBio RSII without size selection).

Figure 10. Comparison of the read-length distributions between the VACV and the host (*Chlorocebus sabaeu*s) transcripts within the utilized non-size selected library preparation methods. Mapped read-lengths are expressed in base pairs and the distribution is showed for 100bp long bins. The x axis is only presented up to 4,000 base pairs, even though the longest read that was detected was up to as long as 9,000bp. 99.86% of the alignments fall into this range. In most cases, the PacBio platforms generated longer reads than the ONT methods.

Figure 11. Illustration of the read-length distributions of the VACV and the host (*Chlorocebus sabaeus*) transcripts within the utilized size-selected library preparation methods. Aligned read-lengths are shown in base pairs per 100bp intervals. The distribution patterns of the viral and host cell reads resemble one another in the size-selected RSII samples, especially in the 2-3kb, 3-5kb and 5kb+ samples. Samples reach their highest peaks around 1,000bp; however, the peak shifts to the right according to the size selection. There is a significant peak in every sample within the shortest range (1-100bp) in the host reads. The effect of size-selection is the most dramatic in the MinION virus sample: the read counts drastically increase beyond 200bp.

Figure 12. Heatmaps depict the relative expression values (the proportion of read counts to the total number of reads at a given time point). The dynamic profiles of the examined viral genes differ in the two datasets derived from the different sequencing methods. This alteration can be explained by the different read-size preferences of the two methods (however, further data analysis is required for accurate kinetic findings). According to the previous studies [13], the examined viral genes belong to the early kinetic class. This is evidenced by the fact that the relative expression values are higher at the early time points – especially in the MinION dataset. The majority of the examined cellular genes

show constant expression level (mainly in MinION data), most of them belong to the housekeeping genes: https://hpcwebapps.cit.nih.gov/ESBL/Database/NephronRNAseq/Housekeeping_Genes.html

The expression patterns of the following genes were analyzed:

VACV

1. *c11r*: Epidermal growth factor-like protein (EGF-like protein)

2. *c7l*: Interferon antagonist C7 (host range protein 2)

n2l: protein N2; m2l: protein M2

e3l: protein E3

h5r: Late transcription elongation factor H5

b8r: Soluble interferon gamma receptor B8

b19r: Ankyrin repeat protein B19

vacwr_4: Truncated CrmB protein

HOST

CST3: *C. sabaeus* cystatin C (XM_007961908.1)

PSAP: *C. sabaeus* prosaposin, transcript variant X1 (XM_007963126.1)

PKM: *C. sabaeus* pyruvate kinase PKM (LOC103217002) (XM_007964863.1)

GAPDH: *C. sabaeus* glyceraldehyde-3-phosphate dehydrogenase (XM_007967342.1)

ENO1: *C. sabaeus* enolase 1, (alpha), (XM_007980661.1)

FTL: *C. sabaeus* ferritin, light polypeptide (XM_007997480.1)

ATP5B: *C. sabaeus* ATP synthase, H+ transporting, mitochondrial F1 complex, beta polypeptide (XM_008003700.1)

ACTG1: *C. sabaeus* actin, gamma 1 (XM_008013242.1)

EEf1A1: *C. sabaeus* eukaryotic translation elongation factor 1 alpha 1 transcript variant X1 (XM_008013483.1)

60S: *C. sabaeus* 60S ribosomal protein L3-like (LOC103247496), mRNA (XM_008019639.1)]

Tables

Table 1. Summary table of the different wet lab approaches applied in this study.

The dynamic transcriptome includes transcripts from various stages of viral infection (from 1h to 8h for Sequel and from 1h to 12h for MinION sequencing), while static transcriptome contain transcripts expressed at various time points of infection.

Table 2. Summary table of the amount of RNA, cDNA and library samples used for PacBio Sequel sequencing: A: amount of PolyA(+) RNA used for cDNA preparation. B: concentration of obtained PCR products. *One µg total RNA was subjected to ribodepletion. C: concentration of SMRTbell libraries.

Table 3. The list of primers sequences used in this study for the reverse transcription reactions. The table also contain the sequence of the gene-specific primer pair used for the amplification of D1R gene of VACV, as well as the sequencing adapters and barcodes.

Table 4. Summary table of the amount of RNA, cDNA and library samples used for ONT MinION sequencing.

Table 5. Summary statistics of the sequencing reads which mapped to the viral genome (A) and to the host reference genome (B) from each run. SE: standard error. * The difference between the yield of the size-selected and non-size-selected (labeled by the asterisk) samples might be caused by the underloading of the SMRT Cell and it is independent from the size-selection step. In some cases, PacBio run results in low output, for which the possible reason is the underloading of the Cells.

Table 6. Statistics of the read counts mapped to the host genome versus host mRNAs.

Additional Files

Additional file 1. Summary table of the reagents and chemistries used for the sequencing.

Additional file 2. Summary statistics of the viral and host reads from each run.

Additional file 3. Read-length distribution is depicted for 100bp long intervals.

Additional file 4. Correspondence between the file names of alignments deposited in ENA and the names that are used in this manuscript. The table also contains the ENA accession numbers of the study, of the experiments, samples, as well as the runs.

Figure 1

Click here to access/download;Figure;Fig1.pdf

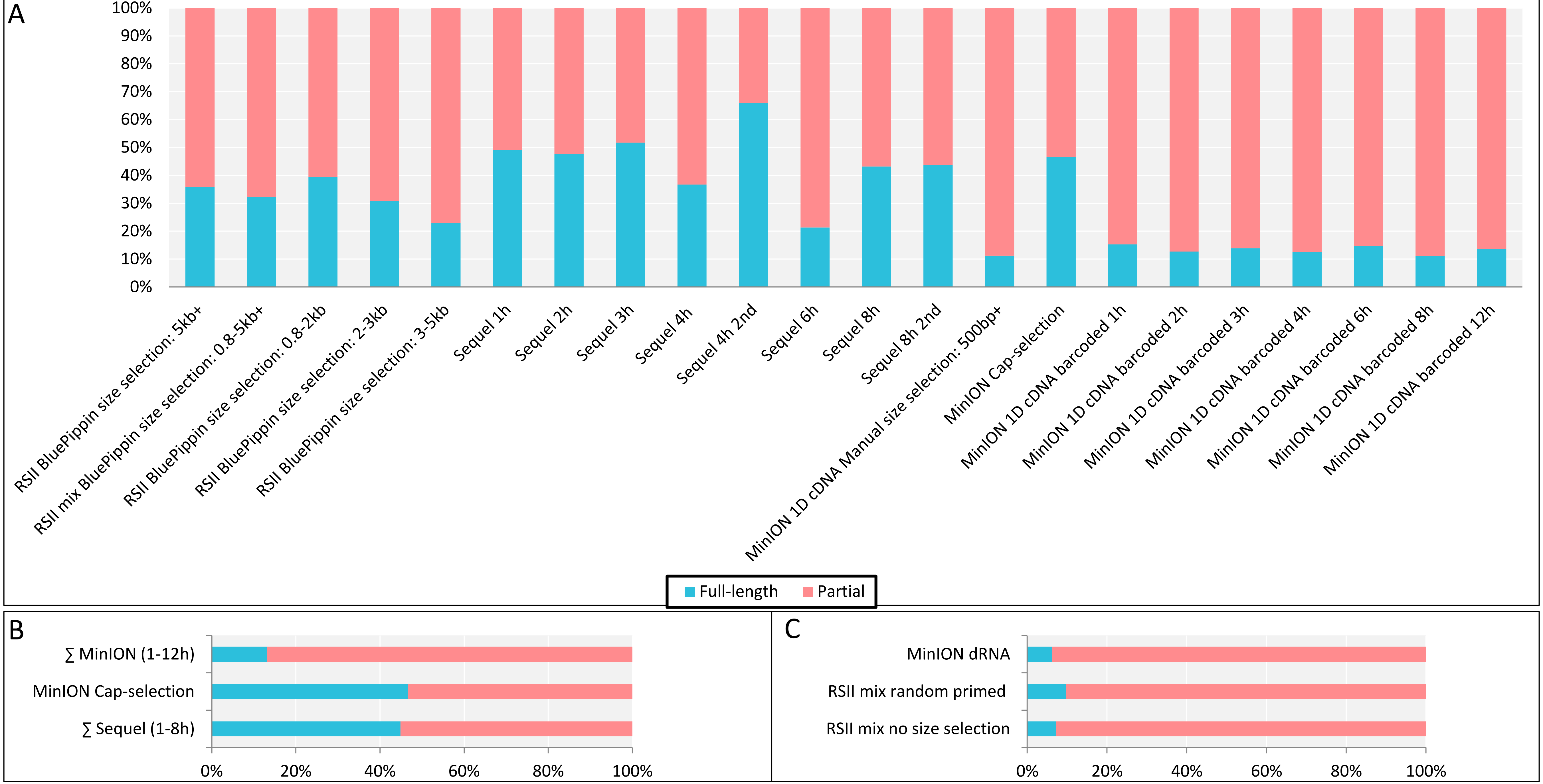

Figure 2

Click here to access/download;Figure;Fig2.pdf

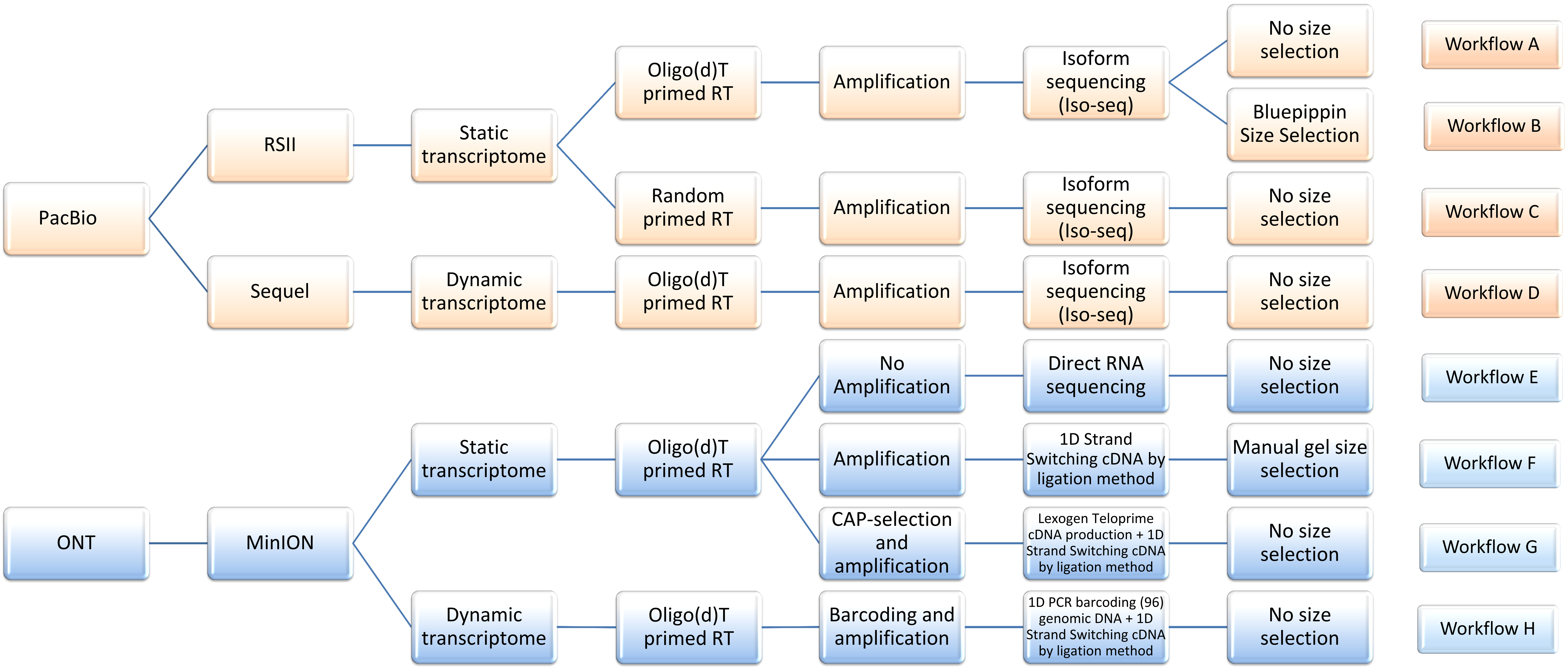

Click here to access/download;Figure;Fig3.pdf

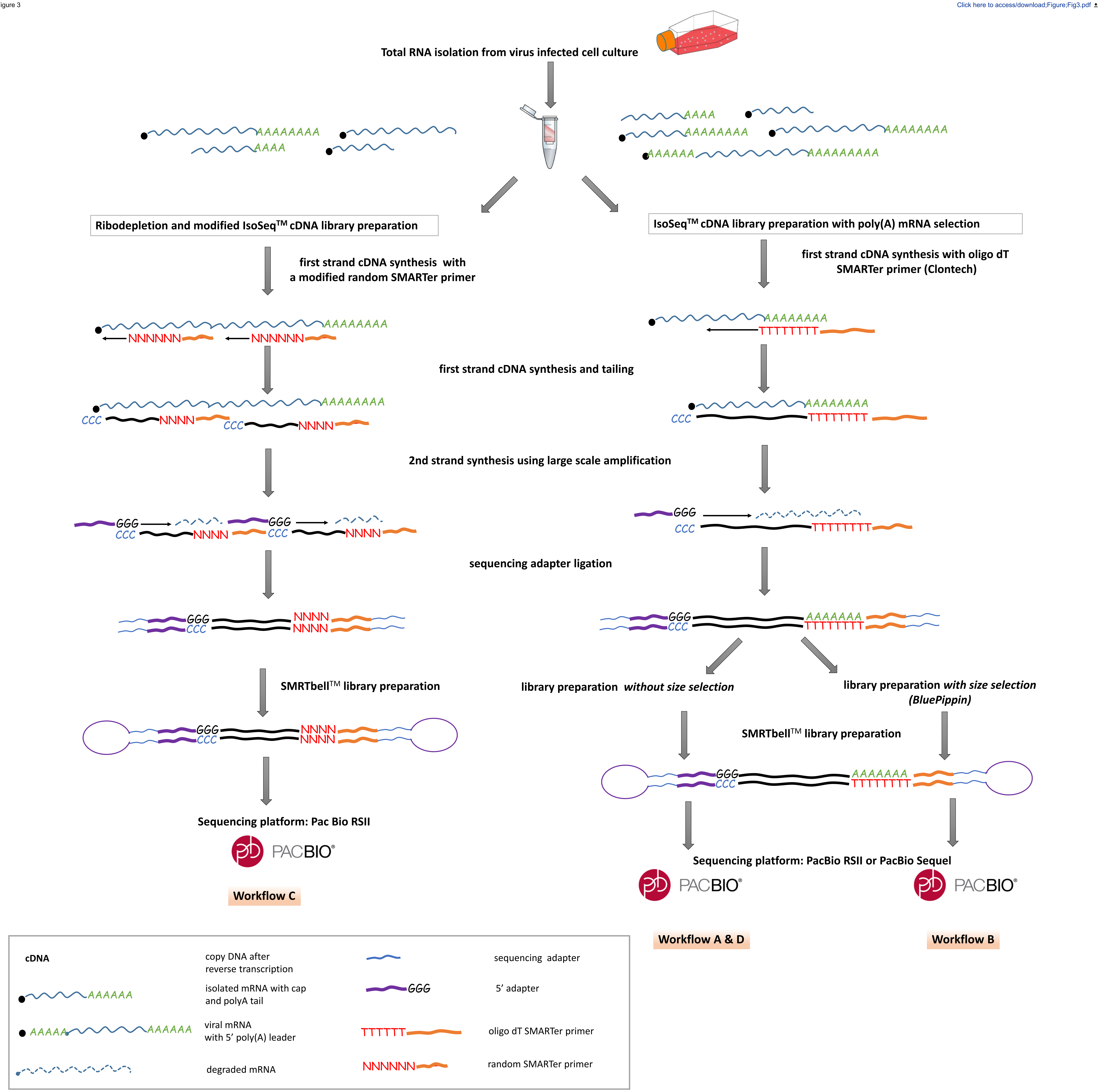

Click here to access/download;Figure;Fig4.pdf

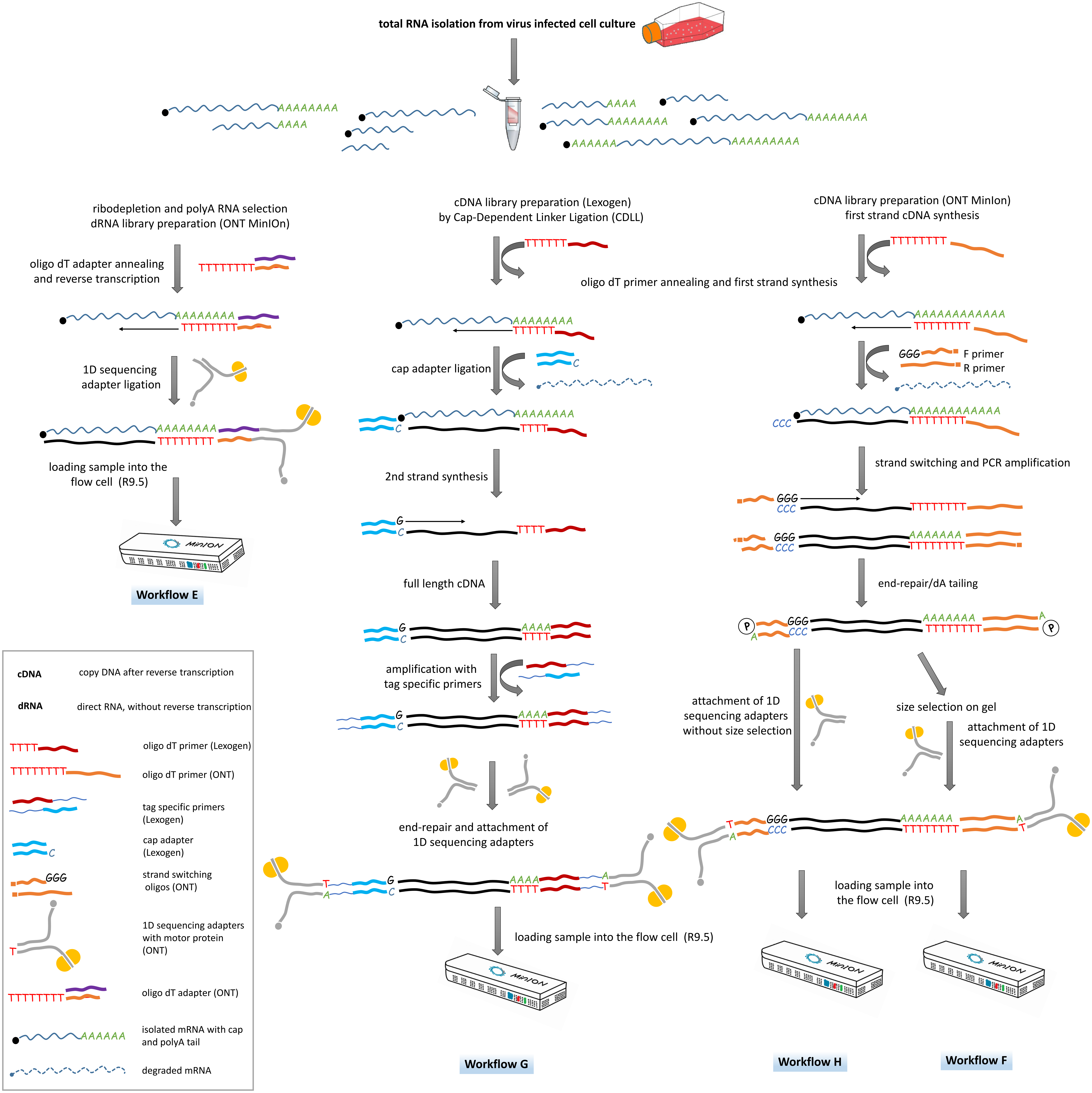

Figure 5

Click here to access/download;Figure;Fig5.pdf

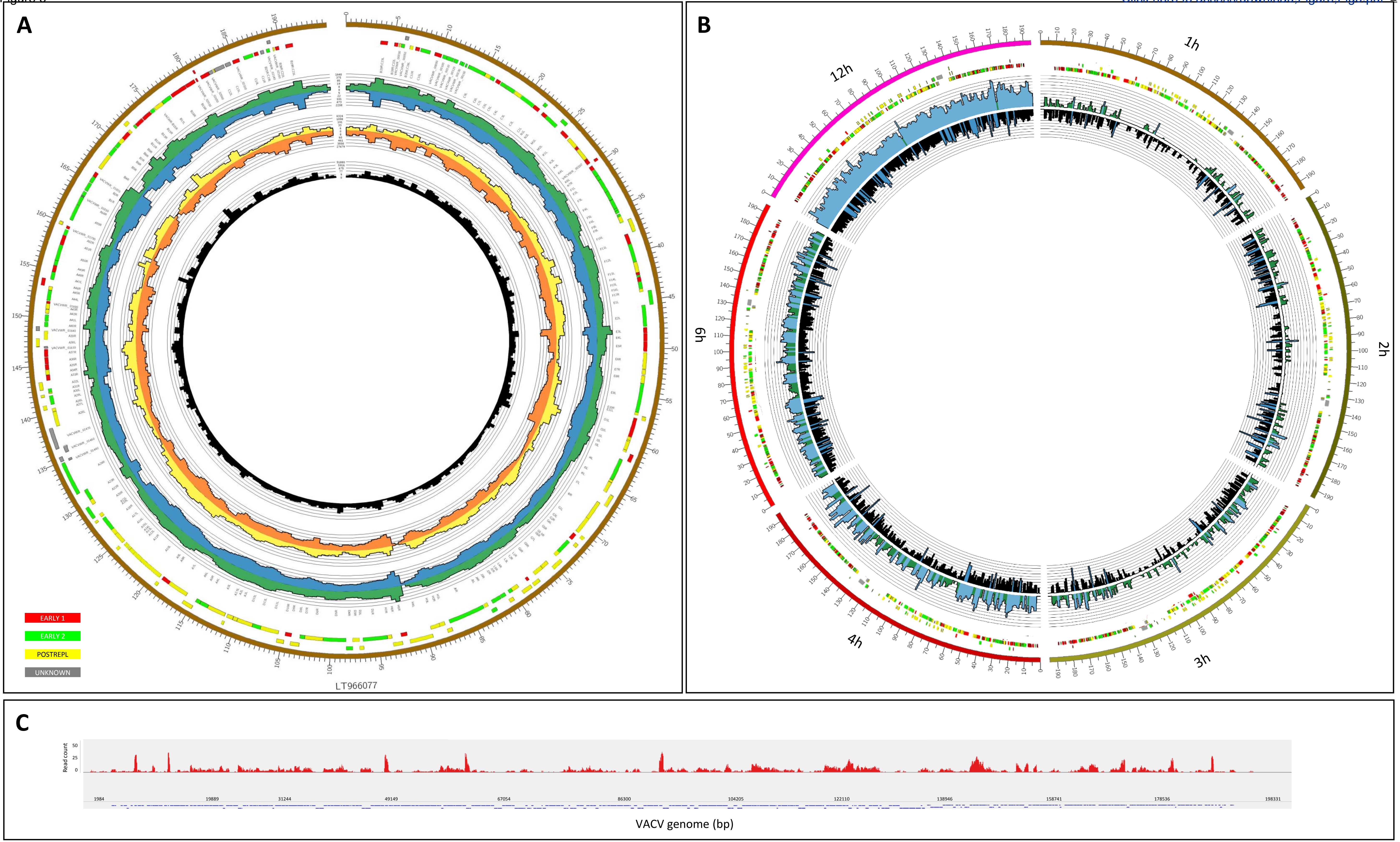

Figure 6

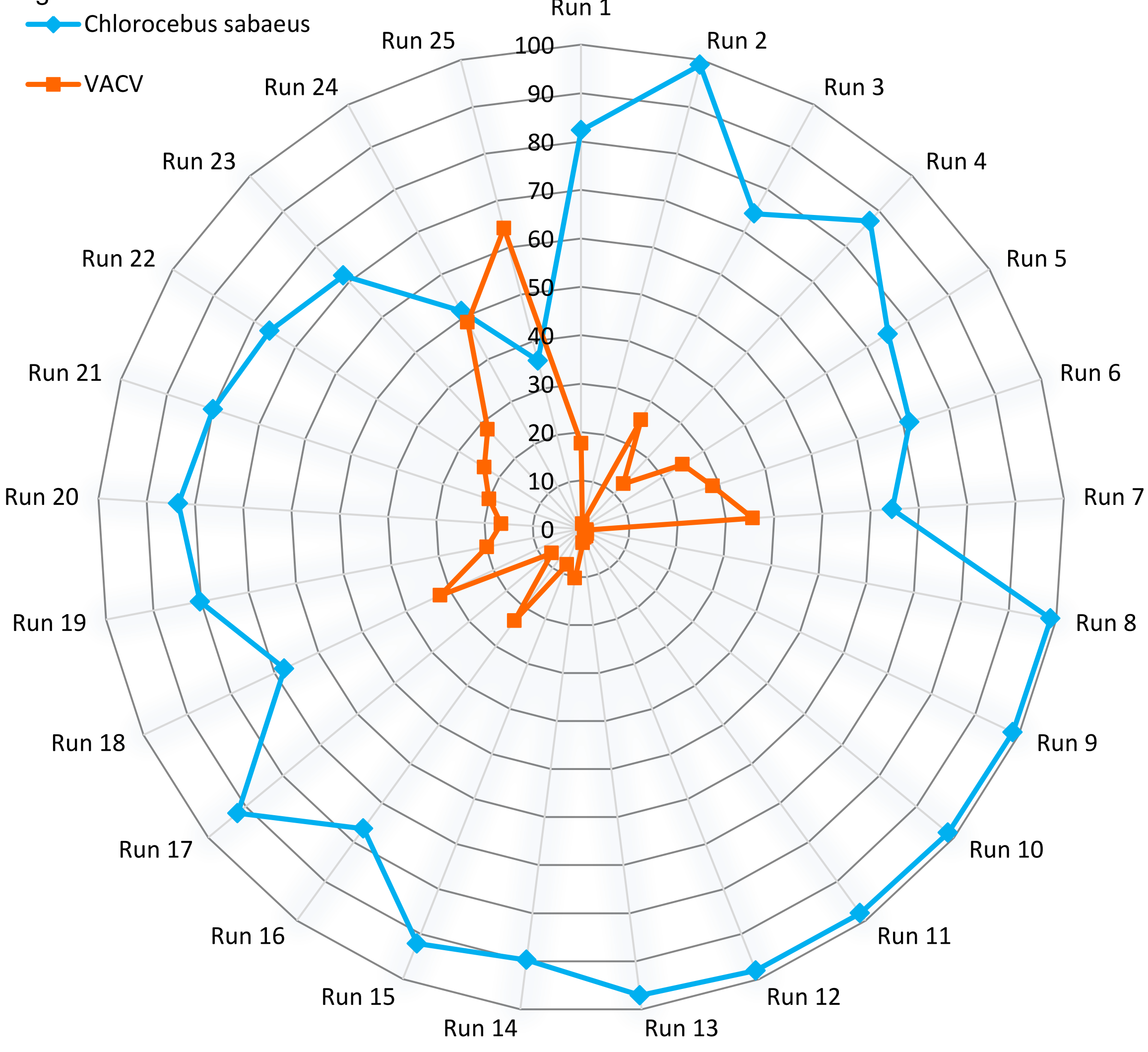

Click here to access/download;Figure;Fig6.pdf

Run #	Samples specificities	Chlorocebus sabaeus	VACV
Run 1	RSII mix no size selection	82.31	17.68
Run 2	RSII mix random primed	98.94	1.055
Run 3	RSII mix BluePippin size selection: 0.8-5kb+	74.28	25.71
Run 4	RSII BluePippin size selection: 0.8-2kb	87.21	12.78
Run 5	RSII BluePippin size selection: 2-3kb	75.14	24.85
Run 6	RSII BluePippin size selection: 3-5kb	71.40	28.59
Run 7	RSII BluePippin size selection: 5kb+	64.42	35.57
Run 8	Sequel 1h	98.82	1.175
Run 9	Sequel 2h	98.64	1.358
Run 10	Sequel 3h	98.46	1.535
Run 11	Sequel 4h	98.12	1.873
Run 12	Sequel 4h 2nd	98.10	1.898
Run 13	Sequel 6h	97.10	2.892
Run 14	Sequel 8h	89.73	10.26
Run 15	Sequel 8h 2nd	92.10	7.891
Run 16	MinION 1D cDNA Manual size selection: 500bp+	76.54	23.45
Run 17	MinION dRNA	92.13	7.860
Run 18	MinION Cap-selection	67.78	32.21
Run 19	MinION 1D cDNA barcoded 1h	80.20	19.79
Run 20	MinION 1D cDNA barcoded 2h	83.43	16.56
Run 21	MinION 1D cDNA barcoded 3h	79.99	20.00
Run 22	MinION 1D cDNA barcoded 4h	76.27	23.72
Run 23	MinION 1D cDNA barcoded 6h	71.77	28.22
Run 24	MinION 1D cDNA barcoded 8h	51.32	48.67
Run 25	MinION 1D cDNA barcoded 12h	35.88	64.11

Figure 7

Click here to access/download;Figure;Fig7.pdf

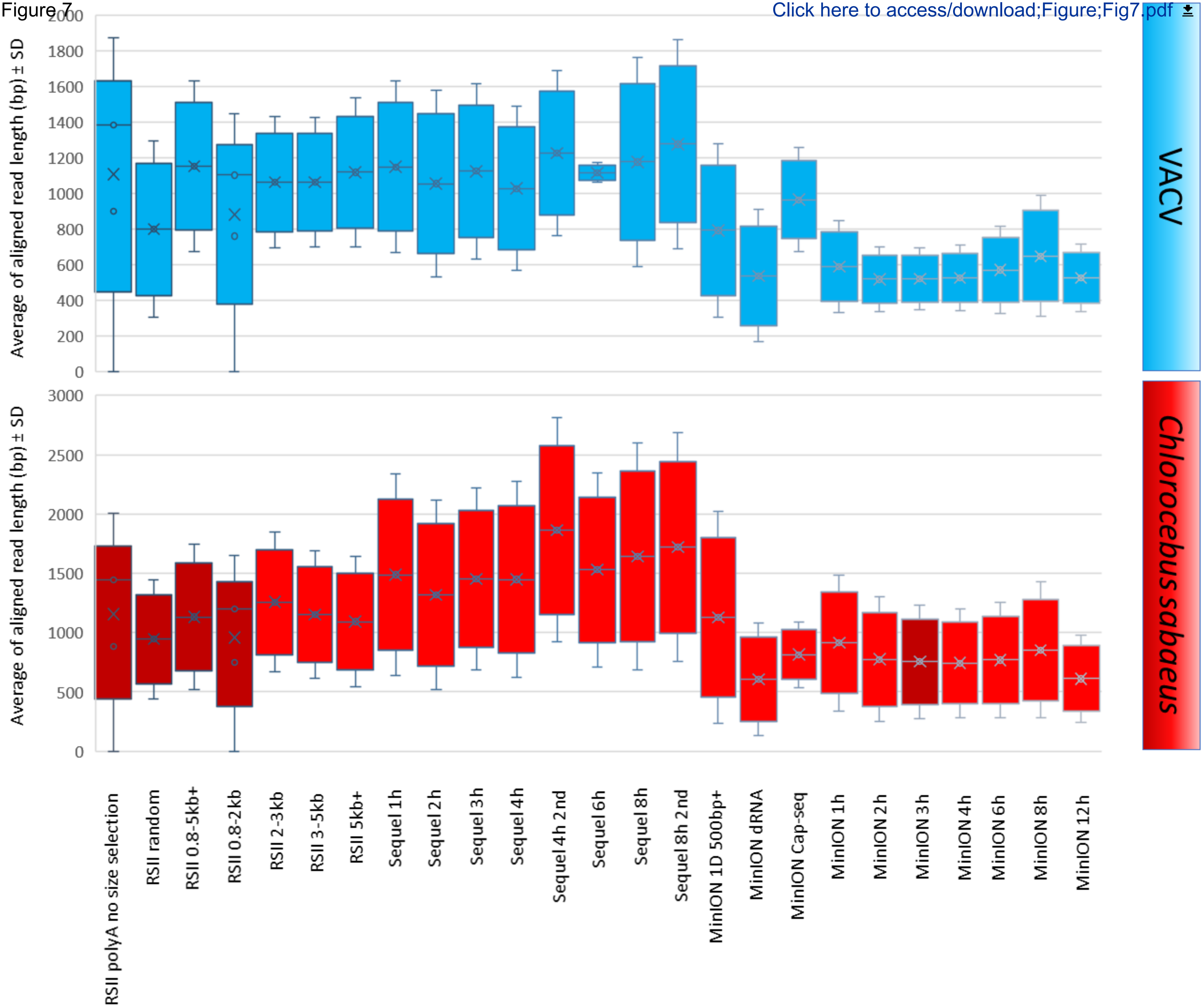

Figure 8

Click here to access/download;Figure;Fig8.pdf

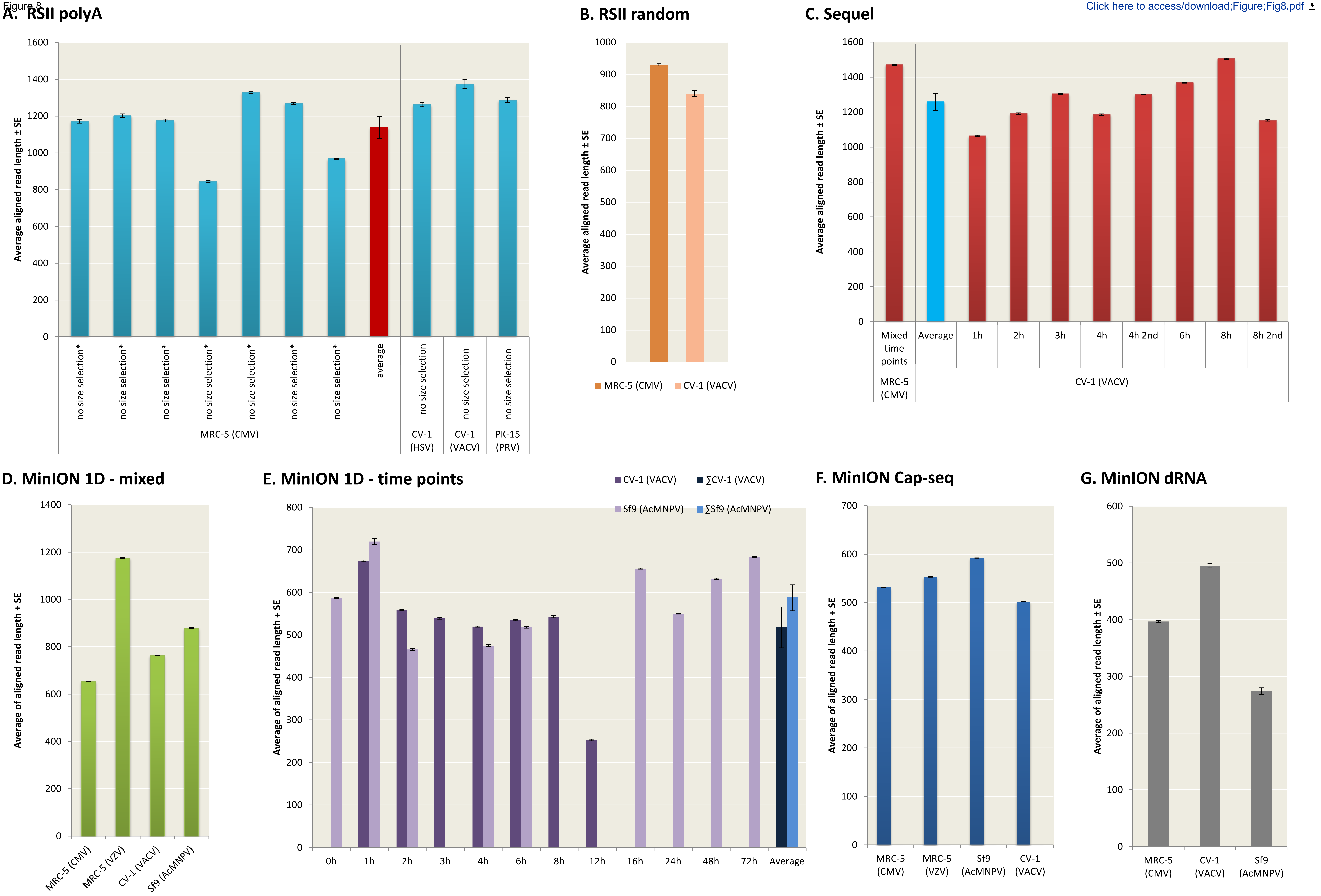

Click here to access/download;Figure;Fig9.pdf

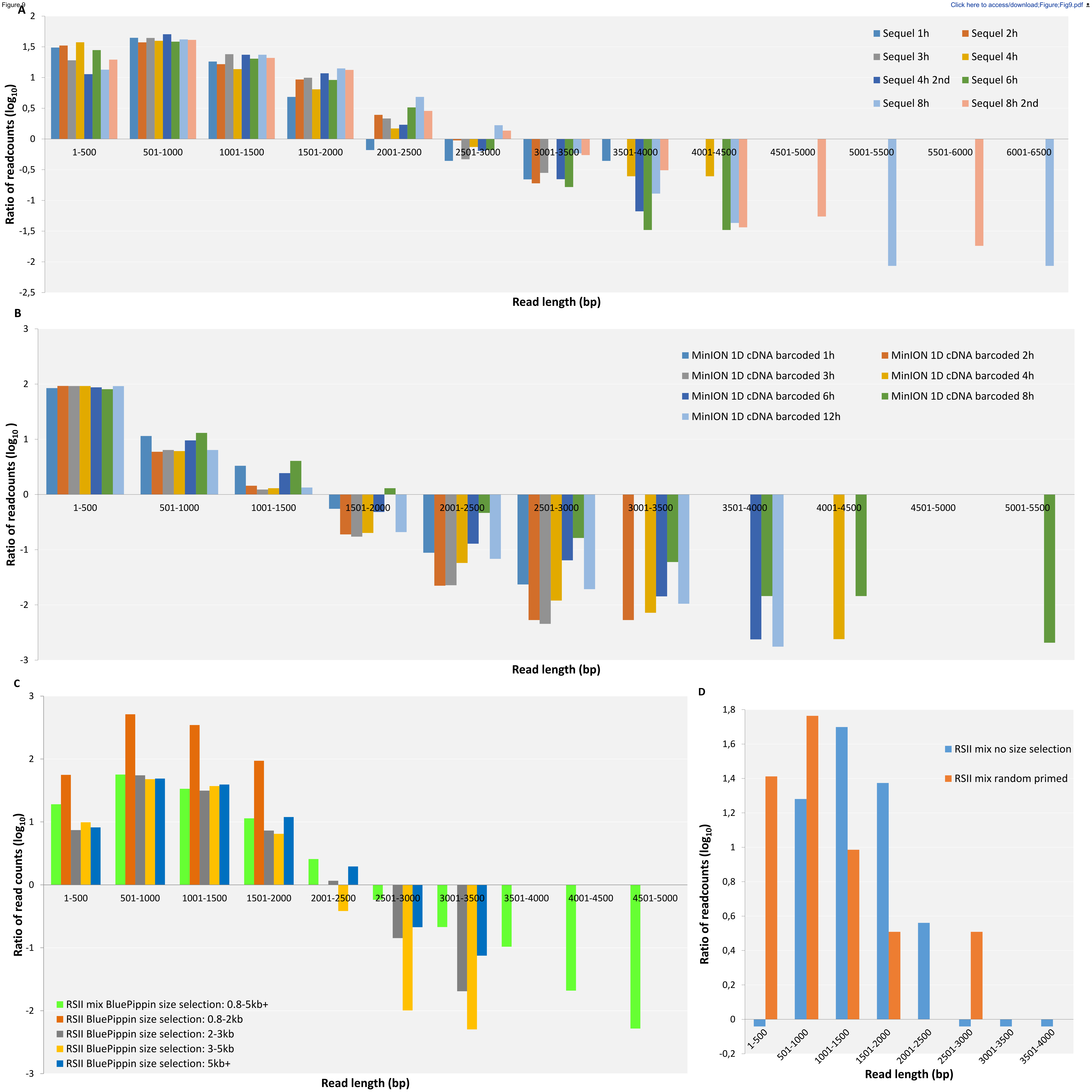

Figure 10

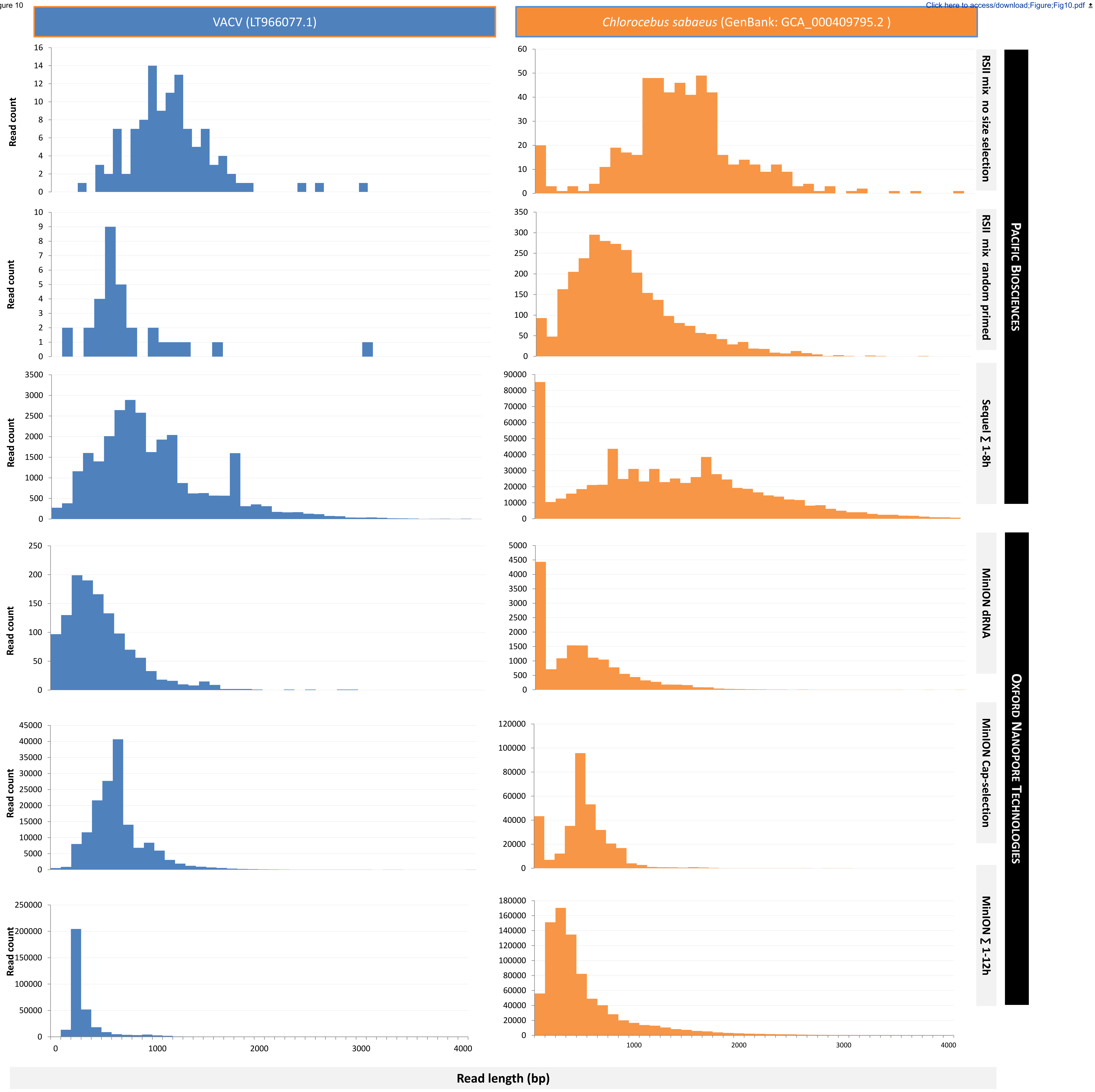

VACV (LT966077.1)
Chlorocebus sabaeus (GenBank: GCA_000409795.2)
RSII mix no size selection
RSII mix random primed
Sequel Σ 1-8h
MinION dRNA
MinION Cap-selection
MinION Σ 1-12h
PACIFIC BIOSCIENCES
OXFORD NANOPORE TECHNOLOGIES
Read count
Read length (bp)

Figure 11

Click here to access/download;Figure;Fig11.pdf

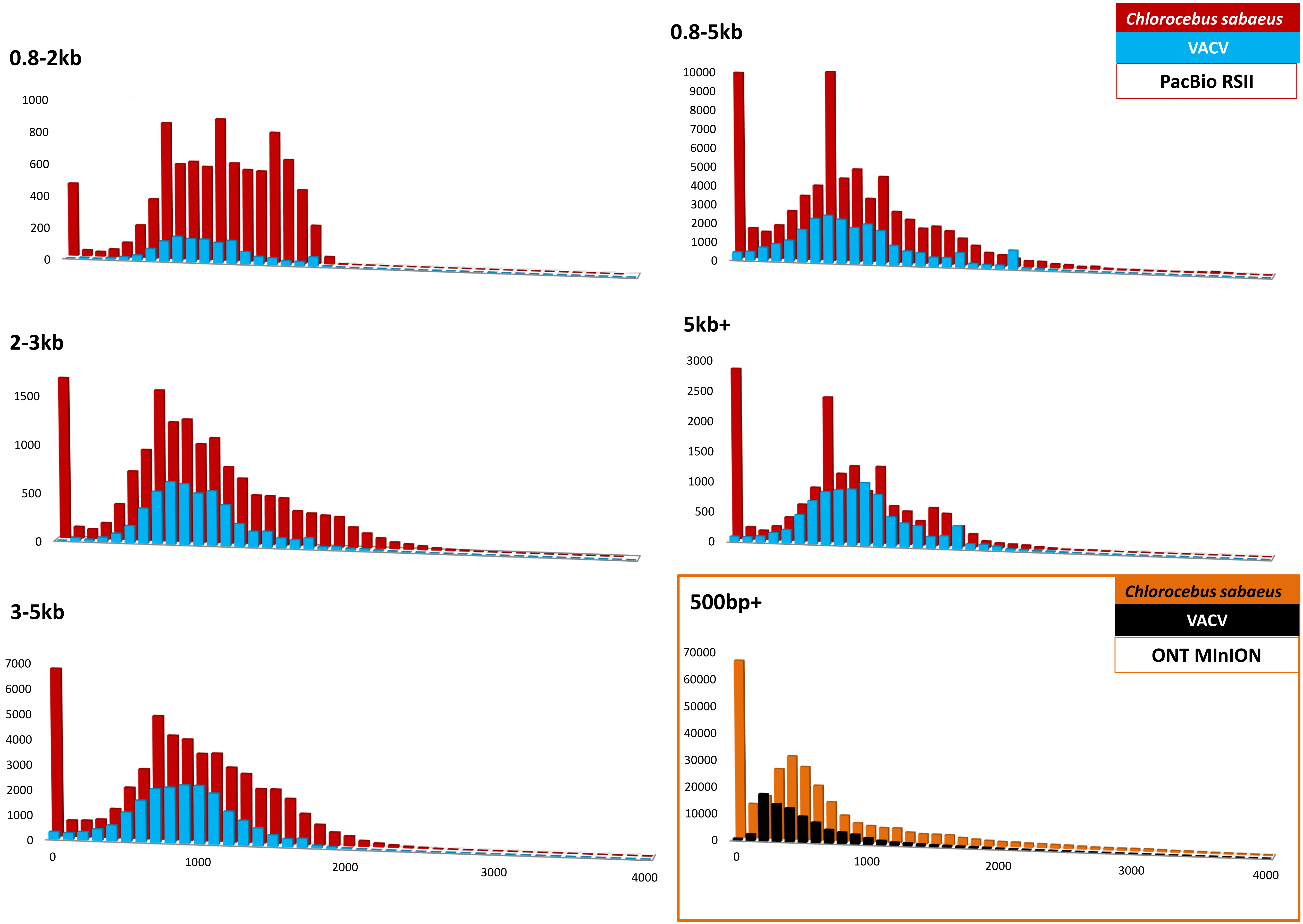

Figure 12

Click here to access/download;Figure;Fig12.pdf

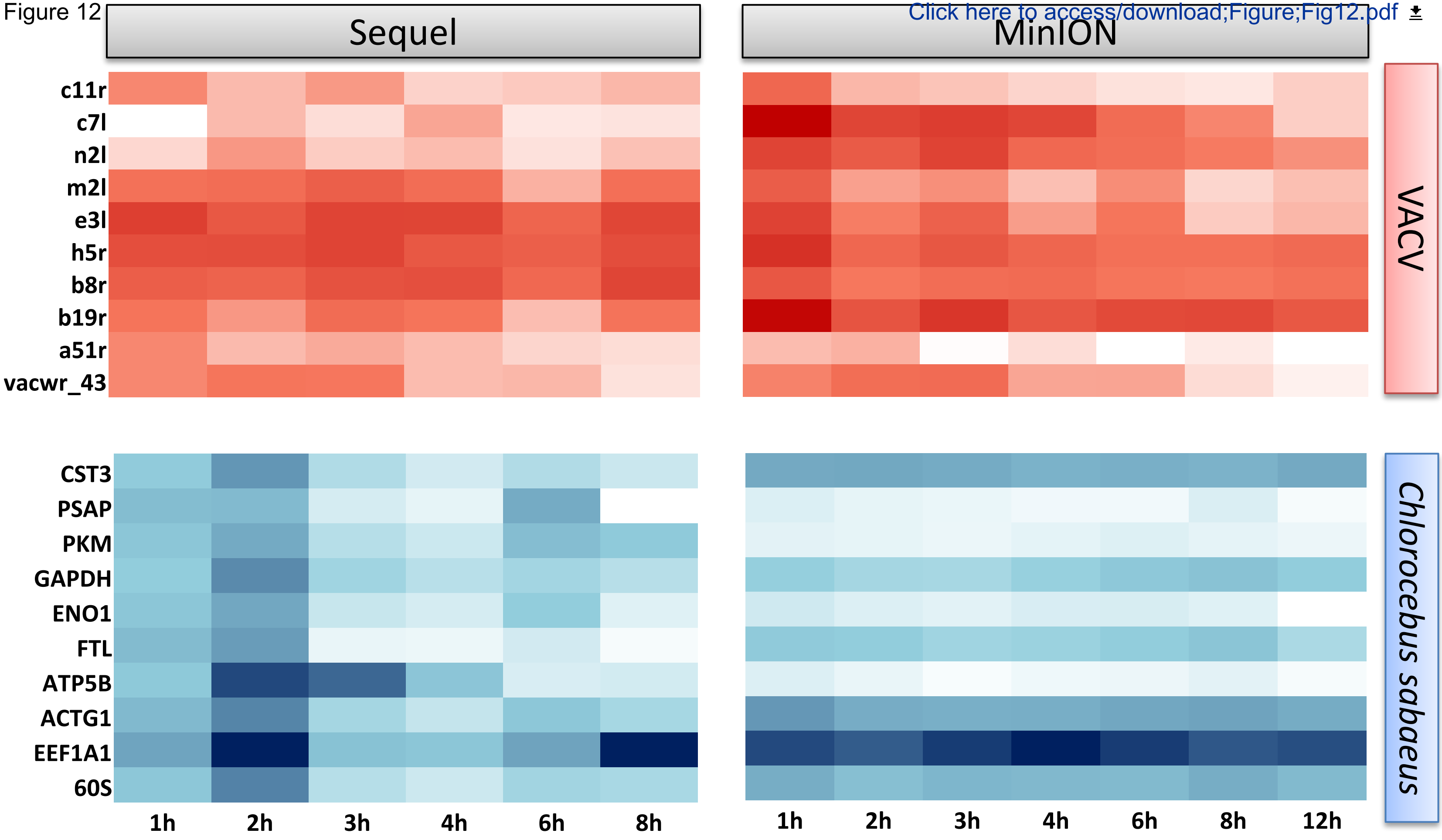

Additional file 1

Click here to access/download
Supplementary Material
Additional_file1.xlsx

Additional file 2

Click here to access/download
Supplementary Material
Additional_file2.xlsx

Additional file 3

Click here to access/download
Supplementary Material
Additional_file3.xlsx

Additional file 4

Click here to access/download

Supplementary Material

Additional_file4.xlsx

Cover letter

Click here to access/download;Personal Cover;Answers to the reviewers.docx

Dear Editor,

We would like to thank you and the three referees for the helpful comments and suggestions to our manuscript. We have made the recommended corrections and we hope that the manuscript will now be acceptable for publication in *GigaScience*. The changes have been marked in blue in the revised version of the manuscript. Our point-by-point responses to the comments are outlined below.

Reviewer #1:

Tombacz et al. used multiple long-read sequencing techniques to sequence VACV and its host cell transcripts during infection. This study obtained over one million reads. Full-length long deep-sequencing reads are critically needed in poxvirus research due to the complexity of poxvirus transcripts: pervasive transcription initiation, termination, extensive read-throughs, tightly spaced ORFs et al. Some of these issues could not be adequately addressed using sequencing technologies yielding relatively short reads. While these data likely provide useful dataset for poxvirus study, more information is required to access the quality of the data and its utilities.

Major points

1. *Based on the information provided, it is not clear whether the reads (or what percentage of the reads) are full-length transcripts (although the authors claimed that they obtained full-length reads in line 91). Especially, the lengths from different sequencing platforms varied greatly. If there are only a very small portions of the reads are full-length transcripts, the advance of this study is limited as compared to those earlier studies.*

We have added this information to the text of the revised version of the manuscript. Furthermore, a new figure (Figure 1) has also been added to the manuscript.

2. *Read length distribution analysis, in addition to the average/median read lengths, will be informative to access the quality of the reads.*

We have added this information to the text. Three new figures (Figures 9, 10 and 11) have been added to the manuscript. The read-length distributions for the dataset are shown in Figure 9 (reads mapped to the VACV genome), as well as Figures 10 and 11 (data aligned to the VACV and to the host genome).

3. *Please discuss different read lengths using different sequencing platforms. They are unlikely all full-length transcripts. What are the advantages and disadvantages in poxvirus transcriptome analyses of the reads from different sequencing platforms?*

We have added this information to the text of the revised version of the manuscript. The various sample preparation and sequencing techniques produce different read-length, read number and precision. Additionally, the various techniques produce different artifacts. There is a relatively large difference between the PacBio and ONT sequencing approaches concerning the quality of the sequencing reads: PacBio produces much fewer mismatches and INDELs than nanopore sequencing. However, high quality reads are unnecessary for transcriptome studies if the genome of the organism is known. The various sequencing platforms recommend different cDNA production kits, which contain different enzymes and

primers for the RT and PCR. The different primers and library preparation conditions may produce different artifacts; however, these can be easily filtered out if we compare the results of different methods. The PacBio MagBead loading selectively eliminates the short fragments (<1,000bp). While on the one hand, removal of incomplete cDNAs can be advantageous, at the same time, it is unfavorable, as we are unable to detect the shorter transcripts and RNA isoforms.

***4.** How is the average length of cellular transcripts in this study as compared to those in other studies?*

A new figure (Figure 8) has been added to the manuscript. In this figure, we have compared the average aligned read-length of cellular transcripts obtained in this and other studies.

***5.** Specifically evaluate the reads (length, coverage, full-length or not, coverage in coding and non-coding regions, et al) of a few VACV ORFs and cellular genes with high resolution at different replication stages will help access the quality of the dataset.*

A new table (Table 6) has been added to the manuscript. We have also added a heatmap illustration (Figure 12) on the expression dynamics of ten viral and ten host-specific genes.

***6.** What are the percentages of viral and cellular reads at each time point?*

We have provided this information in the revised manuscript (see Figure 6).

***7.** Fig. 4. Visualization of reads coverage on VACV genome at individual time points is needed to access the quality of reads at different stages of VACV replication.*

We have added an additional figure panel (Figure 5B) that presents the individual time points from Sequel and MinION sequencing. We have also retained the previous figure as Figure 5A, which includes the five different platforms and/or cDNA library preparation approaches. There are no individual time points from RSII and MinION Cap-Seq, and there is also an RSII dataset from mixed time points samples.

***8.** Sequencing error rates were not discussed in the manuscript.*

We have added this information to the text.

Minor points.

***1.** Lines 49-50, is VACV a cowpox virus? From what I understand, it is not clear.*

Yes, the VACV is the cowpox. We have modified the text for better clarification.

***2.** Table 1, what is Polya(A)(+) in the RNA sample column? Poly(A)(+)?*

Yes, it is Poly(A)(+). We have corrected the misspellings.

3. *Please correct some errors or typos throughout the manuscript. For example, line 116, PSB -PBS. Line 99, A detailed workflow-detailed workflows.*

We have corrected the errors and typos.

4. *Fig 5. 6. 7, Please label Y-axis.*

Figure 5 and 6 have been changed to box plot and the Y-axis has been labeled, but Figure 7 has been removed as recommended by Reviewer #2.

Reviewer #2:

In the manuscript "Dynamic Transcriptome Profiling Dataset of Vaccinia Virus Obtained from Long-read Sequencing Techniques" by Tombácz et al., the authors describe a dataset produced by Pacbio and Nanopore sequencing of VACV, with multiple approaches taken in both sample collection and sequencing library preparation in order to profile several features of the transcriptome. The dataset is of obvious importance to the field and contains many interesting features, but there are some concerns which would need to be addressed before this manuscript is ready for publication. Specific points:

1. *It would be beneficial to include a brief statement defining "dynamic" and "static" sample collection strategies in your data description to clarify how you are using these terms. It is unclear what infection condition means in the Table 1 context, and should either be clarified or replaced by something along the lines of "sample collection strategy".*

We have defined the terms 'static' and 'dynamic' in the Table legend, and replaced 'infection condition' with 'sample collection strategy' as suggested.

2. *How do the samples in Table 2 (A, B, C) correlate with the sequencing run in Table 1?*

An additional column has been added to Table 2. Furthermore, details from RSII sequencing have also been added.

3. *Why was yield so high for the viral genome given the lack of selection? Is the level of viral transcript typically this high, and what you would expect?*

We have developed a novel program recently published on Github, which was used for the reanalysis of our data in the revised manuscript: https://github.com/Szunyike/SAM-Statistic-2018

The ratio of viral transcripts is 21.9% on average in our samples. The exact ratio is dependent on the titer of the virus used for the infection, as well as on the stage of the viral life cycle at the examination period. The sequencing method affects the ratio of read counts between the virus and host cell: e.g. the MinION 1D-Seq method yields a higher amount of shorter reads compared to the PacBio Sequel technique. The VACV transcripts are relatively short compared to the host or to other large DNA viruses (such as herpesviruses and baculoviruses), which is assumed to result in the relatively high ratio of viral reads compared to the host reads in the MinION samples.

4. *Tables 6 and 7 should be combined for ease of interpretation. Tables are not as useful to show read/insert length distribution as actually plotting the distribution, and box and whiskers are inadequate. We recommend pulling out error rates, and representing instead as violin plots, or at least box plots, and include a column for "total reads" and either the count of mapped VACV reads, or percent reads mapped to VACV. Similarly, figures 5 and 6 should be combined to allow for direct comparison, and figure 7 should be removed.*

Tables 6 and 7 have been combined into a single table (Table 5). Figures 5 and 6 have been changed to a box plot and they are combined into a single figure (Figure 7). Read-length distribution has also been presented in the revised manuscript (Figures 9, 10, 11 and Additional file 3). Figure 7 of the old version of the manuscript has been removed. We have provided a new figure (Figure 6) showing the percentages of viral and cellular reads at each sample.

5. *Regarding alignments, it is unclear why certain sequencing were aligned to VACV and why others were aligned to both VACV and CV-1. For example, why aren't the RSII and direct RNA runs included in Table 7 and Figure 6 host mappings?*

We have aligned the RSII and dRNA data to the CV-1 cell line and the statistical data has been added to Table 5 (Table 7 in the old version).

6. *On your Circos plot, it would be interesting to color genes by early/intermediate/late stage. Also regarding the Circos plot - where is the dRNA data?*

The Circos plot has been modified according to the recommendation: the various kinetic classes have been labeled in different colors (Figure 5A). Another picture of coverage on VACV genome at individual time points has also been added (Figure 5B) as was suggested by another Reviewer. The coverage in dRNA sequencing is very low compared to the other five (Sequel, RSII, MinION 1D cDNA, MinION 1D barcoded, and MinION Cap) approaches, and it is not visible at the scale applied for the generation of circus plot. Therefore, we have added a Shashimi plot as a part of this figure (Figure 5C) instead of adding the dRNA values to the same circus plot.

7. *In Table 6, your coverage calculations seem improbable in places. For example, how could the RSII 5kb+ BluePippin run with 8 mapped reads have 42.17X coverage?*

We have corrected this mistake.

8. *Your statement about searching for modifications in dRNA should be amended - although 5mC is widespread in DNA, it is not as ubiquitous in RNA. Additionally, 6mA has been most extensively profiled in viruses: http://jvi.asm.org/content/91/9/e02263-16.full, and is actually quite common.*

We have corrected this part of the manuscript.

9. *In the 1D ONT-cDNA sequencing section, instead of telling the reader that different steps were used from the ONT protocol, I suggest describing what enzymes were used for the end-polishing. The ONT protocols are not publicly accessible, and since the preparations done on these samples were so complex, it may make sense to just say what was done.*

We have added detailed information about the ONT 1D cDNA and direct RNA protocols to the revised manuscript.

10. *Why not minimap2 instead of GMAP? Choice of GMAP is perfectly acceptable, but for readers it would help to explain the choice.*

We have chosen GMAP because we have found it the best long-read aligner in our earlier publications [18, 19, 20, 21, 22, 23, 24]. GMAP have also produced the best alignment results in other studies [e.g. 28]. We generated the PacBio RSII dataset in 2016, when Minimap2 had not yet existed. In the beginning, Minimap2 did not support for RNA mapping (version 1: https://arxiv.org/abs/1708.01492v1). Our MinION and Sequel data are newer, and later versions of the Minimap2 program now support RNA mapping. However, our opinion is that the correct approach would be to use the same program for mapping that we had used to attain our existing data. We have added the Minimap2 program as a recommended long-read aligner to the 'Conclusion and Reuse Potential' section of the manuscript.

11. *Language occasionally awkward, for example "VACV remains to be considered a weapon against potential smallpox outbreaks". Careful editing for grammar, subject-verb agreement will greatly improve readability.*

The manuscript has been thoroughly edited in terms of grammar, and was proofread by a native speaker of English.

Some minor points:

1. *Fastqs (unsorted by organism, just of all reads, for each run) should be hosted on gigascience for ease of access - this would be the first piece of information we would want to download.*

We have uploaded the fastq.qz files to FigShare:

https://figshare.com/s/675f5f71c633473b7445

2. *In findings section of the abstract, it is more useful to include the genus/species of host, and leave the accession numbers to the methods section. Additionally, it would be more useful to include Gb data produced, as well as the size of the genome, upfront.*

We have modified the text as recommended.

3. *In table 1, please make yes/no capitalization consistent. A label of some kind linking the workflows in Fig 1 and Fig 2 and 3 - or linking to the Table would be very helpful. Many different preparations are represented here, and parsing them in the current format is challenging at best. Perhaps some sort of encoding like ONT-cDNA-polyA_1 at the bottom of fig 3 for the relevant workflow and labeled as a column in Table 1? Additionally, please make clear for fig 3 what is dRNA and what is cDNA.*

The recommended corrections have been made in Table 1. We have also made some minor modifications on Figures 3 and 4 (Figures 2 and 3 in the old version of the MS) according to the reviewer's suggestion. Abbreviations of dRNA and cDNA are now explained and we have provided a linking label between Figures 2, 3 and 4 (Figure 1, 2 and 3 in the old version of the

manuscript). The labels are as follows: „Workflow A, B, C, D, E, F, G and H”, and can clearly identify the different methods depicted on Figures 3 and 4 (Figures 2 and 3 in the old version of the MS).

4. *Sometimes you denote 5'/3', sometimes 5'/3', please make consistent to 5'/3'.*

We have modified the text with consistent labeling of 5’/3’ throughout the manuscript.

5. *All of your figures should have y-axis labels and, in the case of figures 4 and 7 - a legend explaining the colors.*

The rectangles are colored according to the known kinetic properties of the genes: red: early 1; green: early 2 (early-late); and yellow: postreplicative (late). Y-axis labels have been added to the figures. Figure 7 has been removed as recommended by Reviewer #2

6. *Please add "availability of source code and requirements" as per*

https://academic.oup.com/gigascience/pages/data_note

We have added the required information to the manuscript.

7. *Standard deviance should be changed to standard deviation.*

We have corrected the wording as recommended.

8. *The statement in line 235 "These aligned reads can be further analyzed by using different long read aligners" should be modified to "These aligned reads can be further analyzed by comparing to results of different long read aligners"*

This sentence has been changed as recommended.

9. *Alignment file names should be updated to a consistent format*

A supplementary table (Additional file 4) has been added which explains the file names of alignments deposited in ENA paired with the names that are used in this manuscript.

10. *Tables 3 and 5 should be combined.*

These two tables have been combined. Additional data have also been added.

11. *Table 1 and Figure 1 are redundant, consider removing Figure 1 or adding additional unique information to it.*

We have provided additional information to Figure 2 (Figure 1 in the old version of the MS) according to the reviewer’s recommendation: we have labeled the unique workflows (A, B, C, D, E, F, G and H) on Figure 2, which helps to identify the different methods presented in the Figures 3 and 4 (Figures 2 and 3 in the old version of the manuscript).

12. *Your text mentions a "fast5.tar.gz" file for ONT dRNA, but listed in the dRNA folder of archives is just a "tar.gz" file in addition to the BAMs - please clarify that this is the same. Also, you only included the raw reads which aligned to VCAV - but it is worthwhile to include all reads as well, because your alignments might not be all-inclusive.*

The files Fast5.tar.gz and tar.gz files are the same. The reads aligned to the host genome have been deposited in the ENA.

13. *What size is the tissue culture flask for the CV-1s?*

We used 25 cm^2 culture flasks for the propagation of CV-1 cells. This information has been added to the MS.

14. *Why were 3x freeze-thaw cycles applied? What is the reasoning for that?*

Freezing and thawing were carried out to help the degradation of cell membrane and nuclear envelope for more efficient isolation of RNA molecules.

Third report:

In this Data Note, the authors present full-length transcriptome data generated by third-generation single-molecule long-read sequencing technologies including PacBio (Pacific Biosciences) and ONT (Oxford Nanopore Technologies) for VACV (Vaccinia virus). Two full-length cDNA synthesis methods (Clontech SMARTer and Lexogen TeloPrime) and three platforms (PacBio RS II, PacBio Sequel and ONT MinION) were applied in this study. The authors also generated transcriptome data using ONT direct RNA sequencing technology. These data will benefit the identification of novel VACV RNA isoforms for virologists and the development and evaluation of bioinformatics tools. Below are some comments on this manuscript.

1. *The authors used two strategies ("no size selection" and "Bluepippin Size Selection") for PacBio RS II platform. Why the number of ROIs generated by "no size selection" is significantly smaller than "Bluepippin Size Selection" (Table 6)?*

The size-selected samples do not necessarily produce higher yields than the non-size-selected samples. In some cases, PacBio run results in low output, for example because of underloading of the SMRT Cell. This sample is a minor part of our dataset. We have labeled this sample with an asterisk (*) in Table 5 (Table 6 in the previous version of the MS) and indicated the possible cause for the yield differences in the figure legend.

2. *In Tables 6 and 7, three columns "insertion frequency", "deletion frequency" and "mismatch frequency" are shown, but the authors did not describe them in "Data summary" section. More details should be included.*

We have added more details to the Data summary section of the revised manuscript.

3. *The grammar should be improved. For example, in Page 4 Line 81, "determine which transcripts contains certain 5'-ends and 3'-ends."; in Page 4 Line 86, "By using these techniques for cDNA production and"; Page 7 Line 140, "240 minutes were set for the RSII*

movie lengths, while 600 min were applied"; in Page 10 Line 191-192, "This basecaller is able identify the nucleotide sequences directly from raw sequencing data."

We have corrected the grammar.

4. *In Table 2, the comma should be converted to the dot.*

We have corrected this error.

5. *In Page 6 Line 42-44, to the best of our knowledge, m6A is an abundant modification in mRNA. Could the authors check whether m6A or 5mC is most widespread in RNA modifications? In addition, the abbreviation should be "m6A" for RNA N6-Methyladenosine. The "6mA" is the abbreviation of DNA N6-methyladenine.*

We have modified the appropriate part of the manuscript. We also have added new text and new references to the revised MS. Furthermore, we have changed the abbreviation of N6-Methyladenosine to “m6A”.

6. In Page 15 Line 293, the full name of "TSS" should be "transcription start site".

We have corrected this error.

https://giga.editorialmanager.com/l.asp?i=41827&l=SE8GCNWN
