# Supplementary material for: Dynamic transcriptome profiling dataset of vaccinia virus obtained from long-read sequencing techniques
Source: Gigascience. 2018 Nov 23;7(12):giy139. doi: 10.1093/gigascience/giy139 (PMC6290886; doi:10.1093/gigascience/giy139)
Supplement: response_to_reviewer_comments_original_submission.pdf [file giy139_response_to_reviewer_comments_original_submission.pdf]

Author's Response To Reviewer Comments

Close

Dear Editor,

We would like to thank you and the three referees for the helpful comments and suggestions to our manuscript. We have made the recommended corrections and we hope that the manuscript will now be acceptable for publication in GigaScience. The changes have been marked in blue in the revised version of the manuscript. Our point-by-point responses to the comments are outlined below.

Reviewer #1:

Tombacz et al. used multiple long-read sequencing techniques to sequence VACV and its host cell transcripts during infection. This study obtained over one million reads. Full-length long deep-sequencing reads are critically needed in poxvirus research due to the complexity of poxvirus transcripts: pervasive transcription initiation, termination, extensive read-throughs, tightly spaced ORFs et al. Some of these issues could not be adequately addressed using sequencing technologies yielding relatively short reads. While these data likely provide useful dataset for poxvirus study, more information is required to access the quality of the data and its utilities.

Major points

1. Based on the information provided, it is not clear whether the reads (or what percentage of the reads) are full-length transcripts (although the authors claimed that they obtained full-length reads in line 91). Especially, the lengths from different sequencing platforms varied greatly. If there are only a very small portions of the reads are full-length transcripts, the advance of this study is limited as compared to those earlier studies.

We have added this information to the text of the revised version of the manuscript. Furthermore, a new figure (Figure 1) has also been added to the manuscript.

2. Read length distribution analysis, in addition to the average/median read lengths, will be informative to access the quality of the reads.

We have added this information to the text. Three new figures (Figures 9, 10 and 11) have been added to the manuscript. The read-length distributions for the dataset are shown in Figure 9 (reads mapped to the VACV genome), as well as Figures 10 and 11 (data aligned to the VACV and to the host genome).

3. Please discuss different read lengths using different sequencing platforms. They are unlikely all full-length transcripts. What are the advantages and disadvantages in poxvirus transcriptome analyses of the reads from different sequencing platforms?

We have added this information to the text of the revised version of the manuscript. The various sample preparation and sequencing techniques produce different read-length, read number and precision. Additionally, the various techniques produce different artifacts. There is a relatively large difference between the PacBio and ONT sequencing approaches concerning the quality of the sequencing reads: PacBio produces much fewer mismatches

and INDELs than nanopore sequencing. However, high quality reads are unnecessary for transcriptome studies if the genome of the organism is known. The various sequencing platforms recommend different cDNA production kits, which contain different enzymes and primers for the RT and PCR. The different primers and library preparation conditions may produce different artifacts; however, these can be easily filtered out if we compare the results of different methods. The PacBio MagBead loading selectively eliminates the short fragments (<1,000bp). While on the one hand, removal of incomplete cDNAs can be advantageous, at the same time, it is unfavorable, as we are unable to detect the shorter transcripts and RNA isoforms.

4. How is the average length of cellular transcripts in this study as compared to those in other studies?

A new figure (Figure 8) has been added to the manuscript. In this figure, we have compared the average aligned read-length of cellular transcripts obtained in this and other studies.

5. Specifically evaluate the reads (length, coverage, full-length or not, coverage in coding and non-coding regions, et al) of a few VACV ORFs and cellular genes with high resolution at different replication stages will help access the quality of the dataset.

A new table (Table 6) has been added to the manuscript. We have also added a heatmap illustration (Figure 12) on the expression dynamics of ten viral and ten host-specific genes.

6. What are the percentages of viral and cellular reads at each time point?

We have provided this information in the revised manuscript (see Figure 6).

7. Fig. 4. Visualization of reads coverage on VACV genome at individual time points is needed to access the quality of reads at different stages of VACV replication.

We have added an additional figure panel (Figure 5B) that presents the individual time points from Sequel and MinION sequencing. We have also retained the previous figure as Figure 5A, which includes the five different platforms and/or cDNA library preparation approaches. There are no individual time points from RSII and MinION Cap-Seq, and there is also an RSII dataset from mixed time points samples.

8. Sequencing error rates were not discussed in the manuscript.

We have added this information to the text.

Minor points.
1. Lines 49-50, is VACV a cowpox virus? From what I understand, it is not clear.

Yes, the VACV is the cowpox. We have modified the text for better clarification.

2. Table 1, what is Polya(A)(+) in the RNA sample column? Poly(A)(+)?

Yes, it is Poly(A)(+). We have corrected the misspellings.

3. Please correct some errors or typos throughout the manuscript. For example, line 116, PSB -PBS. Line 99, A detailed workflow-detailed workflows.

We have corrected the errors and typos.

4. Fig 5. 6. 7, Please label Y-axis.

Figure 5 and 6 have been changed to box plot and the Y-axis has been labeled, but Figure 7 has been removed as recommended by Reviewer #2.

Reviewer #2:

In the manuscript "Dynamic Transcriptome Profiling Dataset of Vaccinia Virus Obtained from Long-read Sequencing Techniques" by Tombácz et al., the authors describe a dataset produced by Pacbio and Nanopore sequencing of VACV, with multiple approaches taken in both sample collection and sequencing library preparation in order to profile several features of the transcriptome. The dataset is of obvious importance to the field and contains many interesting features, but there are some concerns which would need to be addressed before this manuscript is ready for publication.
Specific points:
1. It would be beneficial to include a brief statement defining "dynamic" and "static" sample collection strategies in your data description to clarify how you are using these terms. It is unclear what infection condition means in the Table 1 context, and should either be clarified or replaced by something along the lines of "sample collection strategy".

We have defined the terms 'static' and 'dynamic' in the Table legend, and replaced 'infection condition' with 'sample collection strategy' as suggested.

2. How do the samples in Table 2 (A, B, C) correlate with the sequencing run in Table 1?

An additional column has been added to Table 2. Furthermore, details from RSII sequencing have also been added.

3. Why was yield so high for the viral genome given the lack of selection? Is the level of viral transcript typically this high, and what you would expect?

We have developed a novel program recently published on Github, which was used for the reanalysis of our data in the revised manuscript: https://github.com/Szunyike/SAM-Statistic-2018
The ratio of viral transcripts is 21.9% on average in our samples. The exact ratio is dependent on the titer of the virus used for the infection, as well as on the stage of the viral life cycle at the examination period. The sequencing method affects the ratio of read counts between the virus and host cell: e.g. the MinION 1D-Seq method yields a higher amount of shorter reads compared to the PacBio Sequel technique. The VACV transcripts are relatively short compared to the host or to other large DNA viruses (such as herpesviruses and baculoviruses), which is assumed to result in the relatively high ratio of viral reads compared to the host reads in the MinION samples.

4. Tables 6 and 7 should be combined for ease of interpretation. Tables are not as useful to show read/insert length distribution as actually plotting the distribution, and box and

whiskers are inadequate. We recommend pulling out error rates, and representing instead as violin plots, or at least box plots, and include a column for "total reads" and either the count of mapped VACV reads, or percent reads mapped to VACV. Similarly, figures 5 and 6 should be combined to allow for direct comparison, and figure 7 should be removed.

Tables 6 and 7 have been combined into a single table (Table 5). Figures 5 and 6 have been changed to a box plot and they are combined into a single figure (Figure 7). Read-length distribution has also been presented in the revised manuscript (Figures 9, 10, 11 and Additional file 3). Figure 7 of the old version of the manuscript has been removed. We have provided a new figure (Figure 6) showing the percentages of viral and cellular reads at each sample.

5. Regarding alignments, it is unclear why certain sequencing were aligned to VACV and why others were aligned to both VACV and CV-1. For example, why aren't the RSII and direct RNA runs included in Table 7 and Figure 6 host mappings?

We have aligned the RSII and dRNA data to the CV-1 cell line and the statistical data has been added to Table 5 (Table 7 in the old version).

6. On your Circos plot, it would be interesting to color genes by early/intermediate/late stage. Also regarding the Circos plot - where is the dRNA data?

The Circos plot has been modified according to the recommendation: the various kinetic classes have been labeled in different colors (Figure 5A). Another picture of coverage on VACV genome at individual time points has also been added (Figure 5B) as was suggested by another Reviewer. The coverage in dRNA sequencing is very low compared to the other five (Sequel, RSII, MinION 1D cDNA, MinION 1D barcoded, and MinION Cap) approaches, and it is not visible at the scale applied for the generation of circus plot. Therefore, we have added a Shashimi plot as a part of this figure (Figure 5C) instead of adding the dRNA values to the same circus plot.

7. In Table 6, your coverage calculations seem improbable in places. For example, how could the RSII 5kb+ BluePippin run with 8 mapped reads have 42.17X coverage?

We have corrected this mistake.

8. Your statement about searching for modifications in dRNA should be amended - although 5mC is widespread in DNA, it is not as ubiquitous in RNA. Additionally, 6mA has been most extensively profiled in viruses: http://jvi.asm.org/content/91/9/e02263-16.full, and is actually quite common.

We have corrected this part of the manuscript.

9. In the 1D ONT-cDNA sequencing section, instead of telling the reader that different steps were used from the ONT protocol, I suggest describing what enzymes were used for the end-polishing. The ONT protocols are not publicly accessible, and since the preparations done on these samples were so complex, it may make sense to just say what was done.

We have added detailed information about the ONT 1D cDNA and direct RNA protocols to the revised manuscript.

10. Why not minimap2 instead of GMAP? Choice of GMAP is perfectly acceptable, but for readers it would help to explain the choice.

We have chosen GMAP because we have found it the best long-read aligner in our earlier publications [18, 19, 20, 21, 22, 23, 24]. GMAP have also produced the best alignment results in other studies [e.g. 28]. We generated the PacBio RSII dataset in 2016, when Minimap2 had not yet existed. In the beginning, Minimap2 did not support for RNA mapping (version 1: https://arxiv.org/abs/1708.01492v1). Our MinION and Sequel data are newer, and later versions of the Minimap2 program now support RNA mapping. However, our opinion is that the correct approach would be to use the same program for mapping that we had used to attain our existing data. We have added the Minimap2 program as a recommended long-read aligner to the 'Conclusion and Reuse Potential' section of the manuscript.

11. Language occasionally awkward, for example "VACV remains to be considered a weapon against potential smallpox outbreaks". Careful editing for grammar, subject-verb agreement will greatly improve readability.

The manuscript has been thoroughly edited in terms of grammar, and was proofread by a native speaker of English.

Some minor points:
1. Fastqs (unsorted by organism, just of all reads, for each run) should be hosted on gigascience for ease of access - this would be the first piece of information we would want to download.

We have uploaded the fastq.qz files to FigShare:
https://figshare.com/s/675f5f71c633473b7445

2. In findings section of the abstract, it is more useful to include the genus/species of host, and leave the accession numbers to the methods section. Additionally, it would be more useful to include Gb data produced, as well as the size of the genome, upfront.

We have modified the text as recommended.

3. In table 1, please make yes/no capitalization consistent. A label of some kind linking the workflows in Fig 1 and Fig 2 and 3 - or linking to the Table would be very helpful. Many different preparations are represented here, and parsing them in the current format is challenging at best. Perhaps some sort of encoding like ONT-cDNA-polyA_1 at the bottom of fig 3 for the relevant workflow and labeled as a column in Table 1? Additionally, please make clear for fig 3 what is dRNA and what is cDNA.

The recommended corrections have been made in Table 1. We have also made some minor modifications on Figures 3 and 4 (Figures 2 and 3 in the old version of the MS) according to the reviewer's suggestion. Abbreviations of dRNA and cDNA are now explained and we have provided a linking label between Figures 2, 3 and 4 (Figure 1, 2 and 3 in the old version of the manuscript). The labels are as follows: „Workflow A, B, C, D, E, F, G and H", and can clearly identify the different methods depicted on Figures 3 and 4 (Figures 2 and 3 in the old version of the MS).

4. Sometimes you denote 5'/3', sometimes 5′/3′, please make consistent to 5′/3′.

We have modified the text with consistent labeling of 5'/3' throughout the manuscript.

5. All of your figures should have y-axis labels and, in the case of figures 4 and 7 - a legend explaining the colors.

The rectangles are colored according to the known kinetic properties of the genes: red: early 1; green: early 2 (early-late; and yellow: postreplicative (late). Y-axis labels have been added to the figures. Figure 7 has been removed as recommended by Reviewer #2

6. Please add "availability of source code and requirements" as per https://academic.oup.com/gigascience/pages/data_note

We have added the required information to the manuscript.

7. Standard deviance should be changed to standard deviation.

We have corrected the wording as recommended.

8. The statement in line 235 "These aligned reads can be further analyzed by using different long read aligners" should be modified to "These aligned reads can be further analyzed by comparing to results of different long read aligners"

This sentence has been changed as recommended.

9. Alignment file names should be updated to a consistent format

A supplementary table (Additional file 4) has been added which explains the file names of alignments deposited in ENA paired with the names that are used in this manuscript.

10. Tables 3 and 5 should be combined.

These two tables have been combined. Additional data have also been added.

11. Table 1 and Figure 1 are redundant, consider removing Figure 1 or adding additional unique information to it.

We have provided additional information to Figure 2 (Figure 1 in the old version of the MS) according to the reviewer's recommendation: we have labeled the unique workflows (A, B, C, D, E, F, G and H) on Figure 2, which helps to identify the different methods presented in the Figures 3 and 4 (Figures 2 and 3 in the old version of the manuscript).

12. Your text mentions a "fast5.tar.gz" file for ONT dRNA, but listed in the dRNA folder of archives is just a "tar.gz" file in addition to the BAMs - please clarify that this is the same. Also, you only included the raw reads which aligned to VCAV - but it is worthwhile to include all reads as well, because your alignments might not be all-inclusive.

The files Fast5.tar.gz and tar.gz files are the same. The reads aligned to the host genome

have been deposited in the ENA.

13. What size is the tissue culture flask for the CV-1s?

We used 25 cm2 culture flasks for the propagation of CV-1 cells. This information has been added to the MS.

14. Why were 3x freeze-thaw cycles applied? What is the reasoning for that?

Freezing and thawing were carried out to help the degradation of cell membrane and nuclear envelope for more efficient isolation of RNA molecules.

Third report:
In this Data Note, the authors present full-length transcriptome data generated by third-generation single-molecule long-read sequencing technologies including PacBio (Pacific Biosciences) and ONT (Oxford Nanopore Technologies) for VACV (Vaccinia virus). Two full-length cDNA synthesis methods (Clontech SMARTer and Lexogen TeloPrime) and three platforms (PacBio RS II, PacBio Sequel and ONT MinION) were applied in this study. The authors also generated transcriptome data using ONT direct RNA sequencing technology. These data will benefit the identification of novel VACV RNA isoforms for virologists and the development and evaluation of bioinformatics tools. Below are some comments on this manuscript.

1. The authors used two strategies ("no size selection" and "Bluepippin Size Selection") for PacBio RS II platform. Why the number of ROIs generated by "no size selection" is significantly smaller than "Bluepippin Size Selection" (Table 6)?

The size-selected samples do not necessarily produce higher yields than the non-size-selected samples. In some cases, PacBio run results in low output, for example because of underloading of the SMRT Cell. This sample is a minor part of our dataset. We have labeled this sample with an asterisk (*) in Table 5 (Table 6 in the previous version of the MS) and indicated the possible cause for the yield differences in the figure legend.

2. In Tables 6 and 7, three columns "insertion frequency", "deletion frequency" and "mismatch frequency" are shown, but the authors did not describe them in "Data summary" section. More details should be included.

We have added more details to the Data summary section of the revised manuscript.

3. The grammar should be improved. For example, in Page 4 Line 81, "determine which transcripts contains certain 5'-ends and 3'-ends."; in Page 4 Line 86, "By using these techniques for cDNA production and"; Page 7 Line 140, "240 minutes were set for the RSII movie lengths, while 600 min were applied"; in Page 10 Line 191-192, "This basecaller is able identify the nucleotide sequences directly from raw sequencing data."

We have corrected the grammar.

4. In Table 2, the comma should be converted to the dot.

We have corrected this error.

5. In Page 6 Line 42-44, to the best of our knowledge, m6A is an abundant modification in mRNA. Could the authors check whether m6A or 5mC is most widespread in RNA modifications? In addition, the abbreviation should be "m6A" for RNA N6-Methyladenosine. The "6mA" is the abbreviation of DNA N6-methyladenine.

We have modified the appropriate part of the manuscript. We also have added new text and new references to the revised MS. Furthermore, we have changed the abbreviation of N6-Methyladenosine to “m6A”.

6. In Page 15 Line 293, the full name of "TSS" should be "transcription start site".

We have corrected this error.

https://giga.editorialmanager.com/l.asp?i=41827&l=SE8GCNWN

Close
