# Supplementary material for: Dynamic transcriptome profiling dataset of vaccinia virus obtained from long-read sequencing techniques
Source: Gigascience. 2018 Nov 23;7(12):giy139. doi: 10.1093/gigascience/giy139 (PMC6290886; doi:10.1093/gigascience/giy139)
Supplement: response_to_reviewer_comments_revision_1.pdf [file giy139_response_to_reviewer_comments_revision_1.pdf]

Author's Response To Reviewer Comments

Close

Dear Editor,

We would like to thank you and the two referees for the helpful comments and suggestions to our manuscript. We have made the required corrections and we hope that the manuscript will now be acceptable for publication in GigaScience. The changes have been marked with blue in the revised version of the manuscript. Our point-by-point responses to the comments are given below

Reviewer #1:

The revised manuscript was improved and addressed many concerns raised by the reviewers. Clarification of the following two points may help further improve the manuscript.

1. How are the full-length transcripts determined and defined from the total transcripts? It is not very clear in the current version.

We have clarified this issue in the revised version of the manuscript.

2. Reference #2 didn't specify VACV is a cowpox virus. In fact, most literatures indicate that VACV origin is unclear. Please clarify.

We have clarified this issue in the text and replaced reference #2 (reference #3 in the revised manuscript) with a more relevant citation.

Reviewer #2:

The authors' response to our comments and suggestions has improved this manuscript. We are generally satisfied with the manuscript edits and improvements; however, there are a few more modifications, particularly in the presentation of results, that we feel are required to make this paper acceptable for publishing.

1. In lines 261-265, the authors argue that because they have more short reads from the ONT runs, and VACV transcripts are shorter, this explains the higher ratio of viral reads to host reads in nanopore sequencing. This argument makes some sense for the comparisons to runs in which you performed size selection, where you could be excluding certain shorter VACV transcripts; however, it does not cover (for example) the Sequel runs, where you didn't. Clarification is needed, or this assumption should be removed from the text.

We have clarified this issue in the text of the revised manuscript.

2. Streamlining the manuscript would greatly improve the clarity. Many of the tables and figures have overlapping or redundant information. For example, Figures 1 and 6 are illustrating the same point. I would pick a single figure to talk about read length, one to talk

about coverage, etc. Same for tables.

We have thoroughly reorganized the structures of the figures and tables in order to improve clarity. For example, a large part of Figure 6 (Figure 4 in this version of the manuscript) has moved to the supplement as Additional file 3. Table 6 and a large part of Table 5 have also been moved to the supplement (they are now Additional files 6 and 2, respectively). However, we retained Figure 1 and a part of Figure 6 (Figure 4 in this version of the manuscript), because the other reviewer had suggested including this information in the manuscript. Figure 1 shows the ratios between the full-length and partial reads, whereas Figure 6 illustrates the ratios between the virus and host-specific reads. Figure 2 has been removed, whereas Figures 3 and 4 have been combined.

3. Figure 12 needs a legend, and a clear explanation of how expression levels were calculated.

A legend has been added to Figure 12 (Figure 10 in this version of the manuscript) and explanations have been added to outline how expression levels were calculated.

Small edits:
- Consider combining and streamlining the two sentences in lines 92-94 regarding ONT reads.

We have corrected the text as suggested.

- In Table 2 you should clarify what A/B/C are in both legend and methods, as well as clarify what the asterisk in run 2 means.

We have modified the header of this table, which now includes the requested explanations. We have also modified the text to explain rows A, B and C. The asterisk stands for a remark that is explained in the table legend. Row A in this table shows the amount of polyA(+) RNAs, however sample #2 (which is labeled with an asterisk) is the sample for random primed sequencing, for which we used rRNA-depleted RNA instead of polyA(+) RNA.

- Lines 92-98 can be broken up into 2-3 separate sentences.

We have corrected the text as suggested.

- Table 5. What do the asterisks mean?

The asterisk stands for a remark, concerning the explanation of differences between the read counts of the samples (this modification was requested by the other Reviewer). We have moved the asterisk from the first row of the table to the header of Table 5. A large part of Table 5 has been moved to Additional file 3 in the revised version of the manuscript.

- Table 6. Can be moved to supplement or removed.

We have moved this table to the supplement (it is now Additional file 6)

- Lines 345 and 346. Spell check on modification names

We have corrected the typos.

Close
