# Supplementary material for: Dynamic transcriptome profiling dataset of vaccinia virus obtained from long-read sequencing techniques
Source: Gigascience. 2018 Nov 23;7(12):giy139. doi: 10.1093/gigascience/giy139 (PMC6290886; doi:10.1093/gigascience/giy139)
Supplement: reviewer_1_report_(original_submission) -- Zhilong Yang, Ph.D — 6/18/2018 Reviewed [file giy139_reviewer_1_report_(original_submission).pdf]

Reviewer Report

Title: **Dynamic Transcriptome Profiling Dataset of Vaccinia Virus Obtained from Long-read Sequencing Techniques**

Version: **Original Submission** **Date:** 6/18/2018

Reviewer name **Zhilong Yang, Ph.D**

Reviewer Comments to Author:

Tombacz et al. used multiple long-read sequencing techniques to sequence VACV and its host cell transcripts during infection. This study obtained over one million reads. Full-length long deep-sequencing reads are critically needed in poxvirus research due to the complexity of poxvirus transcripts: pervasive transcription initiation, termination, extensive read-throughs, tightly spaced ORFs et al. Some of these issues could not be adequately addressed using sequencing technologies yielding relatively short reads. While these data likely provide useful dataset for poxvirus study, more information is required to access the quality of the data and its utilities.

Major points
1. Based on the information provided, it is not clear whether the reads (or what percentage of the reads) are full-length transcripts (although the authors claimed that they obtained full-length reads in line 91). Especially, the lengths from different sequencing platforms varied greatly. If there are only a very small portions of the reads are full-length transcripts, the advance of this study is limited as compared to those earlier studies.
2. Read length distribution analysis, in addition to the average/median read lengths, will be informative to access the quality of the reads.
3. Please discuss different read lengths using different sequencing platforms. They are unlikely all full-length transcripts. What are the advantages and disadvantages in poxvirus transcriptome analyses of the reads from different sequencing platforms?
4. How is the average length of cellular transcripts in this study as compared to those in other studies?
5. Specifically evaluate the reads (length, coverage, full-length or not, coverage in coding and non-coding regions, et al) of a few VACV ORFs and cellular genes with high resolution at different replication stages will help access the quality of the dataset.
6. What are the percentages of viral and cellular reads at each time point?
7. Fig. 4. Visualization of reads coverage on VACV genome at individual time points is needed to access the quality of reads at different stages of VACV replication.
8. Sequencing error rates were not discussed in the manuscript.

Minor points.
1. Lines 49-50, is VACV a cowpox virus? From what I understand, it is not clear.
2. Table 1, what is Polya(A)(+) in the RNA sample column? Poly(A)(+)?
3. Please correct some errors or typos throughout the manuscript. For example, line 116, PSB -PBS. Line 99, A detailed workflow-detailed workflows.
4. Fig 5. 6. 7, Please label Y-axis.

Level of Interest

Please indicate how interesting you found the manuscript: Choose an item.

Quality of Written English

Please indicate the quality of language in the manuscript: Choose an item.

Declaration of Competing Interests

Please complete a declaration of competing interests, considering the following questions:

- Have you in the past five years received reimbursements, fees, funding, or salary from an organisation that may in any way gain or lose financially from the publication of this manuscript, either now or in the future?
- Do you hold any stocks or shares in an organisation that may in any way gain or lose financially from the publication of this manuscript, either now or in the future?
- Do you hold or are you currently applying for any patents relating to the content of the manuscript?
- Have you received reimbursements, fees, funding, or salary from an organization that holds or has applied for patents relating to the content of the manuscript?
- Do you have any other financial competing interests?
- Do you have any non-financial competing interests in relation to this paper?

If you can answer no to all of the above, write 'I declare that I have no competing interests' below. If your reply is yes to any, please give details below.

I declare that I have no competing interests

I agree to the open peer review policy of the journal. I understand that my name will be included on my report to the authors and, if the manuscript is accepted for publication, my named report including any attachments I upload will be posted on the website along with the authors' responses. I agree for my report to be made available under an Open Access Creative Commons CC-BY license (http://creativecommons.org/licenses/by/4.0/). I understand that any comments which I do not wish to be included in my named report can be included as confidential comments to the editors, which will not be published.

I agree to the open peer review policy of the journal

To further support our reviewers, we have joined with Publons, where you can gain additional credit to further highlight your hard work (see: https://publons.com/journal/530/gigascience). On publication of this paper, your review will be automatically added to Publons, you can then choose whether or not to claim your Publons credit. I understand this statement. Yes
