# Supplementary material for: Dynamic transcriptome profiling dataset of vaccinia virus obtained from long-read sequencing techniques
Source: Gigascience. 2018 Nov 23;7(12):giy139. doi: 10.1093/gigascience/giy139 (PMC6290886; doi:10.1093/gigascience/giy139)
Supplement: reviewer_2_report_(original_submission) -- Rachael Workman — 6/25/2018 Reviewed [file giy139_reviewer_2_report_(original_submission).pdf]

Reviewer Report

Title: Dynamic Transcriptome Profiling Dataset of Vaccinia Virus Obtained from Long-read Sequencing Techniques

Version: Original Submission **Date:** 6/25/2018

Reviewer name Rachael Workman

. In the manuscript "Dynamic Transcriptome Profiling Dataset of Vaccinia Virus Obtained from Long-read Sequencing Techniques" by Tombácz et al., the authors describe a dataset produced by Pacbio and Nanopore sequencing of VACV, with multiple approaches taken in both sample collection and sequencing library preparation in order to profile several features of the transcriptome. The dataset is of obvious importance to the field and contains many interesting features, but there are some concerns which would need to be addressed before this manuscript is ready for publication.

Specific points:

1. It would be beneficial to include a brief statement defining "dynamic" and "static" sample collection strategies in your data description to clarify how you are using these terms. It is unclear what infection condition means in the Table 1 context, and should either be clarified or replaced by something along the lines of "sample collection strategy".
2. How do the samples in Table 2 (A, B, C) correlate with the sequencing run in Table 1?
3. Why was yield so high for the viral genome given the lack of selection? Is the level of viral transcript typically this high, and what you would expect?
4. Tables 6 and 7 should be combined for ease of interpretation. Tables are not as useful to show read/insert length distribution as actually plotting the distribution, and box and whiskers are inadequate. We recommend pulling out error rates, and representing instead as violin plots, or at least box plots, and include a column for "total reads" and either the count of mapped VACV reads, or percent reads mapped to VACV. Similarly, figures 5 and 6 should be combined to allow for direct comparison, and figure 7 should be removed.
5. Regarding alignments, it is unclear why certain sequencing were aligned to VACV and why others were aligned to both VACV and CV-1. For example, why aren't the RSII and direct RNA runs included in Table 7 and Figure 6 host mappings?
6. On your Circos plot, it would be interesting to color genes by early/intermediate/late stage. Also regarding the Circos plot - where is the dRNA data?
7. In Table 6, your coverage calculations seem improbable in places. For example, how could the RSII 5kb+ BluePippin run with 8 mapped reads have 42.17X coverage?
8. Your statement about searching for modifications in dRNA should be amended - although 5mC is widespread in DNA, it is not as ubiquitous in RNA. Additionally, 6mA has been most extensively profiled in viruses: http://jvi.asm.org/content/91/9/e02263-16.full, and is actually quite common.
9. In the 1D ONT-cDNA sequencing section, instead of telling the reader that different steps were used from the ONT protocol, I suggest describing what enzymes were used for the end-polishing. The ONT protocols are not publicly accessible, and since the preparations done on these samples were so complex, it may make sense to just say what was done.
10. Why not minimap2 instead of GMAP? Choice of GMAP is perfectly acceptable, but for readers it would help to explain the choice.
11. Language occasionally awkward,for example "VACV remains to be considered a weapon against potential smallpox outbreaks". Careful editing for grammar, subject-verb agreement will greatly improve readability.

Some minor points:
1. Fastqs (unsorted by organism, just of all reads, for each run) should be hosted on gigascience for ease of access - this would be the first piece of information we would want to download.

2. In findings section of the abstract, it is more useful to include the genus/species of host, and leave the accession numbers to the methods section. Additionally, it would be more useful to include Gb data produced, as well as the size of the genome, upfront.
3. In table 1, please make yes/no capitalization consistent. A label of some kind linking the workflows in Fig 1 and Fig 2 and 3 - or linking to the Table would be very helpful. Many different preparations are represented here, and parsing them in the current format is challenging at best. Perhaps some sort of encoding like ONT-cDNA-polyA_1 at the bottom of fig 3 for the relevant workflow and labeled as a column in Table 1? Additionally, please make clear for fig 3 what is dRNA and what is cDNA.
4. Sometimes you denote 5'/3', sometimes 5′/3′, please make consistent to 5′/3′.
5. All of your figures should have y-axis labels and, in the case of figures 4 and 7 - a legend explaining the colors.
6. Please add "availability of source code and requirements" as per https://academic.oup.com/gigascience/pages/data_note
7. Standard deviance should be changed to standard deviation.
8. The statement in line 235 "These aligned reads can be further analyzed by using different long read aligners" should be modified to "These aligned reads can be further analyzed by comparing to results of different long read aligners"
9. Alignment file names should be updated to a consistent format
10. Tables 3 and 5 should be combined.
11. Table 1 and Figure 1 are redundant, consider removing Figure 1 or adding additional unique information to it.
12. Your text mentions a "fast5.tar.gz" file for ONT dRNA, but listed in the dRNA folder of archives is just a "tar.gz" file in addition to the BAMs - please clarify that this is the same. Also, you only included the raw reads which aligned to VCAV - but it is worthwhile to include all reads as well, because your alignments might not be all-inclusive.
13. What size is the tissue culture flask for the CV-1s?
14. Why were 3x freeze-thaw cycles applied? What is the reasoning for that?

This review has been jointly carried out and signed by Winston Timp and Rachael Workman.

Level of Interest

Please indicate how interesting you found the manuscript: Choose an item.

Quality of Written English

Please indicate the quality of language in the manuscript: Choose an item.

Declaration of Competing Interests

Please complete a declaration of competing interests, considering the following questions:

- Have you in the past five years received reimbursements, fees, funding, or salary from an organisation that may in any way gain or lose financially from the publication of this manuscript, either now or in the future?
- Do you hold any stocks or shares in an organisation that may in any way gain or lose financially from the publication of this manuscript, either now or in the future?
- Do you hold or are you currently applying for any patents relating to the content of the manuscript?

- Have you received reimbursements, fees, funding, or salary from an organization that holds or has applied for patents relating to the content of the manuscript?
- Do you have any other financial competing interests?
- Do you have any non-financial competing interests in relation to this paper?

If you can answer no to all of the above, write 'I declare that I have no competing interests' below. If your reply is yes to any, please give details below.

Winston Timp and Rachael Workman have received reimbursements for speaking at conferences on both Oxford Nanopore and PacBio applications. Winston Timp has received funding and reagents from PacBio, and holds two patents licensed by Oxford Nanopore (US patent #8748091 and 8394584).

I agree to the open peer review policy of the journal. I understand that my name will be included on my report to the authors and, if the manuscript is accepted for publication, my named report including any attachments I upload will be posted on the website along with the authors' responses. I agree for my report to be made available under an Open Access Creative Commons CC-BY license (http://creativecommons.org/licenses/by/4.0/). I understand that any comments which I do not wish to be included in my named report can be included as confidential comments to the editors, which will not be published.

I agree to the open peer review policy of the journal

To further support our reviewers, we have joined with Publons, where you can gain additional credit to further highlight your hard work (see: https://publons.com/journal/530/gigascience). On publication of this paper, your review will be automatically added to Publons, you can then choose whether or not to claim your Publons credit. I understand this statement. Yes
