# Supplementary material for: Dynamic transcriptome profiling dataset of vaccinia virus obtained from long-read sequencing techniques
Source: Gigascience. 2018 Nov 23;7(12):giy139. doi: 10.1093/gigascience/giy139 (PMC6290886; doi:10.1093/gigascience/giy139)
Supplement: reviewer_2_report_revision_1 -- Rachael Workman — 10/8/2018 Reviewed [file giy139_reviewer_2_report_revision_1.pdf]

Reviewer Report

Title: **Dynamic Transcriptome Profiling Dataset of Vaccinia Virus Obtained from Long-read Sequencing Techniques**

Version: **Revision 1** **Date:** 10/8/2018

Reviewer name **Rachael Workman**

The authors' response to our comments and suggestions has improved this manuscript. We are generally satisfied with the manuscript edits and improvements; however, there are a few more modifications, particularly in the presentation of results, that we feel are required to make this paper acceptable for publishing.

1. In lines 261-265, the authors argue that because they have more short reads from the ONT runs, and VACV transcripts are shorter, this explains the higher ratio of viral reads to host reads in nanopore sequencing. This argument makes some sense for the comparisons to runs in which you performed size selection, where you could be excluding certain shorter VACV transcripts; however, it does not cover (for example) the Sequel runs, where you didn't. Clarification is needed, or this assumption should be removed from the text.

2. Streamlining the manuscript would greatly improve the clarity. Many of the the tables and figures have overlapping or redundant information. For example, Figures 1 and 6 are illustrating the same point. I would pick a single figure to talk about read length, one to talk about coverage, etc. Same for tables.

3. Figure 12 needs a legend, and a clear explanation of how expression levels were calculated.

Small edits:
Consider combining and streamlining the two sentences in lines 92-94 regarding ONT reads.

In Table 2 you should clarify what A/B/C are in both legend and methods, as well as clarify what the asterisk in run 2 means.

Lines 92-98 can be broken up into 2-3 separate sentences.

Table 5. What do the asterisks mean?

Table 6. Can be moved to supplement or removed.

Lines 345 and 346. Spell check on modification names

Level of Interest

Please indicate how interesting you found the manuscript: Choose an item.

Quality of Written English

Please indicate the quality of language in the manuscript: Choose an item.

Declaration of Competing Interests

Please complete a declaration of competing interests, considering the following questions:

- Have you in the past five years received reimbursements, fees, funding, or salary from an organisation that may in any way gain or lose financially from the publication of this manuscript, either now or in the future?
- Do you hold any stocks or shares in an organisation that may in any way gain or lose financially from the publication of this manuscript, either now or in the future?
- Do you hold or are you currently applying for any patents relating to the content of the manuscript?
- Have you received reimbursements, fees, funding, or salary from an organization that holds or has applied for patents relating to the content of the manuscript?
- Do you have any other financial competing interests?
- Do you have any non-financial competing interests in relation to this paper?

If you can answer no to all of the above, write 'I declare that I have no competing interests' below. If your reply is yes to any, please give details below.

WT holds 2 patents (US2011/0226623 A1 and US2012/0040343 A1) which have been licensed by Oxford Nanopore Technologies. WT and RW have received travel funds from Oxford Nanopore to speak at conferences.

I agree to the open peer review policy of the journal. I understand that my name will be included on my report to the authors and, if the manuscript is accepted for publication, my named report including any attachments I upload will be posted on the website along with the authors' responses. I agree for my report to be made available under an Open Access Creative Commons CC-BY license (http://creativecommons.org/licenses/by/4.0/). I understand that any comments which I do not wish to be included in my named report can be included as confidential comments to the editors, which will not be published.

I agree to the open peer review policy of the journal

To further support our reviewers, we have joined with Publons, where you can gain additional credit to further highlight your hard work (see: https://publons.com/journal/530/gigascience). On publication of this paper, your review will be automatically added to Publons, you can then choose whether or not to claim your Publons credit. I understand this statement. Yes
